# Extended Divergence-Measure Fields, the Gauss-Green Formula and Cauchy Fluxes

**DOI:** 10.1007/s00205-025-02135-7

**Published:** 2025-11-25

**Authors:** Gui-Qiang G. Chen, Christopher Irving, Monica Torres

**Affiliations:** 1 Mathematical Institute, University of Oxford, Oxford, OX2 6GG UK; 2 Faculty of Mathematics, Technical University Dortmund, Vogelpothsweg 87, Dortmund, 44227 Germany; 3 Department of Mathematics, Purdue University, West Lafayette, 47907-2067 IN USA

**Keywords:** Primary: 28C05, 26B20, 28A05, 26B12, 35L65, 35L67, Secondary: 28A75, 28A25, 26B05, 26B30, 26B40, 35D30

## Abstract

We establish the Gauss-Green formula for extended divergence-measure fields (i.e., vector-valued measures whose distributional divergences are Radon measures) over open sets. We prove that, for *almost every open set*, the normal trace is a measure supported on the boundary of the set. Moreover, for any open set, we provide a representation of the normal trace of the field over the boundary of the open set as the limit of measure-valued normal traces over the boundaries of approximating sets. Furthermore, using this theory, we extend the balance law from classical continuum physics to a general framework in which the production on any open set is measured with a Radon measure and the associated Cauchy flux is bounded by a Radon measure concentrated on the boundary of the set. We prove that there exists an extended divergence-measure field such that the Cauchy flux can be recovered through the field, *locally on almost every open set* and *globally on every open set*. Our results generalize the classical Cauchy’s Theorem (that is only valid for continuous vector fields) and extend the previous formulations of the Cauchy flux (that generate vector fields within $L^{p}$). Thereby, we establish the equivalence between entropy solutions of the multidimensional nonlinear partial differential equations of divergence form and of the mathematical formulation of physical balance laws via the Cauchy flux through the constitutive relations in the axiomatic foundation of Continuum Physics.

## Introduction

Divergence-measure fields are defined as vector-valued fields ${{\varvec{F}}}=(F_1,F_2,\cdots , F_n)$ whose distributional divergences are represented by (signed) Radon measures. An underlying connection between divergence-measure fields and hyperbolic conservation laws was first observed in [10], and such vector fields over domains with Lipschitz boundary were analyzed in [10, 11]. Since then, the analysis of divergence-measure fields has depended essentially on the regularity of ${{\varvec{F}}}$. For example, the divergence-measure fields were extensively analyzed first in $L^\infty$ in [19] and then in $L^p$ in [9]. See also [1, 4, 14, 16, 20–25, 58, 59, 62–64] and the references therein for further developments for the theory of divergence-measure fields. In this paper, we focus on the case when ${{\varvec{F}}}$ is only a vector-valued Radon measure. More precisely, we analyze *extended* divergence-measure fields which are defined as vector-valued Radon measures whose distributional divergences are Radon measures.

Our approach in this paper is motivated by the previous results in the $L^p$ setting in [9, 19]. However, the case of extended divergence-measure fields is more delicate, since ${{\varvec{F}}}$ may concentrate on lower dimensional sets (for instance, rectifiable curves). We prove that, for *almost every open set*, the normal trace of an extended divergence-measure field is a Radon measure supported on the boundary of the set. Moreover, for *every open set*, the normal trace distribution can be computed as the limit of measure-valued normal traces over the boundaries of approximating sets. Equipped with these results, we further develop a theory of Cauchy fluxes, starting from the balance law and establishing a one-to-one correspondence

$$\begin{aligned} \left\{ \text {Cauchy fluxes } {\mathcal {F}} \text { in } \Omega \right\} \, \longleftrightarrow \, \left\{ \text {Extended divergence-measure fields } {{\varvec{F}}}\text { in } \Omega \right\} \end{aligned}$$

via the normal trace. The precise statement is given in Sect. 6.2.

In the development of a theory of divergence-measure fields, one of the fundamental issues is whether a Gauss-Green formula involving these weakly differentiable vector fields can still be provided. We refer the reader to [18] for a detailed exposition on the development of this fundamental formula, starting from Lagrange (1762) and culminating with the classical formula

1.1 $$\begin{aligned} \int _{U} \phi \, \operatorname {div}{{\varvec{F}}}\,\textrm{d}x + \int _{U} \nabla \phi \cdot {{\varvec{F}}}\,\textrm{d}x = - \int _{\partial U} \phi \,{{\varvec{F}}}\cdot \nu \, \textrm{d}{\mathcal {H}}^{n-1}, \end{aligned}$$

valid for any smooth vector field ${{\varvec{F}}}$, smooth test function $\phi$, and open set *U* with smooth boundary and interior unit normal $\nu$. A first extension of this formula was achieved by Federer and De-Giorgi [28, 29, 36, 37] for the cases of Lipschitz vector fields and sets with irregular boundaries (sets of finite perimeter) by using tools of geometric-measure theory.

Further extensions of (1.1) to divergence-measure fields require a notion of normal trace on the boundary $\partial U$ of any open *U*. For the case of bounded vector fields and sets of finite perimeter, the approach in [19] consisted in constructing essentially *interior* and *exterior* approximations of the sets of finite perimeter with smooth sets and then obtaining the normal traces as the limits of classical normal traces on the smooth approximating sets, which leads to the existence of interior and exterior traces for *every * set of finite perimeter (see also [23]). This approach is consistent with applications to hyperbolic conservation laws since solutions to these equations have jumps across the shock waves (*cf*. [8, 10, 27, 45]). For a divergence-measure field whose underlying field is bounded, given any set of finite perimeter, it was shown in [19] that the normal trace is a bounded function supported on the reduced boundary of the set. For the case of an unbounded vector field, the normal trace is classical for *almost every open set*, and it was shown in [9] that the normal trace distribution on *every open set* can be computed as the limit of the classical normal traces on the boundaries of approximating sets. For unbounded vector fields, several counterexamples show that the normal trace distribution can not be represented in general as a measure supported on the boundary of the set (see for instance [9, 18, 64]). The technique that we develop in this paper in order to generalize (1.1) to the case of extended-divergence fields is the *disintegration of measures*.

As indicated earlier, divergence-measure fields arise naturally in the field of nonlinear hyperbolic conservation laws:

$$\begin{aligned} \partial _t{\textbf{u}}+ \sum _{j=1}^m \partial _{x_j} {\textbf{f}}_j({\textbf{u}})=0 \quad \text{ for } (t,x) \in {\mathbb {R}}_+ \times {\mathbb {R}}^m. \end{aligned}$$

In short form, this is

1.2 $$\begin{aligned} \partial _t{\textbf{u}}+ \operatorname {div}_x \, {\textbf{f}}({\textbf{u}})=0 \quad \text{ for } (t,x) \in {\mathbb {R}}_+ \times {\mathbb {R}}^m, \end{aligned}$$

with $n = m+1$, taking the row-wise divergence of ${\textbf{f}}({\textbf{u}}) = ({\textbf{f}}_1({\textbf{u}}),{\textbf{f}}_2({\textbf{u}}),\cdots ,{\textbf{f}}_m({\textbf{u}}))$, where ${\textbf{f}}_i^{\intercal }:{\mathbb {R}}^N \rightarrow {\mathbb {R}}^m$ for $i=1, \cdots , m$ and ${\textbf{u}} = (u_1,u_2,\cdots ,u_N)^{\intercal }$. One of the main features of (1.2) is that, no matter how smooth the initial data start with, the solution may developly singularities to become discontinuous or singular (unbounded or measure-valued). Physical relevant solutions, so-called *entropy solutions*, of (1.2) are required to be characterized by the entropy inequality

1.3 $$\begin{aligned} \partial _t\eta ({\textbf{u}}) + \operatorname {div}_x \, {\textbf{q}}({\textbf{u}}) \le 0, \end{aligned}$$

which holds in the distributional sense for any entropy-entropy flux pair $(\eta , {\textbf{q}})=(\eta , q_1,\cdots , q_m)$ (i.e., $\nabla q_i({\textbf{u}})=\nabla \eta ({\textbf{u}}){\textbf{f}}_i({\textbf{u}}), i=1,\cdots , m$) that is *convex*, i.e., $\nabla ^2\eta ({\textbf{u}})\ge 0$, for which the field $(\eta ({\textbf{u}}(t,x)), {\textbf{q}}({\textbf{u}}(t,x)))$ is defined. From (1.3), it follows that there is a non-negative measure $\sigma _{\eta }$ such that

$$\begin{aligned} - \operatorname {div}_{t,x} (\eta ({\textbf{u}}(t,x)), {\textbf{q}}({\textbf{u}}(t,x)))= \sigma _{\eta }. \end{aligned}$$

Therefore, $(\eta ({\textbf{u}}(t,x)), {\textbf{q}}({\textbf{u}}(t,x)))$ is an extended divergence-measure field. To study the jumps of entropy solutions across shock waves, we want to obtain the interior and exterior normal traces of entropy-entropy flux fields of the solutions on the shock waves by approximating them with smooth surfaces.

The theory of divergence-measure fields in $L^\infty$ has been applied to the analysis of properties of entropy solutions of nonlinear hyperbolic conservation laws (1.2); see for instance [10, 17, 18] and the references cited therein, as well as Sect. 12 below for the details. Divergence-measure fields also appear in many other areas of analysis, including the study of prescribed mean curvature equations, the 1-Laplacian, the continuity equation, and related topics. We refer to [26, 44, 46–48, 55–57] and the references therein. The theory of divergence-measure fields (such as normal traces, Gauss-Green formulas, and product rules, among others) provide a mathematical foundation for developing new techniques and tools for entropy methods, measure-theoretic analysis, partial differential equations, free boundary problems, and related areas.

Furthermore, there are underlying intrinsic connections between divergence-measure fields and the Cauchy fluxes for the physical balance laws in Continuum Physics. In this paper, we further analyze such connections and present how the Cauchy fluxes can be represented by extended divergence-measure fields. The origin of the study of Cauchy fluxes dates back to the fundamental paper by Cauchy [6] who considered the balance law in Classical Physics:

1.4 $$\begin{aligned} \int _{U} p(x) \, \text {d}x = \int _{\partial U} f(x, \nu (x)) \,\text {d}{\mathcal {H}}^{n-1}(x) \quad \,\, \text { for } \text { any } U \Subset \Omega .\end{aligned}$$

Here, $\Omega$ is a bounded open set, $\nu$ is the interior unit normal, the production *p*(*x*) is a bounded function in $x \in \Omega$, and the *density function* $f(x,\nu )$ is continuous in $x \in \Omega$. The balance law postulates that the production of a quantity in any bounded open set $U \Subset \Omega$ is balanced by the flux of this quantity through $\partial U$. It was shown in [6] that there exists a continuous vector field ${{\varvec{F}}}$ such that

1.5 $$\begin{aligned} f(x, \nu )= {{\varvec{F}}}(x) \cdot \nu . \end{aligned}$$

From classical continuum physics, it follows that the object of study should be the total flux across a surface *S* contained in $\partial U$, that is,

$$\begin{aligned} {\mathcal {F}}(S)= \int _{S} f(x, \nu (x)) \,\textrm{d}{\mathcal {H}}^{n-1}(x). \end{aligned}$$

In this paper, we formulate the conditions on the Cauchy flux ${\mathcal {F}}$ which guarantee the existence of an extended divergence-measure field ${{\varvec{F}}}$ with the property that ${\mathcal {F}}(S)$ can be recovered through the normal trace of ${{\varvec{F}}}$ on open sets. This recovery is global for *every* open set, and local for *almost every open set*. Since the fundamental work of Cauchy [6], some important developments have been made on the problem of removing the continuity assumption; see [9, 19, 31, 43, 52, 59–63, 67] and the references cited therein. We refer the reader to Sect. 6.1 for a more detailed description of the history of Cauchy fluxes and contributions of the aforementioned references.

Classically, the derivation of nonlinear system (1.2) of conservation laws is carried out directly from the balance law (1.4) under the assumption that the vector field is classically differentiable. This procedure is not rigorous, since the solutions to (1.2) may be discontinuous, or even measure-valued, in general. We seek to rigorously derive (1.5) under a general framework with much weaker assumptions. We consider the generalized balance law

$$\begin{aligned} \sigma (U) = {\mathcal {F}}(\partial U) \quad \text { for any } U \Subset \Omega , \end{aligned}$$

where the production $\sigma$ is a Radon measure and the Cauchy Flux ${\mathcal {F}}$ is bounded by a Radon measure concentrated on the boundary $\partial U$ of *U* (see Definition 6.4). Moreover, we seek to *recover the flux locally* as

1.6 $$\begin{aligned} {\mathcal {F}}_U(S) = ( {{\varvec{F}}}\cdot \nu )_{\partial U}(S), \end{aligned}$$

valid for *almost all* open $U \Subset \Omega$ and all Borel $S \subset \partial U$, where the right-hand side is the normal trace of ${{\varvec{F}}}$ to be made precise in Definition 2.4. An extensive analysis has been made when the underlying field is bounded or lies in $L^p$, however a complete treatment in the measure-valued case has remained open, which is one of our motivations.

In this paper, we propose a general formulation for the Cauchy flux in Definition 6.4, which encapsulates the case of measure-valued fields. One of our main results, Theorem 6.6, states that the balance law, together with the conditions imposed on the Cauchy flux, implies the existence of an extended divergence-measure field ${{\varvec{F}}}$ such that the Cauchy flux can be locally recovered through the normal trace of ${{\varvec{F}}}$ on the boundary of almost every open set in the sense of (1.6).

The outline of this paper is as follows: In Sect. 2, we introduce the distributional normal trace and a product rule for extended divergence-measure fields. We show in Sect. 3, more precisely in Theorem 3.3, that the disintegration of measures yields the Gauss-Green formulas for extended divergence-measure fields. Further properties of this disintegration are studied in Sect. 4. In particular, Theorem 4.1 is a coarea-type formula for divergence-measure fields. In Sect.  5, the localization properties of the normal trace are developed, which further motivate our notion of the Cauchy flux in the sequel. In Sects.  6–9, we are devoted to the analysis of the Cauchy flux and the proof of Theorem 6.6. The extensions of the aforementioned results to general open sets $U \subset \Omega$ are analyzed in Sect. 10. In Sect. 11, we discuss the solvability of the equation $-\operatorname {div}{{\varvec{F}}}= \sigma$. Finally, in Sect.  12, we apply the theory of Cauchy fluxes to study the equivalence between entropy solutions of the multidimensional nonlinear partial differential equations of balance laws and of the mathematical formulation of physical balance laws through the constitutive relations in the axiomatic foundation of Continuum Physics.

## Extended Divergence-Measure Fields and Distributional Normal Traces

We start this section by introducing some basic notation and recalling some properties of Radon measures, and then introduce divergence-measure fields and the normal traces.

### Preliminary Notions

Throughout this paper, we work with open subsets $\Omega \subset {\mathbb {R}}^n$, with the standing assumption that $n \ge 2$ unless otherwise specified. We use $B_r(x)$ to denote an open ball of radius $r>0$ centered at $x \in {\mathbb {R}}^n$; more generally, we write $B_r(A) = \bigcup _{a \in A} B_r(a)$ for a set $A \subset {\mathbb {R}}^n$. For any set $A \subset {\mathbb {R}}^n$, denote the characteristic function of *A* by $\mathbbm {1}_A$, and use $A^{\textrm{c}} = {\mathbb {R}}^n \setminus A$ to denote the complement of *A*. We also write $A \Subset \Omega$ if ${\overline{A}}$ is compact and ${{\overline{A}}} \subset \Omega$.

For any space $X(\Omega ,{\mathbb {R}}^n)$ of vector function fields on $\Omega \subset {\mathbb {R}}^n$, denote by $X_{{\textrm{loc}}}(\Omega ,\mathbb R^n)$ the space of vector function fields *f* for which $f |_{\Omega '} \in X(\Omega ', {\mathbb {R}}^{n})$ for all $\Omega ' \Subset \Omega$, and by $X_{\textrm{c}}(\Omega ,{\mathbb {R}}^n)$ the space of vector function fields $f \in X(\Omega ,{\mathbb {R}}^n)$ which are compactly supported in $\Omega$. We write $X(\Omega )$ for the space of scalar functions and define $X_{{\textrm{loc}}}(\Omega )$ and $X_{\textrm{c}}(\Omega )$ analogously.

Denote by $\textrm{Lip}_{\textrm{b}}(\Omega )$ the space of Lipschitz-continuous functions that are bounded in the sense that

$$\begin{aligned} \Vert \phi \Vert _{\textrm{Lip}_{\textrm{b}}(\Omega )} := \Vert \phi \Vert _{L^{\infty }(\Omega )} + \textrm{Lip}(\phi ) < \infty . \end{aligned}$$

Rademacher’s Theorem implies that $\textrm{Lip}_{\textrm{b}}(\Omega ) \subset {W}^{1,\infty }(\Omega )$; however, this inclusion may be strict for a general open set $\Omega$.

For any open set $U \subset \Omega$ and $\varepsilon >0$, throughout the paper, we use the notation

$$\begin{aligned} U^{\varepsilon } := \{ x \in U\,:\,\textrm{dist}(x,\partial U) > \varepsilon \}. \end{aligned}$$

We also set that $U^0 = U$ and

$$\begin{aligned} U^{-\varepsilon } := \{ x \in {\mathbb {R}}^n \,:\,\textrm{dist}(x, U) < \varepsilon \}. \end{aligned}$$

We see that $\partial U^{\varepsilon }$ are the *interior approximations* of $\partial U$, while $\partial U^{-\varepsilon }$ give *exterior approximations*. We often abbreviate the distance function $d(x) = d_U(x) = \textrm{dist}(x,\partial U)$ if the underlying set is clear from the context.

By a *standard mollifier* we mean a non-negative function $\rho \in C^{\infty }_{\textrm{c}}({\mathbb {R}}^n)$, supported in the unit ball, such that $\int _{{\mathbb {R}}^n} \rho (x) \,\textrm{d}x = 1$. For $\delta >0$, we set $\rho _{\delta }(x) = \delta ^{-n} \rho (\frac{x}{\delta })$ and, for $f \in L^1_{{\textrm{loc}}}(\Omega )$, we often write $f_{\delta } = f *\rho _{\delta }$ for the mollification defined in $\Omega ^{\delta }$.

For any open set $\Omega \subset {\mathbb {R}}^{n}$, denote ${\mathcal {M}} (\Omega , {\mathbb {R}}^{n})$ as the space of all finite vector-valued Radon measures on $\Omega$, and ${\mathcal {M}}(\Omega )$ as the space of finite signed Radon measures. If $\mu _k$ is a sequence of vector-valued Radon measures in ${\mathcal {M}} (\Omega , {\mathbb {R}}^{n})$, we use the notation
to denote that the sequence converges to $\mu$ in the weak*–topology. If $\mu$ is a Radon measure, denote its total variation as $|\mu |$. We say that $\mu$ is concentrated in a set $E \subset {\mathbb {R}}^{n}$ if $\left| {\mu }\right| ({\mathbb {R}}^{n} {\setminus } E)=0$. The support of $\mu$, denoted as $\operatorname {spt}(\mu )$, is the intersection of all closed sets *E* such that $\mu$ is concentrated on *E*. In particular,

$$\begin{aligned} {\mathbb {R}}^{n} \setminus \operatorname {spt}( \mu )= \{ x \in {\mathbb {R}}^n\,:\,\left| {\mu }\right| (B_r(x))=0\, \text { for some } r>0 \}. \end{aligned}$$

Let $\mu$ and $\lambda$ be non-negative Radon measures on ${\mathcal {M}}_{{\textrm{loc}}}(\Omega )$. Define $D_{\mu }^+ \lambda : \operatorname {spt}(\mu ) \rightarrow [0, \infty ]$ and $D_{\mu }^-\lambda : \operatorname {spt}(\mu ) \rightarrow [0, \infty ]$ as

$$\begin{aligned} D_{\mu }^+ \lambda (x):= \limsup _{r \rightarrow 0} \frac{\lambda (B_r(x))}{\mu (B_r(x))}, \quad D_{\mu }^- \lambda (x):= \liminf _{r \rightarrow 0} \frac{\lambda (B_r(x))}{\mu (B_r(x))} \qquad \text { for } x \in \operatorname {spt}(\mu ). \end{aligned}$$

If $D_{\mu }^+ \lambda (x)=D_{\mu }^- \lambda (x)$, we denote this value as $D_{\mu } \lambda (x)$. The function $D_{\mu } \lambda (x)$ is called the $\mu$-density of $\lambda$ at *x*. We now recall the well-known Lebesgue-Besicovitch Differentiation Theorem (see for instance [2, §2.4] and [34, §1.6]) which states that

$$\begin{aligned} \mu (\{ x: D_{\mu }^- \lambda (x) < D_{\mu }^+ \lambda (x) \}) =0, \quad \mu (\{ x: D_{\mu }^{+} \lambda (x) = \infty \}) =0, \end{aligned}$$

that is, $D_{\mu } \lambda$ is defined and finite $\mu$–*a.e.*  on ${\mathbb {R}}^{n}$.

Moreover, $D_{\mu } \lambda$ is Borel measurable and locally $\mu$-integrable, and satisfies that

$$\begin{aligned} \lambda = (D_{\mu } \lambda )\, \mu + \lambda _{\text {sing}}. \end{aligned}$$

Here the Radon measure $\lambda _{\text {sing}}$ is defined as
with
which satisfies $\mu (Y) = 0$. We will make frequent use of the following property:

#### Lemma 2.1

Let $\mu$ and $\lambda$ be non-negative Radon measures on $\Omega$. Then $D_{\mu } \lambda _{\textrm{sing}} (x)=0$ for $\mu$–*a.e.* $\,x \in \Omega$.

#### Proof

Let  as above. Consider the decomposition

$$\begin{aligned} \lambda _{\textrm{sing}} = (D_{\mu }\lambda _{\textrm{sing}})\, \mu + (\lambda _{\textrm{sing}})_{\textrm{sing}}. \end{aligned}$$

Since all the measures are non-negative and $\lambda _{\textrm{sing}}({\mathbb {R}}^n \setminus Y) = (\lambda _{\textrm{sing}})_{\textrm{sing}}({\mathbb {R}}^n \setminus Y)=0$, it follows that

$$\begin{aligned} \int _{{\mathbb {R}}^n \setminus Y} (D_{\mu }\lambda _{\textrm{sing}})(x) \,\textrm{d}\mu (x) = 0, \end{aligned}$$

so that $D_{\mu }\lambda _{\textrm{sing}}(x) = 0$ for $\mu$–*a.e.* $x \in {\mathbb {R}}^n \setminus Y$. Since *Y* is $\mu$-null, the conclusion follows. $\square$

The above extends to the case when $\lambda$ is a signed Radon measure by considering the Jordan decomposition $\lambda = \lambda ^+ - \lambda ^-$ with $\lambda ^+$ and $\lambda ^-$ non-negative Radon measures (see [2, §1.1]). Working componentwise, we can also allow for vector-valued measures.

#### Lemma 2.2

Let $\mu$ and $\lambda$ be Radon measures on an open set $\Omega \subset {\mathbb {R}}^n$. Suppose that ${\mathcal {A}}$ is a family of Borel subsets which is closed under intersections and generates the Borel $\sigma$-algebra. If $\mu (A) = \lambda (A)$ for all $A \in \mathcal A$, then $\mu =\lambda$ as measures.

This is shown in [2, Proposition 1.8] by noting that any Radon measure is $\sigma$-finite. While the aforementioned proposition is only stated for non-negative measures, an inspection of the proof reveals that the result also holds for signed and vector-valued measures.

### Divergence-Measure Fields and Normal Traces

We are now ready to introduce our central notions of interest:

#### Definition 2.3

If $${{\varvec{F}}}\in {\mathcal {M}}(\Omega , {\mathbb {R}}^n)$$, define

$$\begin{aligned} |\operatorname {div}{{\varvec{F}}}|(\Omega ):= \sup \Big \{ \int _{\Omega } \nabla \varphi \cdot \, \textrm{d}{{\varvec{F}}}\,:\, \varphi \in C_{\textrm{c}}^1(\Omega ),\, |\varphi |\le 1\Big \}. \end{aligned}$$

We say that ${{\varvec{F}}}$ is an extended divergence-measure field over $\Omega$ if ${{\varvec{F}}}\in {\mathcal {M}}(\Omega , {\mathbb {R}}^n)$ and $|\operatorname {div}{{\varvec{F}}}|(\Omega ) < \infty$. In this case, the Riesz Representation Theorem implies that $\operatorname {div}{{\varvec{F}}}\in {\mathcal {M}} (\Omega )$ and the total variation measure $\left| {\operatorname {div}{{\varvec{F}}}}\right|$ is computed as

$$\begin{aligned} |\textrm{div}\,{{\varvec{F}}}|(A) = \sup \Big \{ \int _{\Omega } \nabla \varphi \cdot \, \text {d}{{\varvec{F}}}: \, \varphi \in C_{\text {c}}^1(A), \, |\varphi |\le 1 \Big \} \qquad \text { for } \text { every } \text { open } \text { set } A \subset \Omega , \end{aligned}$$

and $\left| {\operatorname {div}{{\varvec{F}}}}\right| (E)= \inf \{ \left| {\operatorname {div}{{\varvec{F}}}}\right| (A): A \text { open set}, \, A \supset E\}$ for arbitrary measurable sets *E*.

If ${{\varvec{F}}}\in L^{p}(\Omega , {\mathbb {R}}^n)$ for $1 \le p \le \infty$, then we may view ${{\varvec{F}}}$ as a measure in the sense:

2.1 $$\begin{aligned} \int _{\Omega } \nabla \varphi \cdot \, \textrm{d}{{\varvec{F}}}= \int _{\Omega } \nabla \varphi \cdot {{\varvec{F}}}\,\textrm{d}x. \end{aligned}$$

The spaces of all $L^{p}$-divergence-measure fields and extended divergence-measure fields are denoted as $\mathcal{D}\mathcal{M}^p(\Omega )$ and $\mathcal{D}\mathcal{M}^{\text {ext}}(\Omega )$, respectively. They are Banach spaces with norms given respectively by

$$\begin{aligned} \left\Vert {{\varvec{F}}}\right\Vert _{\mathcal{D}\mathcal{M}^p(\Omega )}= \left\Vert {{\varvec{F}}}\right\Vert _{L^p(\Omega ,{\mathbb {R}}^n)} + |\operatorname {div}{{\varvec{F}}}|(\Omega ) \end{aligned}$$

and

$$\begin{aligned} \left\Vert {{\varvec{F}}}\right\Vert _{\mathcal{D}\mathcal{M}^{\text {ext}}(\Omega )}= |{{\varvec{F}}}|(\Omega ) + |\operatorname {div}{{\varvec{F}}}|(\Omega ). \end{aligned}$$

Since the distributional divergences $\left\langle \operatorname {div}{{\varvec{F}}},\,\cdot \, \right\rangle$ of these vector fields are measures, we see that, for any $\varphi \in C_{\textrm{c}}^{\infty }(\Omega )$,

$$\begin{aligned} \left\langle \operatorname {div}{{\varvec{F}}},\varphi \right\rangle = \int _{\Omega } \varphi \, \textrm{d}(\operatorname {div}{{\varvec{F}}}) =- \int _{\Omega } \nabla \varphi \cdot {{\varvec{F}}}\, \textrm{d}x \quad \text{ for } {{\varvec{F}}}\in L^p(\Omega , {\mathbb {R}}^n), \end{aligned}$$

and

$$\begin{aligned} \left\langle \operatorname {div}{{\varvec{F}}},\varphi \right\rangle = \int _{\Omega } \varphi \, \textrm{d}(\operatorname {div}{{\varvec{F}}}) =- \int _{\Omega } \nabla \varphi \cdot \,\textrm{d}{{\varvec{F}}}\quad \text{ for } {{\varvec{F}}}\in {\mathcal {M}}(\Omega , {\mathbb {R}}^n). \end{aligned}$$

In what follows, we drop the term “extended” and refer to fields ${{\varvec{F}}}\in \mathcal{D}\mathcal{M}^{{{\,\textrm{ext}\,}}}(\Omega )$ simply as divergence-measure fields. For such fields, we can make sense of its normal trace in the distributional sense.

#### Definition 2.4

Let ${{\varvec{F}}}\in \mathcal{D}\mathcal{M}^{\text {ext}}(\Omega )$, and let $E \Subset \Omega$ be a Borel set. The *normal trace* of ${{\varvec{F}}}$ on the boundary of *E* is defined as

2.2 $$\begin{aligned} \langle {{\varvec{F}}}\cdot \nu , \,\phi \rangle _{\partial E} := -\int _{E} \nabla \phi \cdot \textrm{d}{{\varvec{F}}}- \int _{E} \phi \,\textrm{d}(\operatorname {div}{{\varvec{F}}}) \quad \text{ for } \phi \in C_{\textrm{c}}^1({\Omega }). \end{aligned}$$

Equivalently, we can write (2.2) as an equality of distributions:

2.3 $$\begin{aligned} \langle {{\varvec{F}}}\cdot \nu , \,\cdot \,\rangle _{\partial E} = \operatorname {div}(\mathbbm {1}_E {{\varvec{F}}})-\mathbbm {1}_E \operatorname {div}{{\varvec{F}}}\quad \text {in } {\mathcal {D}}'(\Omega ). \end{aligned}$$

If the distribution (2.3) can be identified as a measure in $\Omega$, then we denote this measure by $({{\varvec{F}}}\cdot \nu )_{\partial E}$.

Note that the integrals in (2.2) are well-defined for Borel sets *E*, since ${{\varvec{F}}}$ and $\operatorname {div}{{\varvec{F}}}$ are Radon measures on $\Omega$. In the case of a field ${{\varvec{F}}}\in \mathcal{D}\mathcal{M}^p(\Omega )$, the normal trace is defined by identifying ${{\varvec{F}}}$ with the vector-valued Radon measure ${{\varvec{F}}}{\mathcal {L}}^n$ as in (2.1).

#### Remark 2.5

We have opted to define the normal trace as a distribution in $\Omega$; while one can more generally consider the normal trace as a distribution on ${\mathbb {R}}^n$, we will postpone this discussion to Sect. 10. Moreover, compared to the prior works such as [9, 11, 59, 64], our definition differs by a minus sign, which corresponds to the use of the *interior* unit normal $\nu$ in the classical case.

#### Lemma 2.6

For ${{\varvec{F}}}\in \mathcal{D}\mathcal{M}^{{{\,\textrm{ext}\,}}}(\Omega )$ and any Borel set $E \Subset \Omega$, the normal trace $\langle {{\varvec{F}}}\cdot \nu , \,\cdot \,\rangle _{\partial E}$ is represented by a measure on $\Omega$ if and only if

$$\begin{aligned} \mathbbm {1}_E {{\varvec{F}}}\in \mathcal{D}\mathcal{M}^{{{\,\textrm{ext}\,}}}(\Omega ). \end{aligned}$$

#### Proof

 By definition of the normal trace, we have

$$\begin{aligned} \langle {{\varvec{F}}}\cdot \nu ,\,\cdot \,\rangle _{\partial E} = \operatorname {div}(\mathbbm {1}_E{{\varvec{F}}}) -\mathbbm {1}_E\,\operatorname {div}{{\varvec{F}}}\end{aligned}$$

as distributions in $\Omega$. Since $\mathbbm {1}_E\operatorname {div}{{\varvec{F}}}$ is a Radon measure on $\Omega$, we see that the normal trace is a measure if and only if $\operatorname {div}(\mathbbm {1}_E{{\varvec{F}}})$ is a measure. This precisely corresponds to imposing that $\mathbbm {1}_E {{\varvec{F}}}\in \mathcal{D}\mathcal{M}^{{{\,\textrm{ext}\,}}}(\Omega )$. $\square$

While we can view the normal trace as a distribution in $\Omega$, analogously to the classical case, one may wonder if it can be represented by a measure on $\partial E$, or equivalently as a distribution of order zero. While counterexamples show this need not hold in general (see [64, Example 2.5]), one can expand the allowed test functions $\phi$ in the definition of normal traces. This is obtained from the following product rule for $\mathcal{D}\mathcal{M}^{{{\,\textrm{ext}\,}}}$–fields due to Šilhavý [64] based on the notion of *pairings* introduced by Anzelotti [3] (also see [10, 11]). Far-reaching generalizations of this pairing have recently been obtained by Comi, De Cicco, and Scilla [20].

#### Theorem 2.7

If ${{\varvec{F}}}\in \mathcal {D}\mathcal {M}^{\textrm{ext}}(\Omega )$ and $\phi \in {W}^{1,\infty }(\Omega )$, then $\phi {{\varvec{F}}}\in {\mathcal {D}}{\mathcal {M}}^\textrm{ext}(\Omega )$ and

$$\begin{aligned} \operatorname {div}(\phi {{\varvec{F}}}) = \phi \,\operatorname {div}{{\varvec{F}}}+ \overline{\nabla \phi \cdot {{\varvec{F}}}}, \end{aligned}$$

where $\overline{\nabla \phi \cdot {{\varvec{F}}}}$ is a signed measure on $\Omega$ characterized by

2.4 $$\begin{aligned} \int _{\Omega } \psi \, \textrm{d}\overline{\nabla \phi \cdot {{\varvec{F}}}} = \lim _{\delta \rightarrow 0} \int _{\Omega } \psi \nabla \phi _{\delta } \cdot \textrm{d}{{\varvec{F}}}\quad \mathrm{for \,any }\,\psi \in C_{\textrm{c}}(\Omega ) , \end{aligned}$$

and satisfies the estimate

2.5 $$\begin{aligned} |\overline{\nabla \phi \cdot {{\varvec{F}}}} |\le \Vert \nabla \phi \Vert _{L^{\infty }(\Omega )} |{{\varvec{F}}}|\end{aligned}$$

as measures in $\Omega$.

#### Proof

 Let $\rho _{\delta }$ be a standard mollifer, and set $\phi _{\delta } = \phi *\rho _{\delta }$. We first claim that

2.6 $$\begin{aligned} \operatorname {div}(\phi _{\delta } {{\varvec{F}}}) = \phi _{\delta } \, \operatorname {div}{{\varvec{F}}}+ \nabla \phi _{\delta } \cdot {{\varvec{F}}}\quad \text{ in } \Omega ^{\delta }, \end{aligned}$$

and notice that the right-hand side is well-defined since both terms are a product of a Borel measure with a continuous function. To see this, we mollify the field ${{\varvec{F}}}$ by ${{\varvec{F}}}_{\varepsilon } = {{\varvec{F}}}*\rho _{\varepsilon }$, so that the classical product rule

2.7 $$\begin{aligned} \operatorname {div}(\phi _{\delta } {{\varvec{F}}}_{\varepsilon })= \phi _{\delta }\, \operatorname {div}{{\varvec{F}}}_{\varepsilon } + \nabla \phi _{\delta } \cdot {{\varvec{F}}}_{\varepsilon } \end{aligned}$$

holds in $\Omega ^{\delta }$ for $0<\varepsilon <\delta$. Let $\psi \in C_{\textrm{c}}(\Omega )$ be supported in $\Omega ^{\delta }$. Since $\operatorname {div}{{\varvec{F}}}_{\varepsilon } = (\operatorname {div}{{\varvec{F}}}) *\rho _{\varepsilon }$, $(\psi \phi _{\delta }) \in C_{\textrm{c}}(\Omega )$, and the mollifications converge weakly ${}^{*}$, we have

$$\begin{aligned} \lim _{\varepsilon \rightarrow 0} \int _{\Omega } \psi \phi _{\delta } \, \operatorname {div}{{\varvec{F}}}_{\varepsilon } \,\textrm{d}x = \int _{\Omega } \psi \phi _{\delta } \,\textrm{d}(\operatorname {div}{{\varvec{F}}}). \end{aligned}$$

In particular,  as measures in $\Omega ^{\delta }$. Similarly,  in $\Omega ^{\delta }$. Thus, since  as distributions in $\Omega ^{\delta }$ and this limit is unique, sending $\varepsilon \rightarrow 0$ in (2.7) gives that $\operatorname {div}(\phi _{\delta }{{\varvec{F}}})$ is a measure on $\Omega ^{\delta }$ given by (2.6) as claimed.

We now send $\delta \rightarrow 0$. By continuity of $\phi$, we see that $\phi _{\delta } \rightarrow \phi$ uniformly in $\Omega ^{\delta _0}$ for each $\delta _0>0$, which implies that  in each $\Omega ^{\delta _0}$. Also, since $\phi$ is Lipschitz, we now show that the second term in (2.6) is uniformly bounded in $\delta < \delta _0$:

2.8 $$\begin{aligned} \int _{\Omega ^{\delta _0}}|\nabla \phi _{\delta }|\, \textrm{d}|{{\varvec{F}}}|\le \left\Vert \nabla \phi _{\delta }\right\Vert _{L^{\infty }(\Omega ^{\delta _0})}|{{\varvec{F}}}|(\Omega ^{\delta _0}) \le \left\Vert \nabla \phi \right\Vert _{L^{\infty }(\Omega )} |{{\varvec{F}}}|(\Omega ) < \infty . \end{aligned}$$

Thus, for any $\delta _k \rightarrow 0$, we can find a further subsequence $\delta _{k_m} \rightarrow 0$ for which $\nabla \phi _{\delta _{k_m}}\!\!\cdot {{\varvec{F}}}$ converges weakly ${}^*$ in $\Omega ^{\delta _0}$ to some limiting measure as $m \rightarrow \infty$. Moreover, for each $\delta _0>0$, we have
2.9as distributions in $\Omega ^{\delta _0}$, so the limiting measure is uniquely determined in ${\mathcal {D}}'(\Omega ^{\delta _0})$ for each $\delta _0>0$. Thus, we deduce the existence of a Radon measure in $\Omega$, denoted as $\overline{\nabla \phi \cdot {{\varvec{F}}}}$, such that
for each $\delta _0>0$. Then we can upgrade the convergence in (2.9) to hold weakly ${}^{*}$ as measures, and conclude that $\operatorname {div}(\phi {{\varvec{F}}})$ is a measure in $\Omega$ given by

$$\begin{aligned} \operatorname {div}(\phi {{\varvec{F}}})= \phi \,\operatorname {div}{{\varvec{F}}}+ \overline{\nabla \phi \cdot {{\varvec{F}}}}. \end{aligned}$$

Finally, to show (2.5), we use (2.8) to estimate

$$\begin{aligned} \left| \int _{\Omega ^{\delta _0}} \psi \, \nabla \phi _{\delta } \cdot \text {d}{{\varvec{F}}}\right| \le \left\| \nabla \phi \right\| _{L^{\infty }(\Omega )} \int _{\Omega ^{\delta _0}} \psi \,\text {d}|{{\varvec{F}}}|\quad \,\, \text { for } \text { any } \psi \in C_{\text {c}}(\Omega ^{\delta _0}) \text { and } 0<\delta <\delta _0. \end{aligned}$$

Sending $\delta \rightarrow 0$ and noting that $\psi$ is arbitrary, we deduce that
as measures. Since this estimate is uniform in $\delta _0$, it holds in $\Omega$. $\square$

#### Remark 2.8

 As the proof illustrates, if $\phi \in C^{1}({{\overline{\Omega }}})$, the product rule reduces to

2.10 $$\begin{aligned} \operatorname {div}(\phi {{\varvec{F}}})= \phi \, \operatorname {div}{{\varvec{F}}}+ \nabla \phi \cdot {{\varvec{F}}}, \end{aligned}$$

where the right-hand side can be understood classically via multiplying a Radon measure by a continuous function. However, if $\phi$ is merely Lipschitz, then $\nabla \phi$ is only defined almost everywhere. Since the measure ${{\varvec{F}}}$ may concentrate on the points of non-differentiablity, it is necessary to understand the product via a suitable pairing. Moreover, if $\phi \in C^1_{\textrm{c}}(\Omega )$, we apply (2.10) to write the normal trace as

$$\begin{aligned} \langle {{\varvec{F}}}\cdot \nu , \,\phi \rangle _{\partial E} = - \operatorname {div}(\phi {{\varvec{F}}})(E) \quad \text {for any } E \Subset \Omega \text { Borel}. \end{aligned}$$

We observe that, owing to Theorem 2.7, the right-hand side is well defined even when $\phi \in {W}^{1,\infty }(\Omega )$. This leads to the following corollary:

#### Corollary 2.9

Let ${{\varvec{F}}}\in \mathcal{D}\mathcal{M}^{{{\,\textrm{ext}\,}}}(\Omega )$, and let $E \Subset \Omega$ be a Borel set. Then the normal trace extends to a bounded linear functional on ${W}^{1,\infty }(\Omega )$ by setting

$$\begin{aligned} \langle {{\varvec{F}}}\cdot \nu , \,\phi \rangle _{\partial E} = -\operatorname {div}(\phi {{\varvec{F}}})(E) = -\int _{E} \phi \,\textrm{d}(\operatorname {div}{{\varvec{F}}}) - \int _{E} \textrm{d}\overline{\nabla \phi \cdot {{\varvec{F}}}} \end{aligned}$$

for any $$\phi \in {W}^{1,\infty }(\Omega )$$.

#### Proof

  By the product rule (Theorem 2.7), the extension is well-defined and agrees with Definition 2.4 when $\phi \in C^1_{\textrm{c}}(\Omega )$. Moreover, by linearity of the distributional divergence, we see that $\langle {{\varvec{F}}}\cdot \nu , \,\cdot \,\rangle _{\partial E}$ is linear; its boundedness follows from the estimate

$$\begin{aligned} \begin{aligned} |\langle {{\varvec{F}}}\cdot \nu , \,\phi \rangle _{\partial E}|&\le \int _{\Omega } |\phi |\,\textrm{d}|\operatorname {div}{{\varvec{F}}}|+ |\overline{\nabla \phi \cdot {{\varvec{F}}}}|(\Omega ) \\&\le \Vert \phi \Vert _{L^{\infty }(\Omega )} |\operatorname {div}{{\varvec{F}}}|(\Omega ) + \Vert \nabla \phi \Vert _{L^{\infty }(\Omega )} |{{\varvec{F}}}|(\Omega )\\&\le \left\Vert \phi \right\Vert _{{W}^{1,\infty }(\Omega )} \left\Vert {{\varvec{F}}}\right\Vert _{\mathcal{D}\mathcal{M}^{{{\,\textrm{ext}\,}}}(\Omega )}, \end{aligned} \end{aligned}$$

which is valid for any $\phi \in {W}^{1,\infty }(\Omega )$, where we have used (2.5). $\square$

In what follows, we always take this extension when we test the normal trace against a function in ${W}^{1,\infty }(\Omega )$. Note in particular that

2.11 $$\begin{aligned} \langle {{\varvec{F}}}\cdot \nu , \,\mathbbm {1}_{\Omega }\rangle _{\partial U} = -(\operatorname {div}{{\varvec{F}}})(U) \quad \text {for any } U \Subset \Omega , \end{aligned}$$

since $\mathbbm {1}_{\Omega } \in {W}^{1,\infty }(\Omega )$ is a valid test function and $\overline{\nabla \mathbbm {1}_{\Omega } \cdot {{\varvec{F}}}} = 0$.

#### Lemma 2.10

Let ${{\varvec{F}}}\in \mathcal {D}\mathcal {M}^{\textrm{ext}}(\Omega )$ with $\operatorname {spt}(\left| {{{\varvec{F}}}}\right| ) \subset \Omega$. Then

$$\begin{aligned} \operatorname {div}{{\varvec{F}}}(\Omega ) =0. \end{aligned}$$

#### Proof

  We extend ${{\varvec{F}}}$ to a measure ${\tilde{{{\varvec{F}}}}}$ in ${\mathbb {R}}^{n}$ by setting $|{\tilde{{{\varvec{F}}}}}|(\mathbb {{\mathbb {R}}}^{n} \setminus \Omega )=0$. We claim that ${\tilde{{{\varvec{F}}}}} \in \mathcal{D}\mathcal{M}^{{{\,\textrm{ext}\,}}}({\mathbb {R}}^n)$ and that $\operatorname {div}{\tilde{{{\varvec{F}}}}}$ is the zero-extension of $\operatorname {div}{{\varvec{F}}}$ to ${\mathbb {R}}^n$. Indeed, for $\phi \in C^{\infty }_{\textrm{c}}({\mathbb {R}}^n)$, $\operatorname {spt}(\phi ) \cap \operatorname {spt}(|{{\varvec{F}}}|) \subset \Omega$ is compact, so that we can find $\chi \in C_{\textrm{c}}^{\infty }(\Omega )$ such that $\chi = 1$ in a neighborhood of $\operatorname {spt}(\phi ) \cap \operatorname {spt}(|{{\varvec{F}}}|)$. Then $\chi \phi \in C^{\infty }_{\textrm{c}}(\Omega )$ and

$$\begin{aligned} \int _{{\mathbb {R}}^n} \nabla \phi \cdot \, \textrm{d}{{\tilde{{{\varvec{F}}}}}} = \int _{\Omega } \nabla (\chi \phi ) \cdot \,\textrm{d}{{\varvec{F}}}= - \int _{\Omega } \chi \phi \,\textrm{d}(\operatorname {div}{{\varvec{F}}}) = - \int _{\Omega } \phi \,\textrm{d}(\operatorname {div}{{\varvec{F}}}) \end{aligned}$$

by our choice of $\chi$. Since $\phi$ was arbitrary, it follows that ${{\tilde{{{\varvec{F}}}}}} \in \mathcal{D}\mathcal{M}^{{{\,\textrm{ext}\,}}}(\Omega )$ with the claimed divergence.

Now, let $\chi _k \in C^{\infty }_{\textrm{c}}(\Omega )$ such that $\mathbbm {1}_{B_k(0)} \le \chi _k \le \mathbbm {1}_{B_{k+1}(0)}$ in ${\mathbb {R}}^n$ and $\Vert \nabla \chi _k \Vert _{L^{\infty }(\Omega )} \le 2$. By definition of the distributional derivative,

$$\begin{aligned} \int _{\Omega } \chi _k\, \textrm{d}(\operatorname {div}{{\varvec{F}}}) = \int _{{\mathbb {R}}^n} \chi _k \,\textrm{d}(\operatorname {div}{{\tilde{{{\varvec{F}}}}}}) = - \int _{{\mathbb {R}}^n} \nabla \chi _k \cdot \textrm{d}{{\tilde{{{\varvec{F}}}}}}. \end{aligned}$$

Since $\nabla \chi _k$ is uniformly bounded in $L^{\infty }$ and converges pointwise to zero as $k \rightarrow \infty$, the Dominated Convergence Theorem leads to

$$\begin{aligned} \lim _{k \rightarrow \infty } \int _{{\mathbb {R}}^n} \nabla \chi _k \cdot \textrm{d}\tilde{{\varvec{F}}}= 0. \end{aligned}$$

Similarly, since $\chi _k$ converges pointwise to $\mathbbm {1}_{{\mathbb {R}}^n}$ and $\operatorname {div}{{\varvec{F}}}$ is a finite measure, we have

$$\begin{aligned} \operatorname {div}{{\varvec{F}}}(\Omega ) = \lim _{k \rightarrow \infty } \int _{\Omega } \chi _k \,\textrm{d}(\operatorname {div}{{\varvec{F}}}) = \lim _{k \rightarrow \infty } \int _{{\mathbb {R}}^n} \nabla \chi _k \cdot \textrm{d}{{\tilde{{{\varvec{F}}}}}} = 0, \end{aligned}$$

as required. $$\square $$

#### Corollary 2.11

Let ${{\varvec{F}}}\in \mathcal{D}\mathcal{M}^{{{\,\textrm{ext}\,}}}(\Omega )$. Then, for any open set $U \Subset \Omega$,

$$\begin{aligned} \langle {{\varvec{F}}}\cdot \nu , \,\phi \rangle _{\partial {{\overline{U}}}} = \int _{\Omega \setminus {{\overline{U}}}} \textrm{d}\overline{\nabla \phi \cdot {{\varvec{F}}}} + \int _{\Omega \setminus {{\overline{U}}}} \phi \,\textrm{d}(\operatorname {div}{{\varvec{F}}}) \quad \,\, \text{ for } \text{ any } \phi \in {W}_{\text {c}}^{1,\infty }(\Omega ). \end{aligned}$$

#### Proof

  By Lemma 2.10 and the product rule, for $\phi \in {W}^{1,\infty }_{\textrm{c}}(\Omega )$, we see that $\phi {{\varvec{F}}}\in \mathcal{D}\mathcal{M}^{{{\,\textrm{ext}\,}}}(\Omega )$ is compactly supported, so that

$$\begin{aligned} 0 = \int _{\Omega } \textrm{d}(\operatorname {div}(\phi {{\varvec{F}}})) = \int _{\Omega } \textrm{d}\overline{\nabla \phi \cdot {{\varvec{F}}}} + \int _{\Omega } \phi \,\textrm{d}(\operatorname {div}{{\varvec{F}}}). \end{aligned}$$

By splitting the latter integrals to integrate over ${{\overline{U}}}$ and $\Omega {\setminus } {{\overline{U}}}$ respectively, we deduce

$$\begin{aligned} 0 = - \langle {{\varvec{F}}}\cdot \nu , \,\phi \rangle _{\partial {{\overline{U}}}} + \int _{\Omega \setminus {{\overline{U}}}} \textrm{d}\overline{\nabla \phi \cdot {{\varvec{F}}}} + \int _{\Omega \setminus {{\overline{U}}}} \phi \,\textrm{d}(\operatorname {div}{{\varvec{F}}}), \end{aligned}$$

from which the result follows. $\square$

#### Remark 2.12

 Using Corollary 2.11, we can interpret $-\langle {{\varvec{F}}}\cdot \nu , \,\cdot \,\rangle _{\partial {{\overline{U}}}}$ as the *exterior normal trace* of ${{\varvec{F}}}$ on $\partial U$. Since

2.12 $$\begin{aligned} \langle {{\varvec{F}}}\cdot \nu , \,\phi \rangle _{\partial U} - \langle {{\varvec{F}}}\cdot \nu , \,\phi \rangle _{\partial {{\overline{U}}}} = \int _{\partial U} \textrm{d}\overline{\nabla \phi \cdot {{\varvec{F}}}} + \int _{\partial U} \phi \, \textrm{d}(\operatorname {div}{{\varvec{F}}}), \end{aligned}$$

the interior and exterior traces agree (up to a sign), provided that $|{{\varvec{F}}}|(\partial U) = |\operatorname {div}{{\varvec{F}}}|(\partial U) = 0$, by using the fact that $|\overline{\nabla \phi \cdot {{\varvec{F}}}}|\ll |{{\varvec{F}}}|$ by (2.5). In particular, this holds for $\partial U^{\varepsilon }$ for all but countably many $\varepsilon >0$. In general, this need not hold, for which we say that there is a *jump* across the boundary $\partial U$ if the two traces do not coincide. We emphasize that this does not occur if $\left| {\operatorname {div}{{\varvec{F}}}}\right| \ll {\mathcal {L}}^{n}$ and $\left| {{{\varvec{F}}}}\right| \ll {\mathcal {L}}^{n}$, provided that ${\mathcal {L}}^n(\partial U) = 0$, so it is necessary to consider measure-valued fields to model such phenomena.

#### Example 2.13

 We now show, by means of an example, that the jump in (2.12) can occur due to the concentration of field ${{\varvec{F}}}$ itself. For this, consider $\Omega = {\mathbb {R}}^2$ and
where $e_1 = (1,0)$. Since ${{\varvec{F}}}$ is a Radon measure on ${\mathbb {R}}^2$ and

$$\begin{aligned} \int _{{\mathbb {R}}^2} \nabla \phi \cdot \textrm{d}{{\varvec{F}}}= \int _{{\mathbb {R}}} \partial _{x_1}\phi (x_1,0) \,\textrm{d}x_1 = 0 \quad \text{ for } \text{ any } \phi \in C^{1}_{\textrm{c}}({\mathbb {R}}^2), \end{aligned}$$

it follows that ${{\varvec{F}}}\in \mathcal{D}\mathcal{M}^{{{\,\textrm{ext}\,}}}_{{\textrm{loc}}}(\Omega )$. Then, considering $Q = (0,1)^2 \Subset {\mathbb {R}}^2$, we have

$$\begin{aligned} \langle {{\varvec{F}}}\cdot \nu , \,\phi \rangle _{\partial Q} = 0 \quad \text{ for } \phi \in C^{1}_{\textrm{c}}({\mathbb {R}}^2), \end{aligned}$$

since ${\textrm{supp}}\,({{\varvec{F}}}) \cap Q = \varnothing .$ On the other hand, we have

$$\begin{aligned} \langle {{\varvec{F}}}\cdot \nu , \,\phi \rangle _{\partial {{\overline{Q}}}} = \int _0^1 \partial _{x_1} \phi (x_1,0) \,\textrm{d}x_1 = \phi (1,0) - \phi (0,0). \end{aligned}$$

This implies that the normal traces on *Q* and ${{\overline{Q}}}$ are represented by measures such that

$$\begin{aligned} ({{\varvec{F}}}\cdot \nu )_{\partial {{\overline{Q}}}} = \delta _{(1,0)}-\delta _{(0,0)} \ne 0 = ({{\varvec{F}}}\cdot \nu )_{\partial Q}, \end{aligned}$$

exhibiting a jump across the boundary $\partial Q$, despite the fact that $\operatorname {div}{{\varvec{F}}}= 0$ in ${\mathbb {R}}^2$. This is in contrast to the $\mathcal{D}\mathcal{M}^p$ setting for which, for sufficiently regular domains (i.e., when ${\mathcal {L}}^n(\partial U) = 0$), there is a jump across the boundary if and only if $|\operatorname {div}{{\varvec{F}}}|(\partial U) \ne 0$.

We can also infer from Lemma 2.10 that the normal trace is supported on $\partial E$. We state this result by using the language of distributions.

#### Lemma 2.14

Let ${{\varvec{F}}}\in \mathcal {D}\mathcal {M}^{\textrm{ext}}(\Omega )$, and let $E \Subset \Omega$ a Borel set. Then the normal trace $\langle {{\varvec{F}}}\cdot \nu , \,\phi \rangle _{\partial E}$ is a distribution of order 1 supported on $\partial E$.

#### Proof

  For $\phi \in C^1_{\textrm{c}}(\Omega )$, we can estimate

$$\begin{aligned} \begin{aligned} \left|\langle {{\varvec{F}}}\cdot \nu , \,\phi \rangle _{\partial E}\right|&\le \int _{\Omega } |\phi |\,\textrm{d}|\operatorname {div}{{\varvec{F}}}|+ \int _{\Omega } |\nabla \phi |\, \textrm{d}|{{\varvec{F}}}|\\&\le \Vert \phi \Vert _{L^{\infty }(\Omega )} |\operatorname {div}{{\varvec{F}}}|(\Omega ) + \Vert \nabla \phi \Vert _{L^{\infty }(\Omega )} |{{\varvec{F}}}|(\Omega ), \end{aligned} \end{aligned}$$

from which it follows that the normal trace is a distribution of order 1 in $\Omega$.

For the second part, let $\phi \in C^1_{\textrm{c}}(\Omega )$ be supported in $\Omega \setminus \partial E$. Since $\partial E$ is compact, $\textrm{dist}(\operatorname {spt}(\phi ),\partial E)>0$ so that $V:= \operatorname {spt}(\phi ) \cap E \Subset E$. Then, by the product rule, we have

2.13 $$\begin{aligned} \langle {{\varvec{F}}}\cdot \nu , \,\phi \rangle _{\partial E} = -\operatorname {div}(\phi {{\varvec{F}}})(E) = -\operatorname {div}(\phi {{\varvec{F}}})(V), \end{aligned}$$

since $\phi$ vanishes in the relatively open set $E \setminus V$. Now, let $\chi \in C^1_{\textrm{c}}(\Omega )$ be a cutoff such that $\chi \equiv 1$ on *V* and vanishes on $\Omega \setminus {\overline{E}}$. Then $\chi \phi {{\varvec{F}}}\in \mathcal{D}\mathcal{M}^{{{\,\textrm{ext}\,}}}(\Omega )$ is compactly supported, agrees with $\phi {{\varvec{F}}}$ in a neighborhood of *E*, and vanishes outside *V*. Applying Lemma 2.10 leads to

$$\begin{aligned} \operatorname {div}(\phi {{\varvec{F}}})(V) = \operatorname {div}(\chi \phi {{\varvec{F}}})(V) = \operatorname {div}(\chi \phi {{\varvec{F}}})(\Omega ) = 0. \end{aligned}$$

We combine this with (2.13) to conclude the result. $\square$

We can further refine Lemma 2.14 to allow for the testing of functions that vanish merely on $\partial E$. The following is due to [64] for the case of open sets; we record the proof for completeness and observe that the result applies to more general cases.

#### Theorem 2.15

Let ${{\varvec{F}}}\in \mathcal{D}\mathcal{M}^{{{\,\textrm{ext}\,}}}(\Omega )$, and let $E \Subset \Omega$ be either open or closed, or Borel satisfying $|{{\varvec{F}}}|(\partial E) = 0$. Then, if $\phi \in {W}^{1,\infty }(\Omega )$ vanishes on $\partial E$,

$$\begin{aligned} \langle {{\varvec{F}}}\cdot \nu , \,\phi \rangle _{\partial E} = 0. \end{aligned}$$

#### Proof

We first consider the case when $\operatorname {spt}(\phi ) \cap \partial E = \varnothing$; in this case, the result follows by arguing exactly as in Lemma 2.14. For $\phi \in {W}^{1,\infty }(\Omega )$, we see that (2.13) remains true by Corollary 2.9, so the identical argument goes through.

For general $\phi \in {W}^{1,\infty }(\Omega )$ vanishing on $\partial E$, we reduce to the first case via an approximation argument. For $\delta >0$, define

2.14 $$\begin{aligned} d_{\delta }(x) = {\left\{ \begin{array}{ll} 0 &  \text { if } 0 \le \textrm{dist}(x,\partial E)< \delta ,\\ \frac{1}{\delta }(\textrm{dist}(x,\partial E) - \delta ) &  \text { if } \delta \le \textrm{dist}(x,\partial E) < 2\delta ,\\ 1 &  \text { if } \textrm{dist}(x,\partial E) \ge 2\delta . \end{array}\right. } \end{aligned}$$

Then $d_{\delta }$ is $\frac{1}{\delta }$-Lipschitz and vanishes in a neighborhood of $\partial E$. Since $d_{\delta } \phi$ is supported away from $\partial E$, we know that

2.15 $$\begin{aligned} 0 = \langle {{\varvec{F}}}\cdot \nu , \,d_{\delta }\phi \rangle _{\partial E} = -\int _{E} d_{\delta }\phi \,\textrm{d}(\operatorname {div}{{\varvec{F}}}) - \int _{E} \textrm{d}\overline{\nabla (d_{\delta }\phi )\cdot {{\varvec{F}}}} \end{aligned}$$

by above. We now argue that we can pass to the limit as $\delta \rightarrow 0$.

This can be seen as follows: Since $\phi$ vanishes on $\partial E$, by the Lipschitz property, we have

$$\begin{aligned} \sup _{B_{2\delta }(\partial E)} |\phi (x)|\le 2\delta \Vert \nabla \phi \Vert _{L^{\infty }(\Omega )}, \end{aligned}$$

where $B_{2\delta }(\partial E) = \{ x \in {\mathbb {R}}^n: \textrm{dist}(x,\partial E) < 2\delta \}$. By the product rule (namely, using (2.4)), we see that, for any $\psi \in C_{\textrm{c}}(\Omega )$,

$$\begin{aligned} \begin{aligned} \int _{\Omega } \psi \,\textrm{d}\overline{\nabla (d_{\delta }\phi )\cdot {{\varvec{F}}}}&= \lim _{\varepsilon \rightarrow 0} \int _{\Omega } \psi \nabla (d_{\delta }\phi )*\rho _{\varepsilon } \cdot \textrm{d}{{\varvec{F}}}\\&= \lim _{\varepsilon \rightarrow 0} \int _{\Omega } \psi (d_{\delta }\nabla \phi + \phi \nabla d_{\delta }) *\rho _{\varepsilon } \cdot \textrm{d}{{\varvec{F}}}\\&= \int _{\Omega } \psi \,d_{\delta } \,\textrm{d}\overline{\nabla \phi \cdot {{\varvec{F}}}} + \int _{\Omega } \psi \,\phi \,\textrm{d}\overline{\nabla d_{\delta } \cdot {{\varvec{F}}}}, \end{aligned} \end{aligned}$$

which implies

$$\begin{aligned} \overline{\nabla (d_{\delta }\phi )\cdot {{\varvec{F}}}} = d_{\delta }\,\overline{\nabla \phi \cdot {{\varvec{F}}}} + \phi \,\overline{\nabla d_{\delta } \cdot {{\varvec{F}}}} \quad \text{ as } \text{ measures } \text{ in } \Omega . \end{aligned}$$

Moreover, since $\operatorname {spt}(\nabla d_{\delta })\Subset A_{\delta } := \{ \delta \le \textrm{dist}(x,\partial E) \le 2\delta \} \subset B_{2\delta }(\partial E)$, $\overline{\nabla d_{\delta } \cdot {{\varvec{F}}}}$ is also supported in $A_{\delta }$. Hence, using (2.5), we can estimate

2.16 $$\begin{aligned} \begin{aligned} \left|\int _{E} \phi \,\textrm{d}\overline{\nabla d_{\delta } \cdot {{\varvec{F}}}}\right|&\le \sup _{B_{2\delta }(\partial E)}|\phi |\Vert \nabla d_{\delta } \Vert _{L^\infty (\Omega )}|{{\varvec{F}}}|(A_{\delta })\\&\le 2 \Vert \nabla \phi \Vert _{L^\infty (\Omega )} |{{\varvec{F}}}|(A_{\delta }), \end{aligned} \end{aligned}$$

which vanishes as $\delta \rightarrow 0$ since $\displaystyle \limsup _{\delta \rightarrow 0} A_{\delta } = \varnothing$. Thus, passing to the limit in (2.15) and noting that $d_{\delta } \rightarrow \mathbbm {1}_{\Omega \setminus \partial E}$ pointwise,

2.17 $$\begin{aligned} \begin{aligned} 0&= \lim _{\delta \rightarrow 0} \langle {{\varvec{F}}}\cdot \nu , \,d_{\delta }\phi \rangle _{\partial E}\\&= \lim _{\delta \rightarrow 0} \int _E d_{\delta }\phi \, \textrm{d}(\operatorname {div}{{\varvec{F}}}) + \lim _{\delta \rightarrow 0} \int _E d_{\delta } \,\textrm{d}\overline{\nabla \phi \cdot {{\varvec{F}}}} \\&= \int _{E \setminus \partial E} \phi \, \textrm{d}(\operatorname {div}{{\varvec{F}}}) + \int _{E \setminus \partial E} \textrm{d}\overline{\nabla \phi \cdot {{\varvec{F}}}}, \end{aligned} \end{aligned}$$

by (2.16) and the Dominated Convergence Theorem.

Moreover, since $\phi$ vanishes in $\partial E$, it follows that

$$\begin{aligned} \int _{E \setminus \partial E} \phi \,\textrm{d}(\operatorname {div}{{\varvec{F}}}) = \int _E \phi \,\textrm{d}(\operatorname {div}{{\varvec{F}}}). \end{aligned}$$

Now, if *E* is open, then $E \setminus \partial E = 0$, and (2.17) implies that $\langle {{\varvec{F}}}\cdot \nu , \,\phi \rangle _{\partial E} = 0$. If *E* is closed, the same argument applies to $\Omega \setminus E$ so that, by Corollary 2.11,

$$\begin{aligned} \langle {{\varvec{F}}}\cdot \nu \rangle _{\partial E} = -\int _{\Omega \setminus E} \phi \,\textrm{d}(\operatorname {div}{{\varvec{F}}}) - \int _{\Omega \setminus E} \textrm{d}\overline{\nabla \phi \cdot {{\varvec{F}}}} = 0. \end{aligned}$$

Finally, if *E* is merely Borel with $|{{\varvec{F}}}|(\partial E) = 0$, then, since $\overline{\nabla \phi \cdot {{\varvec{F}}}} \ll |{{\varvec{F}}}|$ by (2.5), it follows that

$$\begin{aligned} \int _{E \cap \partial E} \textrm{d}\overline{\nabla \phi \cdot {{\varvec{F}}}} = 0. \end{aligned}$$

Combining this with (2.17), we conclude the proof. $\square$

#### Remark 2.16

  We expect that the conclusion of Theorem 2.15 holds for any Borel set $E \Subset \Omega$. However, we were unable to establish this without a further topological or measure-theoretic condition to ensure that $|\overline{\nabla \phi \cdot {{\varvec{F}}}} |(E \cap \partial E) = 0$. Note that, for $\mathcal{D}\mathcal{M}^p(\Omega )$–fields ${{\varvec{F}}}$, under the mild regularity condition that ${\mathcal {L}}^n(\partial E)= 0$, the condition that $|{{\varvec{F}}}|(\partial E) = \int _{\partial E} |{{\varvec{F}}}|\,\textrm{d}{\mathcal {L}}^n= 0$ is always satisfied.

#### Corollary 2.17

Let ${{\varvec{F}}}\in \mathcal{D}\mathcal{M}^{{{\,\textrm{ext}\,}}}(\Omega )$, and let $U \Subset \Omega$ be open. Then there is a linear functional $N_U \in \textrm{Lip}_{\textrm{b}}(\partial U)^{*}$ satisfying

$$\begin{aligned} \langle {{\varvec{F}}}\cdot \nu , \,\phi \rangle _{\partial U} = N_U(\phi |_{\partial U}) \quad \text {for any } \phi \in \textrm{Lip}_{\textrm{b}}(\Omega ). \end{aligned}$$

#### Proof

Given $\phi _0 \in \textrm{Lip}_{\textrm{b}}(\partial U)$, let $\phi \in \textrm{Lip}_{\textrm{b}}(\Omega )$ be an extension of $\phi _0$ to $\Omega$; this is possible by [2, Proposition 2.12] and multiplying the obtained extension by the Lipschitz cutoff $\chi (x) = \max \{0,1-\textrm{dist}(x,\partial U)\}$ to ensure that it is bounded. We then define

$$\begin{aligned} N_U(\phi _0) := \langle {{\varvec{F}}}\cdot \nu , \phi \rangle _U. \end{aligned}$$

Note that this extension can be chosen to satisfy that $\Vert \phi \Vert _{\textrm{Lip}_{\textrm{b}}(\Omega )} \le C\Vert \phi \Vert _{\textrm{Lip}_{\textrm{b}}(\partial U)},$ so it follows from the boundedness of $\langle {{\varvec{F}}}\cdot \nu , \,\cdot \,\rangle _{\partial U}$ that $N_U$ is bounded. To show that it is well-defined, observe that, if $\phi _1, \phi _2 \in \textrm{Lip}_{\textrm{b}}(\Omega ) \subset {W}^{1,\infty }(\Omega )$ are two such extensions, then $\phi _1-\phi _2$ vanishes on $\partial U$ so that

$$\begin{aligned} \langle {{\varvec{F}}}\cdot \nu , \,\phi _1 \rangle _U - \langle {{\varvec{F}}}\cdot \nu , \,\phi _2 \rangle _U = \langle {{\varvec{F}}}\cdot \nu , \,\phi _1 - \phi _2 \rangle = 0, \end{aligned}$$

by Theorem 2.15. Therefore, $N_U$ is well-defined, independent of the choice of extension. $\square$

## Representation and Limit Formula for the Normal Trace via Disintegration

In this section, we show the normal trace admits a measure representation on *almost every* open set and derive a limit formula in the general case. A key tool in our analysis is the disintegration of measures, which we apply in the following form:

### Theorem 3.1

Let $\mu$ be a finite Radon measure on an open set $U \subset {\mathbb {R}}^n$ such that $U \ne \varnothing , {\mathbb {R}}^n$, and let $d=\textrm{dist}(\,\cdot ,\partial U)$ be the distance function. Then there exist both a finite non-negative Radon measure $\tau$ on $(0, \infty )$ and a family of measures $\mu _t$ in *U* such that the mapping: $t \mapsto \mu _t$ is $\tau$-measurable. For $\tau$–*a.e.* $t \in (0,\infty )$, $\mu _t$ is supported on $d^{-1} (t)$ with $\left| {\mu _t}\right| (d^{-1}(t)) = 1$ and

$$\begin{aligned} \mu (A \cap U^{t_1}\setminus {\overline{U}}^{t_2}) = \int _{t_1}^{t_2} \int _{d^{-1} (t)} \chi _A(x) \,\textrm{d}\mu _t(x)\,\textrm{d}\tau (t), \end{aligned}$$

where the $\tau$-integral is understood to be over the open interval $(t_1,t_2)$. Furthermore, for any bounded Borel function $\phi$ on *U*,

$$\begin{aligned} \int _U \phi (x) \,\textrm{d}\mu (x) = \int _0^{\infty } \int _{d^{-1}(t)} \phi (x) \,\textrm{d}\mu _t(x)\,\textrm{d}\tau (t). \end{aligned}$$

We write

3.1 $$\begin{aligned} \mu = \tau \otimes _{\partial U^t} \mu _t \end{aligned}$$

as a shorthand for this decomposition.

### Proof

We define the function $\Phi :U \rightarrow (0, \infty ) \times U$ as $\Phi (x)=(d(x),x)$. Then the push-forward measure $\Phi _{\#}\mu$ is a measure on $(0, \infty ) \times U$. By the Disintegration Theorem presented in [2, Theorem 2.28] applied to $\Phi _{\#}\mu$, it follows that there exists a family of measures $\mu _t$ satisfying

$$\begin{aligned} \Phi _{\#}\mu = \tau \otimes \mu _t, \quad \left| {\Phi _{\#}\mu }\right| = \tau \otimes \left| {\mu _t}\right| , \end{aligned}$$

where $\tau$ is a measure on $(0, \infty )$ defined as

$$\begin{aligned} \tau = \pi _{\#} (\left| {\Phi _{\#}\mu }\right| )\quad \text { with }\pi :(0, \infty ) \times U \rightarrow (0, \infty ) \text{ and } \pi (t,x)=t, \end{aligned}$$

and $\mu _t$ is a family of measures on *U* with $\left| {\mu _t}\right| (U)=1$ for $\tau$–*a.e.* $t \in (0, \infty )$. For a Borel set $A \subset U \setminus {{\overline{U}}}^t$, since $\Phi$ is injective, $\Phi ^{-1}(\Phi (A)) = A$, so that

3.2 $$\begin{aligned} \mu (A) = \Phi _{\#}\mu (\Phi (A)) = \int _0^t \int _U \chi _{\Phi ^{-1}(A)}(t,x)\,\textrm{d}\mu _t(x)\,\textrm{d}\tau (t). \end{aligned}$$

We now claim that, for $\tau$–*a.e.* $t \in (0,\infty )$, $\mu _t$ is supported on $\partial U_t$. Indeed, for any $t_1 < t_2$, we have

$$\begin{aligned} \tau ((t_1,t_2))&= \pi _{\#} (\left| {\Phi _{\#}\mu }\right| ) ((t_1,t_2))=\left| {\Phi _{\#}\mu }\right| (\pi ^{-1}((t_1,t_2))) \\&= \left| {\Phi _{\#}\mu }\right| ((t_1,t_2)\times U)= \Phi _{\#}\left| {\mu }\right| ((t_1,t_2)\times U) \\&= \left| {\mu }\right| (\Phi ^{-1}((t_1,t_2) \times U))= \left| {\mu }\right| (U^{t_2}\setminus {\overline{U}}^{t_1}), \end{aligned}$$

and

$$\begin{aligned} \left| {\mu }\right| (U^{t_2}\setminus {\overline{U}}^{t_1})= \left| {\Phi _{\#}\mu }\right| \big ((t_1,t_2) \times (U^{t_2}\setminus {\overline{U}}^{t_1})\big )=\int _{t_1}^{t_2}\int _{U^{t_2} \setminus \overline{U^{t_1}}} \,\textrm{d}\left| {\mu _t}\right| \,\textrm{d}\tau (t). \end{aligned}$$

Combining the above, we infer that

$$\begin{aligned} 1 = \frac{|\mu |(U^{t_2} \setminus \overline{U^{t_1}})}{\tau ((t_1,t_2))} = \frac{1}{\tau ((t_1,t_2))} \int _{t_1}^{t_2} |\mu _t|(U \setminus \overline{U^{t_1}})\,\textrm{d}\tau (t). \end{aligned}$$

Let $t>0$ and $0< \varepsilon < \delta$. Then we can employ the above with $t_1 = t-\varepsilon$ and $t_2 = t+\varepsilon$, and apply that $U^{t+\varepsilon } \setminus \overline{U^{t-\varepsilon }}\subset U^{t+\delta } \setminus \overline{U^{t-\delta }}$ to obtain

$$\begin{aligned} \frac{1}{\tau ((t-\varepsilon ,t+\varepsilon ))} \int _{t-\varepsilon }^{t+\varepsilon } |\mu _s |(U^{t+\delta } \setminus \overline{U^{t-\delta }}) \,\textrm{d}\tau (s) \ge 1. \end{aligned}$$

Since the function

3.3 $$\begin{aligned} s \mapsto |\mu _s |(U^{t+\delta } \setminus \overline{U^{t-\delta }}) \end{aligned}$$

is $\tau$-measurable for $t \ge 0$ by [2, (2.19)], there is a $\tau$-null set ${\mathcal {N}} \subset [0,\infty )$ such that every $t \notin {\mathcal {N}}$ is a Lebesgue point for (3.3) for each $\delta = \frac{1}{k}$ with $k \in {\mathbb {N}}$. Then, for such *t*, we have

$$\begin{aligned} 1 \le \lim _{\varepsilon \rightarrow 0} \frac{1}{\tau ((t-\varepsilon ,t+\varepsilon ))} \int _{t-\varepsilon }^{t+\varepsilon } |\mu _s |(U^{s+\frac{1}{k}} \setminus \overline{U^{s-\frac{1}{k}}}) \,\textrm{d}\tau (s) = |\mu _t |(U^{t+\frac{1}{k}} \setminus \overline{U^{t-\frac{1}{k}}}). \end{aligned}$$

Since this holds for all *k*, we infer that

$$\begin{aligned} |\mu _t |(\partial U^t) = \lim _{k \rightarrow \infty } |\mu _t|(U^{t+\frac{1}{k}} \setminus \overline{U^{t-\frac{1}{k}}}) \ge 1. \end{aligned}$$

However, since $|\mu _t|(U)=1$, it follows that $\mu _t$ is supported on $\partial U^t$.

Since $\tau$-almost every $\mu _t$ is supported on $\partial U^t$, (3.2) simplifies to give

$$\begin{aligned} \mu (A) = \int _0^t \int _{\partial U^t} \chi _A(x)\,\textrm{d}\mu (x)\,\textrm{d}\tau (t), \end{aligned}$$

which is what we set out to prove. Moreover, an approximation argument implies (3.1). $\square$

### Lemma 3.2

Let $\mu$ be a Radon measure on an open set $U \subset {\mathbb {R}}^n$. Consider the decomposition

$$\begin{aligned} \mu = \tau \otimes _{\partial U^t} \mu _t \end{aligned}$$

from Theorem 3.1. Then, for $\tau$–*a.e.* $t \in [0,\infty )$,

$$\begin{aligned} D_{|\mu |}\mu (x) = D_{|\mu ^t|}\mu ^t(x) \quad |\mu ^t|\textit{--a.e.}\,\,\text{ on } \partial U^t. \end{aligned}$$

### Proof

  We know from the above proof that $\Phi _{\#}\mu = \tau \otimes \mu _t$ and $|\Phi _{\#}\mu |= \tau \otimes |\mu _t |$. Thus, if $\phi$ is a bounded Borel-measurable function on *U*, we have

$$\begin{aligned} \int _U \phi \,\textrm{d}\mu = \int _0^{\infty } \int _{\partial U^t} \phi \,\textrm{d}\mu _t\,\textrm{d}\tau = \int _0^{\infty } \int _{\partial U^t} \phi \, D_{|\mu _t|}\mu _t \,\textrm{d}|\mu _t|\,\textrm{d}\tau , \end{aligned}$$

and

$$\begin{aligned} \int _U \phi \,\textrm{d}\mu = \int _U \phi \,D_{|\mu |}\mu \,\textrm{d}|\mu |= \int _0^{\infty } \int _{\partial U^t} \phi \, D_{|\mu |}\mu \,\textrm{d}|\mu _t|\,\textrm{d}\tau . \end{aligned}$$

Now, replacing $\phi$ by $\mathbbm {1}_{U^{t_2} \setminus \overline{U^{t_1}}}\phi$ and then combining the above, we obtain

$$\begin{aligned} \int _{t_1}^{t_2} \int _{\partial U^t} \phi \, D_{|\mu _t|}\mu _t \,\textrm{d}|\mu _t|\,\textrm{d}\tau = \int _{t_1}^{t_2} \int _{\partial U^t} \phi \,D_{|\mu |}\mu \,\textrm{d}|\mu _t|\,\textrm{d}\tau . \end{aligned}$$

Let $\{\phi _j\}$ be a countable and uniformly dense subset of $C_{\textrm{c}}(U)$. Then, for $\tau$–*a.e.* $t \ge 0$,

$$\begin{aligned} \begin{aligned} \int _{\partial U^t} \phi _j\, D_{|\mu _t|}\mu _t \,\textrm{d}|\mu _t|&= \lim _{\varepsilon \rightarrow 0} \int _{t-\varepsilon }^{t+\varepsilon } \int _{\partial U^s} \phi _j \,D_{|\mu _s|}\mu _s \,\textrm{d}|\mu _s|\,\textrm{d}\tau \\&=\lim _{\varepsilon \rightarrow 0} \int _{t-\varepsilon }^{t+\varepsilon } \int _{\partial U^s} \phi _j\,D_{|\mu |}\mu \,\textrm{d}|\mu _s|\,\textrm{d}\tau \\&= \int _{\partial U^t} \phi _j\,D_{|\mu |}\mu \,\textrm{d}|\mu _t|. \end{aligned} \end{aligned}$$

By the density of $\{\phi _j\}$, it follows that, for any such *t*,

$$\begin{aligned} (D_{|\mu _t|}\mu _t) |\mu _t|= (D_{|\mu |}\mu ) |\mu _t|\quad \text{ as } \text{ measures } \text{ on } \partial U^t, \end{aligned}$$

from which the result follows. $\square$

Let $\tau$ be a non-negative Radon measure on ${\mathbb {R}}$. In the subsequent proof, we apply the Lebesgue-Besicovitch Differentiation Theorem to write

$$\begin{aligned} \tau = (D_{{\mathcal {L}}^1} \tau ) {\mathcal {L}}^1 + \tau _{\text {sing}}, \end{aligned}$$

where the Radon measure $\tau _{\text {sing}}$ satisfies
By Lemma 2.1, $D_{{\mathcal {L}}^1}\tau _{\textrm{sing}}(t) = 0$ for ${\mathcal {L}}^1$–*a.e.* $t>0$.

### Theorem 3.3

Let $F\in \mathcal {D}\mathcal {M}^{\textrm{ext}}(\Omega )$, and let $U \Subset \Omega$ be open. Then there is an ${\mathcal {L}}^1$–null set ${\mathcal {N}} \subset (0,\infty )$ such that, for any $\varepsilon \notin {\mathcal {N}}$, there exists a measure $({{\varvec{F}}}\cdot \nu )_{\partial U^{\varepsilon }}$ supported on $\partial U^{\varepsilon }$ such that

$$\begin{aligned} \langle {{\varvec{F}}}\cdot \nu , \,\phi \rangle _{\partial U^{\varepsilon }} = \int _{\partial U^{\varepsilon }} \phi \,\textrm{d}({{\varvec{F}}}\cdot \nu )_{\partial U^{\varepsilon }} \quad \text {for any } \phi \in {W}^{1,\infty }(\Omega ). \end{aligned}$$

Moreover, for every $\varepsilon _k \rightarrow 0$ with $\varepsilon _k \notin {\mathcal {N}}$, the normal trace of ${{\varvec{F}}}$ on $\partial U$ can be represented as the limit of trace measures:

$$\begin{aligned} \langle {{\varvec{F}}}\cdot \nu , \,\phi \rangle _{ \partial U} = \lim _{k \rightarrow \infty } \int _{\partial U^{\varepsilon _{k}}} \phi \,\textrm{d}({{\varvec{F}}}\cdot \nu )_{\partial U^{\varepsilon _{k}}} \quad \text {for any } \phi \in {W}^{1,\infty }(\Omega ). \end{aligned}$$

### Proof

  We divide the proof into three steps.

**1**. For each $0<t<s$, we define $\psi _{t,s}^U \in \textrm{Lip}_{\textrm{c}}(\Omega )$ by

3.4 $$\begin{aligned} \psi _{t,s}^U(x) = \left\{ \begin{array}{ll} s-t &  \text { if } x \in U^{s}, \\ d(x)-t &  \text { if } x \in U^t \setminus U^{s}, \\ 0 &  \text { if } x \not \in U^t. \end{array} \right. \end{aligned}$$

We first show that

3.5 $$\begin{aligned} \int _{U} \psi _{t,s}^U \, \textrm{d}(\operatorname {div}(\phi {{\varvec{F}}})) = -\int _{U^t \setminus \overline{U^{s}}} \phi \, \textrm{d}\overline{\nabla d \cdot {{\varvec{F}}}} \end{aligned}$$

for any $\phi \in {W}^{1,\infty }(\Omega )$ and all $0< t < s$ for which

3.6 $$\begin{aligned} |\overline{\nabla d \cdot {{\varvec{F}}}}|(\partial U^t) = |\overline{\nabla d \cdot {{\varvec{F}}}}|(\partial U^s) = 0 \end{aligned}$$

that holds for all but countably many *s* and *t*.

Since $\psi ^U_{t,s}$ is supported away from $\partial U$, by Lemma 2.14, we have

$$\begin{aligned} 0 = \langle {{\varvec{F}}}\cdot \nu , \,\psi _{t,s}^U \phi \rangle _{\partial U} = \operatorname {div}(\phi \psi _{t,s}^U {{\varvec{F}}})(U) = \langle \phi {{\varvec{F}}}\cdot \nu , \,\psi _{t,s}^U \rangle _{\partial U}. \end{aligned}$$

Now, using the product rule (Theorem 2.7), we have

$$\begin{aligned} 0= \langle \phi {{\varvec{F}}}\cdot \nu , \,\psi _{t,s}^U \rangle _{\partial U} = \int _{U} \psi _{t,s}^U \,\textrm{d}(\operatorname {div}(\phi {{\varvec{F}}})) + \int _{U} \textrm{d}\overline{ \nabla \psi _{t,s}^U \cdot (\phi {{\varvec{F}}}) }. \end{aligned}$$

By definition of the pairing measure,
where we have used (3.6) to justify the weak ${}^*$–limit in the last equality. Combining this with (3.7) yields (3.5), which can be written as

3.8 $$\begin{aligned} (s-t)\int _{U^{s}}\textrm{d}(\operatorname {div}(\phi {{\varvec{F}}})) + \int _{U^t \setminus {\overline{U}}^{s}}(d(x)-t) \, \textrm{d}(\operatorname {div}(\phi {{\varvec{F}}})) = -\int _{U^t \setminus \overline{U^{s}}} \phi \, \textrm{d}\overline{\nabla d \cdot {{\varvec{F}}}}, \end{aligned}$$

by definition of $\psi _{t,s}^U$.

**2**. We now apply the disintegration result (Theorem 3.1) with $\mu = \overline{\nabla d \cdot {{\varvec{F}}}}$ to write

$$\begin{aligned} \int _{U^t \setminus \overline{U^s}} \phi \,\textrm{d}\overline{\nabla d \cdot {{\varvec{F}}}} = \int _t^s \int _{\partial U^r} \phi \,\textrm{d}\mu _r\,\textrm{d}\tau (r). \end{aligned}$$

Now, given $\varepsilon , h>0$ with $h < \varepsilon$, we apply (3.8) with $s = \varepsilon +h$ and $t = \varepsilon -h$ to obtain

3.9 $$\begin{aligned} \begin{aligned}&- \int _{\varepsilon -h}^{\varepsilon +h} \int _{\partial U^t} \phi \,\text {d}\mu _t\,\text {d}\tau (t) \\  &= 2h \int _{U^{\varepsilon +h}} \text {d}(\operatorname {div}(\phi {{\varvec{F}}})) + \int _{U^{\varepsilon -h} \setminus \overline{U^{\varepsilon +h}}} (d(x) -\varepsilon +h)\,\text {d}(\operatorname {div}(\phi {{\varvec{F}}})). \end{aligned} \end{aligned}$$

This is valid for all but countably many $h>0$ depending on $\varepsilon$. We also impose that

3.10 $$\begin{aligned} |{{\varvec{F}}}|(\partial U^{\varepsilon }) = |\operatorname {div}{{\varvec{F}}}|(\partial U^{\varepsilon }) = 0, \end{aligned}$$

which holds for all but countably many $\varepsilon$.

We now divide both sides by 2*h* and study the limit as $h \rightarrow 0$. Let $h_k \searrow 0$ be any sequence such that (3.9) is valid with each $h_k$ in place of *h*. Since

$$\begin{aligned} |d(x) -\varepsilon + h_k|\le 2h_k\quad \,\, \text { on } U^{\varepsilon -h_k}\setminus {\overline{U}}^{\varepsilon +h_k} \end{aligned}$$

and $|\operatorname {div}(\phi {{\varvec{F}}})|(U^{\varepsilon -h_k}\setminus {\overline{U}}^{\varepsilon +h_k}) \rightarrow 0$ as $k \rightarrow \infty$ by (3.10), we have

$$\begin{aligned} \lim _{k \rightarrow \infty } \frac{1}{2h_k}\int _{U^{\varepsilon -h_k} \setminus \overline{U^{\varepsilon +h_k}}} (d(x) -\varepsilon +h_k)\,\textrm{d}(\operatorname {div}(\phi {{\varvec{F}}})) = 0. \end{aligned}$$

The Dominated Convergence Theorem gives

$$\begin{aligned} \lim _{k \rightarrow \infty } \int _{U^{\varepsilon +h_k}} \textrm{d}(\operatorname {div}(\phi {{\varvec{F}}})) = \int _{U^\varepsilon } \textrm{d}(\operatorname {div}(\phi {{\varvec{F}}})) = -\langle {{\varvec{F}}}\cdot \nu , \,\phi \rangle _{\partial U^{\varepsilon }}. \end{aligned}$$

Thus, we infer that

3.11 $$\begin{aligned} \langle {{\varvec{F}}}\cdot \nu , \,\phi \rangle _{\partial U^{\varepsilon }} = \lim _{k \rightarrow \infty } \frac{1}{2h_k} \int _{\varepsilon -h_k}^{\varepsilon +h_k} \int _{\partial U^t} \phi \,\textrm{d}\mu _t\,\textrm{d}\tau (t). \end{aligned}$$

**3**. To proceed, we decompose measure $\tau$ in two parts: the absolutely continuous part with respect to ${\mathcal {L}}^{1}$ and the singular part as

$$\begin{aligned} \tau = (D_{{\mathcal {L}}^{1}} \tau ) {\mathcal {L}}^{1} + \tau _{\text {sing}}, \quad D_{{\mathcal {L}}^{1}} \tau \in L^1_{\text {loc}}((0,\infty )). \end{aligned}$$

Hence, with $T:=D_{{\mathcal {L}}^{1}} \tau$, we have

3.12 $$\begin{aligned} \int _{\varepsilon - h_k}^{\varepsilon + h_k }\int _{\partial U^t}\phi \,\textrm{d}\mu _t \,\textrm{d}\tau (t) =\int _{\varepsilon - h_k}^{\varepsilon + h_k }\int _{\partial U^t}\phi \,\textrm{d}\mu _t \,T(t) \,\textrm{d}t +\int _{\varepsilon - h_k}^{\varepsilon + h_k }\int _{\partial U^t}\phi \,\textrm{d}\mu _t \,\textrm{d}\tau _{\text {sing}}(t). \end{aligned}$$

We can estimate the first integral on the right-hand side of (3.12) by using the Lebesgue Differentiation Theorem. Indeed, for ${\mathcal {L}}^1$*–a.e.* $\varepsilon$, we obtain

3.13 $$\begin{aligned} \lim _{k \rightarrow \infty } \frac{1}{2h_k} \int _{\varepsilon - h_k}^{\varepsilon + h_k } \Big (\int _{\partial U^t}\phi \,\textrm{d}\mu _t \Big ) T(t)\,\textrm{d}t =\int _{\partial U^\varepsilon }\phi \,T(\varepsilon )\,\textrm{d}\mu _{\varepsilon }, \end{aligned}$$

so that

3.14 $$\begin{aligned} ({{\varvec{F}}}\cdot \nu )_{\partial U^{\varepsilon }} = T(\varepsilon ) \mu _{\varepsilon }, \end{aligned}$$

from (3.11). Since $\left| {\mu _t}\right| (\partial U^{t})=1$ for $\tau _{\text {sing}}$*–a.e.*  *t*, we have

$$\begin{aligned} \Big |\int _{\partial U^{t}} \phi \,\textrm{d}\mu _{t} \Big |\le \left\Vert \phi \right\Vert _{L^{\infty }(\Omega )}\left| {\mu _{t}}\right| (\partial U^{t})=\left\Vert \phi \right\Vert _{L^{\infty }(\Omega )}. \end{aligned}$$

Using this and Lemma 2.1, we can estimate the second integral on the right-hand side of (3.12) as

3.15 $$\begin{aligned} \lim _{k \rightarrow \infty } \frac{1}{2h_k} \Big |\int _{\varepsilon - h_k}^{\varepsilon + h_k }\int _{\partial U^t}\phi \,\textrm{d}\mu _t \textrm{d}\tau _{\text {sing}}(t)\Big |&\le \left\Vert \phi \right\Vert _{L^{\infty }(\Omega )} \lim _{h_k \rightarrow 0} \frac{\tau _{\text {sing}} (\varepsilon -h_k, \varepsilon + h_k)}{{\mathcal {L}}^{1} (\varepsilon -h_k, \varepsilon + h_k)} \nonumber \\&= \left\Vert \phi \right\Vert _{L^{\infty }(\Omega )} D_{{\mathcal {L}}^1} \tau _{\text {sing}}(\varepsilon )=0 \end{aligned}$$

for ${\mathcal {L}}^{1}$–*a.e.* $\varepsilon >0$. Thus, defining ${\mathcal {N}} \subset (0,\infty )$ to be the set of points where either (3.10), (3.13), or (3.15) does not hold, which is ${\mathcal {L}}^1$–null, and from (3.14),

$$\begin{aligned} \langle {{\varvec{F}}}\cdot \nu , \,\phi \rangle _{\partial U^{\varepsilon }} = \int _{\partial U^{\varepsilon }} \phi \, \textrm{d}({{\varvec{F}}}\cdot \nu )_{\partial U^{\varepsilon }} \quad \text {for any }\varepsilon \notin {\mathcal {N}}. \end{aligned}$$

Finally, taking any $\varepsilon _k \rightarrow 0$ with $\varepsilon _k \notin {\mathcal {N}}$ for all *k*, we have

$$\begin{aligned} \langle {{\varvec{F}}}\cdot \nu , \,\phi \rangle _{\partial U} = \lim _{k \rightarrow \infty } \langle {{\varvec{F}}}\cdot \nu , \phi \rangle _{\partial U^{\varepsilon _k}} = \lim _{k \rightarrow \infty } \int _{\partial U^{\varepsilon _{k}}} \phi \,\textrm{d}({{\varvec{F}}}\cdot \nu )_{\partial U^{\varepsilon _{k}}} \end{aligned}$$

as required. $$\square $$

### Remark 3.4

 Similar results were previously obtained by Frid [40] for domains with Lipschitz deformable boundaries.

Later in Sect. 7.1, we will use a similar decomposition to $\overline{\nabla d \cdot {{\varvec{F}}}}$ from the above proof applied to a non-negative measure, which we record here.

### Lemma 3.5

Let $\mu$ be a non-negative Radon measure on an open set $U \subset {\mathbb {R}}^n$. Then there exists a decomposition
where $D_{{\mathcal {L}}^1}\tau _{\textrm{sing}} = 0$ $\,{\mathcal {L}}^1$–*a.e.*  on $[0,\infty )$, and we have used notation (3.1) from Theorem 3.1.

### Proof

  We apply Theorem 3.1 to $\mu$ to decompose

$$\begin{aligned} \mu = \tau \otimes _{\partial U^t} {{\tilde{\mu }}}_t, \end{aligned}$$

and then use the Lebesgue-Besicovitch Theorem to decompose
where ${\mathcal {L}}^1$ and $\tau _{\textrm{sing}}$ are mutually singular, and $T = D_{{\mathcal {L}}^1}\tau \in L^1((0,\infty ))$. Taking the precise representative of *T*, we set $\mu _t = T(t){\tilde{\mu }}_t$, which is defined ${\mathcal {L}}^1$–*a.e.*  on $(0,\infty )$. This gives the claimed decomposition by noting that the last part follows from Lemma 2.1. $\square$

## Properties of the Disintegration

In Sect. 3, we have established Theorem 3.3 by considering a suitable disintegration of $\overline{\nabla d \cdot {{\varvec{F}}}}$ along $\{\partial U^{\varepsilon }\}_{\varepsilon >0}$ and showing that the obtained measures $({{\varvec{F}}}\cdot \nu )_{\partial U^{\varepsilon }}$ coincided with the normal trace. We now investigate this decomposition more closely and show that measure $\overline{\nabla d \cdot {{\varvec{F}}}}$ can actually be recovered from these traces.

Informally, if we take $d(x) = x_i$, which corresponds to taking *U* to be a half-plane $\{ x \in {\mathbb {R}}^n: x_i \ge 0\}$ for some $1 \le i \le n$, then it follows from $\nabla d = e_i$ that $\overline{\nabla d \cdot {{\varvec{F}}}} = F_i$ for each $1 \le i \le n$, where ${{\varvec{F}}}= (F_1,F_2,\cdots ,F_n)$. Thus, such a result allows us to recover the underlying field ${{\varvec{F}}}$ from the associated normal traces over sets $\{\partial U^{\varepsilon }\}_{\varepsilon >0}$; while Theorem 4.1 cannot directly be applied to the half-space that is unbounded, we adapt this later in Lemma 8.3. This observation serves as a fundamental starting point for the theory of Cauchy fluxes in the extended setting, which will be developed from Sect. 6 onwards.

### Theorem 4.1

Let ${{\varvec{F}}}\in \mathcal {D}\mathcal {M}^{\textrm{ext}}(\Omega ),$ $U \Subset \Omega$, and let $d(x) = \textrm{dist}(x,\partial U)$. Then

4.1 $$\begin{aligned} \int _0^{\infty } \langle {{\varvec{F}}}\cdot \nu , \,\phi \rangle _{\partial U^{t}} \,\textrm{d}t = \int _{U } \phi \,\textrm{d}\overline{\nabla d \cdot {{\varvec{F}}}} \quad \text{ for } \text{ any } \phi \in {W}^{1,\infty }(\Omega ), \end{aligned}$$

where $\overline{\nabla d \cdot {{\varvec{F}}}}$ is given as in Theorem 2.7. Moreover, (4.1) extends to any bounded Borel function $\phi$ on $\Omega$, understanding that $t \mapsto \langle {{\varvec{F}}}\cdot \nu , \,\phi \rangle _{\partial U^t}$ is only defined for ${\mathcal {L}}^1$–*a.e.* $t>0$.

Equivalently, consider the disintegration given by Theorem 3.1, which allows us to write

4.2 $$\begin{aligned} \int _U \phi \,\textrm{d}\overline{\nabla d \cdot {{\varvec{F}}}} = \int _0^{\infty } \int _{d^{-1}(t)} \phi (x) \,\textrm{d}\mu _t(x)\,\textrm{d}\tau (t) \end{aligned}$$

for any bounded Borel function $\phi$ on *U*. Now, in Theorem 3.3, we have seen that

4.3 $$\begin{aligned} \langle {{\varvec{F}}}\cdot \nu , \cdot \rangle _{\partial U^t} =( {{\varvec{F}}}\cdot \nu )_{\partial U^\varepsilon }= D_{{\mathcal {L}}^1} \tau (t)\, \mu _t \quad \text {for }{\mathcal {L}}^1\textit{--a.e.}\,\, t>0, \end{aligned}$$

so Theorem 4.1 is equivalent to the assertion that $\tau _{\textrm{sing}} = 0.$ To prove this, we use the following elementary lemma.

### Lemma 4.2

Let $\tau$ and $\sigma$ be finite Radon measures on $(0,\infty )$ such that

$$\begin{aligned} \tau ' = \sigma \quad \text { in }{\mathcal {D}}'((0,\infty )), \end{aligned}$$

that is,

$$\begin{aligned} -\int _0^{\infty } \psi '(t) \,\textrm{d}\tau (t) = \int _0^{\infty } \psi (t) \,\textrm{d}\sigma (t) \quad \text {for any } \psi \in C^1_{\textrm{c}}((0,\infty )). \end{aligned}$$

Then $\tau \ll {\mathcal {L}}^1$ and there exists $T \in BV((0,\infty ))$ such that 

### Proof

  Define $v(x) = \sigma ((0,x))$ for $x>0$, which lies in $BV((0,\infty ))$. Then *v* satisfies $v' = \sigma$ and is bounded pointwise by $|\sigma |((0,\infty ))$. Define the measure: ${{\tilde{\tau }}} = \tau - v {\mathcal {L}}^1$. Since ${{\tilde{\tau }}}' = 0$ in ${\mathcal {D}}'((0,\infty ))$, we claim ${{\tilde{\tau }}}$ is a constant multiple of the Lebesgue measure. Indeed, mollifying ${{\tilde{\tau }}}$, we obtain a smooth function ${{\tilde{\tau }}}_{\delta }$ that satisfies ${{\tilde{\tau }}}_{\delta }' \equiv 0$ in $(0,\infty )$. Thus, for each $\delta >0$, there exists a constant $c_{\delta } \in {\mathbb {R}}$ such that ${{\tilde{\tau }}}_{\delta }(x) = c_{\delta }$ for all $x>\delta$. Since  as $\delta \rightarrow 0$ locally in $(0,\infty )$, we know that $c_{\delta } \rightarrow c$ for some $c \in {\mathbb {R}}$. Hence, $\tau = (v + c) {\mathcal {L}}^1$, which implies that $\tau$ is represented by a *BV* function. $\square$

### Proof of Theorem 4.1

We divide the proof into four steps.

**1**. Let $\psi \in C^1_{\textrm{c}}((0,\infty ))$ and $g \in C^1_{\textrm{c}}(\Omega )$, and set $\phi (x) = \psi (d(x)) g(x)$. Then $\phi \in \textrm{Lip}_{\textrm{c}}(U)$, and the mollification $\phi _{\delta }$ is defined and vanishes on $\partial U$ for $\delta >0$ sufficiently small. By Lemma 2.10, we have

$$\begin{aligned} \int _U \nabla \phi \cdot {{\varvec{F}}}_{\delta } \,\textrm{d}x = \int _U \nabla \phi _{\delta } \cdot \textrm{d}{{\varvec{F}}}= - \int _U \phi _{\delta }\, \textrm{d}(\operatorname {div}{{\varvec{F}}}) = - \int _U \phi \operatorname {div}{{\varvec{F}}}_{\delta } \,\textrm{d}x. \end{aligned}$$

Expanding $$\nabla \phi (x) = \psi '(d(x)) g(x)\nabla d(x) + \psi (d(x))\nabla g(x)$$ gives

$$\begin{aligned} -\int _U \psi '(d)\, g\, \nabla d \cdot {{\varvec{F}}}_{\delta } \,\textrm{d}x = \int _U \big (\phi \, (\operatorname {div}{{\varvec{F}}})_{\delta } + \psi (d) \nabla g \cdot {{\varvec{F}}}_{\delta }\big ) \,\textrm{d}x. \end{aligned}$$

Sending $\delta \rightarrow 0$ and using Theorem 2.7, we obtain

4.4 $$\begin{aligned} -\int _U \psi '(d) \, g \,\textrm{d}\overline{\nabla d \cdot {{\varvec{F}}}} = \int _U \phi \, \textrm{d}(\operatorname {div}{{\varvec{F}}}) + \int _U \psi (d)\, \nabla g \cdot \textrm{d}{{\varvec{F}}}. \end{aligned}$$

**2**. Now set $\lambda = g \, \operatorname {div}{{\varvec{F}}}+ \nabla g \cdot {{\varvec{F}}}$ and use Proposition 3.1 to write

$$\begin{aligned} \overline{\nabla d \cdot {{\varvec{F}}}}= \tau \otimes _{\partial U^t} \mu _t, \quad \lambda = \sigma \otimes _{\partial U^t} \lambda _t. \end{aligned}$$

Using this, we can rewrite (4.4) as

$$\begin{aligned} -\int _0^{\infty } \psi '(t)\int _{\partial U^t} g(x) \,\textrm{d}\mu _t(x) \,\textrm{d}\tau (t) = \int _0^{\infty } \psi (t) \int _{\partial U^t} \,\textrm{d}\lambda _t(x) \,\textrm{d}\sigma (t). \end{aligned}$$

Hence, as distributions on (0, 1),  we have shown that

$$\begin{aligned} \frac{\textrm{d}}{\textrm{d}t} \Big (\int _{\partial U^t} g(x) \,\textrm{d}\mu _t(x) \,\tau \Big ) = \Big ( \int _{\partial U^t} \,\textrm{d}\lambda _t(x) \Big ) \,\sigma . \end{aligned}$$

By Lemma 4.2, we infer that $\left( \int _{\partial U^t} g(x) \,\textrm{d}\mu _t(x)\right) \tau$ can be represented by a $BV$ function. In particular, decomposing , we infer by uniqueness of the Lebesgue-Besicovitch Decomposition Theorem that
as measures on $[0,\infty )$ so that, for any $g \in C^1_{\textrm{c}}(\Omega )$,

4.5 $$\begin{aligned} \int _{\partial U^t} g(x) \,\textrm{d}\mu _t(x) =0 \quad \tau _{\textrm{sing}}  \textit{--a.e.} \end{aligned}$$

**3**. Set $\mu = \overline{\nabla d \cdot {{\varvec{F}}}}$ and put ${{\tilde{g}}} = D_{|\mu |}\mu$. In order to apply (4.5) with ${{\tilde{g}}}$, we approximate ${{\tilde{g}}}$ by mappings in $C^1_{\textrm{c}}(U)$. By the Lusin Theorem [2, Theorem 1.45, Remark 1.46], we can find a sequence ${\tilde{g}}_k$ of continuous functions in *U* such that

$$\begin{aligned} {{\tilde{g}}}_k(x) \rightarrow {{\tilde{g}}}(x) \quad \text {for }|\mu |\textit{--a.e.}\,\,x \in U\,\, \text{ as } k \rightarrow \infty , \end{aligned}$$

and $|{{\tilde{g}}}_k|\le 1$ holds $|\mu |$–*a.e.*  for each *k*. Then, for any $\delta _k \rightarrow 0,$ $g_k =(\chi _{U^{2\delta _k}}{{\tilde{g}}}_k)_{\delta _k}$ is a sequence of $C^{\infty }_{\textrm{c}}(U)$ functions converging pointwise $|\mu |$–*a.e.*  to ${{\tilde{g}}}$ in *U*, which is also uniformly bounded by the constant 1. Now applying (4.5) with each $g_k$ yields

$$\begin{aligned} \begin{aligned} \int _0^{\infty }\Big |\int _{\partial U^t} {{\tilde{g}}} \,\text {d}\mu _t(x)\Big |\,\text {d}\tau _{\text {sing}}(t)&=\int _0^{\infty }\Big |\int _{\partial U^t} (g_k - {{\tilde{g}}}) \,\text {d}\mu _t(x)\Big |\,\text {d}\tau _{\text {sing}}(t)\\  &\le \int _0^{\infty } \int _{\partial U^t} |g_k - {{\tilde{g}}}|\,\text {d}\mu _t(x) \,\text {d}\tau \\  &= \int _U |g_k - {{\tilde{g}}}|\,\text {d}\mu , \end{aligned} \end{aligned}$$

which tends to zero as $k \rightarrow \infty$ by the Dominated Convergence Theorem. Hence, it follows that

$$\begin{aligned} \int _{\partial U^t} D_{|\mu |}\mu (x) \,\textrm{d}\mu _t(x) = 0 \quad \tau _{\textrm{sing}}  \textit{--a.e.} \end{aligned}$$

By Lemma 3.2, we have

$$\begin{aligned} D_{|\mu |}\mu (x) = D_{|\mu _t|}\mu _t(x) \quad \text {for } \tau \textit{--a.e.}\,\,t \text { and } \mu _t\textit{--a.e.}\,\, x, \end{aligned}$$

so it follows that

$$\begin{aligned} |\mu _t|(\partial U^t) = \int _{\partial U^t} D_{|\mu |}\mu (x) \,\textrm{d}\mu _t(x) = 0 \quad \ \tau _{\textrm{sing}}  \textit{--a.e.} \end{aligned}$$

However, by Theorem 3.1, $|\mu _t|$ is a probability measure supported on $\partial U^t$ for $\tau$–*a.e.* $t \in (0,\infty ),$ so the above can only hold if $\tau _{\textrm{sing}} = 0$.

**4**. By (4.2)–(4.3), we have

$$\begin{aligned} \int _{U} \phi \,\textrm{d}\overline{\nabla d \cdot {{\varvec{F}}}} = \int _0^{\infty } \int _{\partial U^t} \phi \,\textrm{d}({{\varvec{F}}}\cdot \nu )_{\varepsilon } \,\textrm{d}\varepsilon + \int _0^{\infty } \int _{\partial U_t} \phi \,\textrm{d}\mu _t\,\textrm{d}\tau _{\textrm{sing}} \end{aligned}$$

for any bounded Borel function $\phi$ in $\Omega$. Since we have shown that $\tau _{\textrm{sing}} = 0$, the last integral is zero, thus establishing the result. $\square$

### Remark 4.3

  A similar coarea-type formula was recently obtained in [20, Theorem 6.1] in a more general context. Moreover, while it is not explicitly stated, a careful inspection of their argument reveals an alternative proof of Theorem 4.1.

As a consequence, we obtain an alternative proof of the following result from [64], which will be used later. Similar results for Lipschitz-deformable boundaries were proved earlier in [11].

### Theorem 4.4

(Theorem 2.4 in [64])  Let ${{\varvec{F}}}\in \mathcal{D}\mathcal{M}^{{{\,\textrm{ext}\,}}}(\Omega )$ and $U \Subset \Omega$, and let $d(x) = \textrm{dist}(x,\partial U)$. Then

4.6 $$\begin{aligned} \langle {{\varvec{F}}}\cdot \nu , \,\phi \rangle _{\partial U} = \lim _{\varepsilon \rightarrow 0} \frac{1}{\varepsilon } \int _{U \setminus \overline{U^{\varepsilon }}} \phi \,\textrm{d}\overline{\nabla d \cdot {{\varvec{F}}}} \qquad \mathrm{for \,any }\, \phi \in {W}^{1,\infty }(\Omega ). \end{aligned}$$

Moreover, if

$$\begin{aligned} \liminf _{\varepsilon \rightarrow 0} \frac{1}{\varepsilon }\, |\overline{\nabla d \cdot {{\varvec{F}}}}|(U \setminus \overline{U^{\varepsilon }}) < \infty , \end{aligned}$$

then the normal trace $\langle {{\varvec{F}}}\cdot \nu , \,\cdot \,\rangle _{\partial U}$ is represented by a measure on $\partial U$.

### Proof

By Theorem 4.1, we see that

$$\begin{aligned} \int _0^{\infty } \int _{\partial U^t} \psi \,\textrm{d}({{\varvec{F}}}\cdot \nu )_{\partial U^t} \,\textrm{d}t = \int _{U } \psi \,\textrm{d}\overline{\nabla d \cdot {{\varvec{F}}}} \end{aligned}$$

holds for all bounded Borel functions $\psi$ on $\Omega$. Then, taking $\psi = \mathbbm {1}_{U \setminus \overline{U^{\varepsilon }}} \,\phi$ with $\varepsilon >0$ and $\phi \in {W}^{1,\infty }(\Omega )$, we have

4.7 $$\begin{aligned} \frac{1}{\varepsilon } \int _0^{\varepsilon } \phi \,\textrm{d}\overline{\nabla d \cdot {{\varvec{F}}}} = \frac{1}{\varepsilon } \int _0^{\varepsilon } \langle {{\varvec{F}}}\cdot \nu , \,\phi \rangle _{\partial U^t} \,\textrm{d}t. \end{aligned}$$

Since the mapping:

$$\begin{aligned} t \mapsto \langle {{\varvec{F}}}\cdot \nu , \,\phi \rangle _{\partial U^t} = \int _{U^t} \textrm{d}\overline{\nabla \phi \cdot {{\varvec{F}}}} + \int _{U^t} \phi \,\ \textrm{d}(\operatorname {div}{{\varvec{F}}}) \end{aligned}$$

is right-continuous on $[0,\infty )$ for $\phi \in {W}^{1,\infty }(\Omega )$, sending $\varepsilon \rightarrow 0$ in (4.7), we deduce (4.6). Now, for any $\varepsilon _k \rightarrow 0$, the above implies that
as distributions in $\Omega$. If this sequence of measures is uniformly bounded in ${\mathcal {M}}(\Omega )$, by the weak ${}^{*}$–compactness and uniqueness of the limit, we infer that the limiting distribution is also a measure. $\square$

## Localization of the Normal Trace

In this section, we analyze how the normal trace relative to different boundaries can differ. In particular, we seek to understand whether the relation

5.1 $$\begin{aligned} \langle {{\varvec{F}}}\cdot \nu , \,\phi \rangle _{\partial U} = \langle {{\varvec{F}}}\cdot \nu , \,\phi \rangle _{\partial V} \end{aligned}$$

holds for $U, V \subset \Omega$ with overlapping boundary and for any $\phi$ supported in a suitable neighborhood of $\partial U \cap \partial V$. Moreover, if the normal traces of ${{\varvec{F}}}$ on the boundaries of *U* and *V* are represented by measures, one may ask if the equality holds as measures:
5.2This question is motivated by the study of Cauchy fluxes in the sequel. Indeed, it is easily answered whenever the normal trace admits a representation of the form

$$\begin{aligned} \langle {{\varvec{F}}}\cdot \nu , \,\phi \rangle _{\partial U} = \int _{\partial U} \phi (x)\, {{\varvec{F}}}(x) \cdot \nu _{\partial U} \,\textrm{d}{\mathcal {H}}^{n-1}, \end{aligned}$$

which is valid by the classical Gauss-Green formula for a Lipschitz ${{\varvec{F}}}$ and a bounded Lipschitz domain *U*; we see that it is necessary for the associated normals $\nu _{\partial U}$ and $\nu _{\partial V}$ to coincide ${\mathcal {H}}^{n-1}$–*a.e.*  on the common intersection. For bounded divergence-measure fields and sets of finite perimeter, this can be affirmatively answered by using [19, Theorem 5.3] after reformulation to consider suitable notions of measure-theoretic boundaries and normals. However, for a general field ${{\varvec{F}}}$ and open sets *U* and *V*, the normal trace may *fail* to be equal as measures on the common intersection, which is illustrated by the following examples.

### Example 5.1

  Following [11, Example 1.1] which is itself based on [66, §III.14, Example 1], consider the vector field

$$\begin{aligned} {{\varvec{F}}}(x) = \frac{(x_1,x_2)}{x_1^2+x_2^2} \quad \text {for }x=(x_1,x_2) \in {\mathbb {R}}^2, \end{aligned}$$

which lies in $\mathcal{D}\mathcal{M}^1_{{\textrm{loc}}}({\mathbb {R}}^2)$ with $\operatorname {div}{{\varvec{F}}}= 2\pi \delta _0$. Indeed, ${{\varvec{F}}}(x) = 2 \pi \, \nabla \Gamma (x)$, where $\Gamma$ is the fundamental solution for the Laplacian in ${\mathbb {R}}^2$ (see e.g.,  [35, §2.2.1]). Let $H = (0,\infty ) \times {\mathbb {R}}$ be the half-space where $x_1>0$, and let $Q = (0,1) \times (0,1)$ be the unit cube. Then we claim the normal traces of ${{\varvec{F}}}$ on $\partial H$ and $\partial Q$ can be represented by measures that take the form:
5.35.4Indeed, for (5.3), using an unbounded version of [9, Theorem 7.1], for $\phi \in C^{\infty }_{\textrm{c}}({\mathbb {R}}^2)$, we can show that

$$\begin{aligned} \langle {{\varvec{F}}}\cdot \nu , \,\phi \rangle _{\partial H} = -\lim _{\varepsilon \rightarrow 0} \int _{{\mathbb {R}}} \frac{\varepsilon }{\varepsilon ^2 + x_2^2}\, \phi (\varepsilon ,x_2) \,\textrm{d}x_2 = -\pi \phi (0,0), \end{aligned}$$

which implies (5.3). To prove this limit, observe that

$$\begin{aligned} \int _{-\infty }^{\infty } \frac{\varepsilon }{\varepsilon ^2+x_2^2} \phi (0,0) \,\textrm{d}x_2 = \pi \phi (0,0) \quad \text{ for } \text{ any } \varepsilon >0 \end{aligned}$$

and, if $\phi$ is supported in $B_R(0)$, the remainder can be estimated by

$$\begin{aligned} \begin{aligned}&\Big |\int _{{\mathbb {R}}} \frac{\varepsilon }{\varepsilon ^2+x_2^2} ( \phi (\varepsilon ,x_2)-\phi (0,0)) \,\textrm{d}x_2\Big |\\&\quad \le \int _{-R}^R \frac{\varepsilon }{\varepsilon ^2+x_2^2} |\phi (\varepsilon ,x_2) - \phi (0,0) |\, \textrm{d}x_2 + 2 |\phi (0,0)|\int _R^{\infty } \frac{\varepsilon }{\varepsilon ^2 + x_2^2} \,\textrm{d}x_2 \\&\quad \le \Vert \nabla \varphi \Vert _{L^{\infty }({\mathbb {R}}^2)} \int _{-R}^R \frac{\varepsilon }{\sqrt{\varepsilon ^2 + x_2^2}} \,\textrm{d}x_2 + |\phi (0,0)|\,\frac{2\varepsilon }{R}, \end{aligned} \end{aligned}$$

which vanishes as $\varepsilon \searrow 0$. Thus, (5.3) follows.

For the flux on $\partial Q$, we apply Theorem 4.4 near the origin to check that $({{\varvec{F}}}\cdot \nu )_{\partial Q}$ is a measure on $\partial Q$; indeed, we now show that

5.5 $$\begin{aligned} \limsup _{\varepsilon \rightarrow 0} \frac{1}{\varepsilon } \int _{B_{1/2}(0) \cap Q \setminus {\overline{Q}}^{\varepsilon }} |\nabla d \cdot {{\varvec{F}}}|\,\textrm{d}x < \infty . \end{aligned}$$

For this, observe that, for $x \in Q$ satisfying $|x |< \frac{1}{2}$, $d(x) = \textrm{dist}(x,Q)$ satisfies

$$\begin{aligned} \nabla d(x) = {\left\{ \begin{array}{ll} e_1 & \text {if } x_1< x_2, \\ e_2 & \text {if } x_2 < x_1. \end{array}\right. } \end{aligned}$$

Then, for each $\varepsilon >0$, we split the integral in (5.5) into the pieces

$$\begin{aligned} A_{1,\varepsilon }&= \big \{ (x_1,x_2) \in B_{1/2}(0)\, :\, 0< x_1< x_2< \varepsilon \big \}, \\ A_{2,\varepsilon }&= \big \{ (x_1,x_2) \in B_{1/2}(0)\, :\, 0< x_1< \varepsilon< x_2< \frac{1}{2} \big \}, \\ A_{3,\varepsilon }&= \big \{ (x_1,x_2) \in B_{1/2}(0)\, :\, 0< x_2< x_1< \varepsilon \big \}, \\ A_{4,\varepsilon }&= \big \{ (x_1,x_2) \in B_{1/2}(0)\, :\, 0< x_2< \varepsilon< x_1 < \frac{1}{2} \big \}, \end{aligned}$$

so that

$$\begin{aligned} B_{1/2}(0) \cap (Q \setminus {\overline{Q}}^{\varepsilon }) = A_{1,\varepsilon } \cup A_{2,\varepsilon } \cup A_{3,\varepsilon } \cup A_{4,\varepsilon }. \end{aligned}$$

Since $\nabla d = e_1$ on $A_{1,\varepsilon }$ and $A_{2,\varepsilon }$, we can compute

5.6 $$\begin{aligned} \begin{aligned} \frac{1}{\varepsilon } \int _{A_{1,\varepsilon }} |\nabla d \cdot {{\varvec{F}}}|\,\textrm{d}x&\le \frac{1}{\varepsilon } \int _0^{\varepsilon } \int _{0}^{x_2} \frac{x_1}{x_1^2+x_2^2} \,\textrm{d}x_1 \,\textrm{d}x_2 = \frac{1}{2} \log 2, \end{aligned} \end{aligned}$$

and

5.7 $$\begin{aligned} \begin{aligned} \frac{1}{\varepsilon } \int _{A_{2,\varepsilon }} |\nabla d \cdot {{\varvec{F}}}|\,\textrm{d}x&\le \frac{1}{\varepsilon } \int _{\varepsilon }^{\frac{1}{2}} \int _0^{\varepsilon } \frac{x_1}{x_1^2+x_2^2} \,\textrm{d}x_1 \,\textrm{d}x_2 \\&= \frac{1}{4\varepsilon } \log (1+4\varepsilon ^2) - \frac{1}{2} \log 2 + \arctan (\frac{1}{2\varepsilon }) - \frac{\pi }{4}, \end{aligned} \end{aligned}$$

by a direct calculation. Both terms remain bounded as $\varepsilon \rightarrow 0$, since $\arctan (x) \le \frac{\pi }{2}$ and $\log (1+x) \le x$ for all $x \ge 0$. Moreover, the integrals over $A_{3,\varepsilon }$ and $A_{4,\varepsilon }$ coincide with the integrals in (5.6) and (5.7), respectively, by swapping the coordinates. Then the claim follows.

Therefore, $({{\varvec{F}}}\cdot \nu )_{\partial Q}$ is a measure on $\partial Q$, which agrees with  on $\partial Q {\setminus } \{0\}$, by using Theorem 5.3 below and since ${{\varvec{F}}}$ is smooth there. Then there is $c_0 \in {\mathbb {R}}$ such that . This constant can be determined by noting that

$$\begin{aligned} ({{\varvec{F}}}\cdot \nu )_{\partial Q}(\partial Q) = c_0 + \frac{\pi }{2} = \int _{ Q} \textrm{d}(\operatorname {div}{{\varvec{F}}}) = 0, \end{aligned}$$

so that $c_0 = -\frac{\pi }{2}$, establishing (5.4). Then we have
Thus, the respective normal traces concentrate at the corner point (0, 0) and take different values there. This shows that (5.2) may fail in general.

The above example is for a field ${{\varvec{F}}}$ that is singular at the origin, and the discrepancy arose due to the concentration of the normal trace at a point. One may also encounter obstructions arising from the regularity of the domains, which is illustrated in the following example:

### Example 5.2

  In ${\mathbb {R}}^2$, we can consider the domains:

$$\begin{aligned} V = B_1(0) \setminus \{ (x_1,0)\,:\, x_1 \ge 0 \}, \quad U = \{x \in V\,:\,x_2 > 0 \} = B_1(0)^+. \end{aligned}$$

Then we take ${{\varvec{F}}}(x) = e_2 = (0,1)$, which is smooth and hence lies in ${\mathcal {D}}{\mathcal {M}}^{\infty }_{\textrm{loc}}({\mathbb {R}}^2)$. Set $A = \{x \in B_1(0): x_1 > 0 \}$. Then $A \cap \partial U = A \cap \partial V = \{ (x_1,0):\,0< x_1 < 1\}$. However, we have
so the associated normal traces do not coincide, even though $U \subset V$. Indeed, the normal trace on $\partial U \cap A$ is understood in the classical sense, since *U* is a Lipschitz domain. For $\partial V$, using the definition, we can compute the normal trace for $\phi \in {W}^{1,\infty }_{\textrm{c}}({\mathbb {R}}^2)$ as

$$\begin{aligned} \begin{aligned} \langle {{\varvec{F}}}\cdot \nu , \,\phi \rangle _{\partial V}&= -\int _V \phi \,\operatorname {div}{{\varvec{F}}}\,\textrm{d}x - \int _V \nabla \phi \cdot {{\varvec{F}}}\,\textrm{d}x \\&= -\int _{B_1(0)} \phi \, \operatorname {div}{{\varvec{F}}}\,\textrm{d}x - \int _{B_1(0)} \nabla \phi \cdot {{\varvec{F}}}\,\textrm{d}x \\&= \langle {{\varvec{F}}}\cdot \nu , \,\phi \rangle _{\partial B_1(0)}, \end{aligned} \end{aligned}$$

by noting that ${\mathcal {L}}^2(B_1(0) \setminus V) = 0$, so the normal trace indeed vanishes on *A*.

In general, one may still hope for (5.1) to hold on *relatively open* portions of the common boundary, which will be sufficient for our later purposes. More precisely, we have

### Theorem 5.3

Let $\Omega \subset {\mathbb {R}}^n$ be open and ${{\varvec{F}}}\in \mathcal{D}\mathcal{M}^{{{\,\textrm{ext}\,}}}(\Omega )$. Given $U, V \Subset \Omega$, let $A \subset {\mathbb {R}}^n$ be open such that

5.8 $$\begin{aligned} U \cap A = V \cap A. \end{aligned}$$

Then $$A \cap \partial U = A \cap \partial V$$ and

$$\begin{aligned} \langle {{\varvec{F}}}\cdot \nu , \,\phi \rangle _{\partial U} = \langle {{\varvec{F}}}\cdot \nu , \,\phi \rangle _{\partial V} \quad \text {for any } \phi \in {W}^{1,\infty }_{\textrm{c}}(A). \end{aligned}$$

In particular, if the normal traces on $\partial U$ and $\partial V$ are represented by measures, then
5.9

### Proof

  Replacing *A* by $A \cap \Omega$ if necessary, we can assume that $A \subset \Omega$. We first show that $A \cap \partial U = A \cap \partial V$. Indeed, if $x \in A \cap \partial U$, since *U* is open, then $x \notin U$. Moreover, since *A* is open, there is a sequence $\{x_k\} \subset A \cap U$ such that $x_k \rightarrow x$. However, by (5.8), each $x_k \in V$ while $x \notin V$, so $x \in \partial V$ and the latter inclusion follows by symmetry.

Now, for any $\delta >0$, when $\varepsilon \in (0,\delta )$, we claim that

$$\begin{aligned} A^{\delta } \cap (U \setminus \overline{U^{\varepsilon }}) = A^{\delta } \cap (V \setminus \overline{V^{\varepsilon }}), \end{aligned}$$

and that $d_U = d_V$ on this common intersection. Indeed, if $x \in A^{\delta } \cap (U \setminus \overline{U^{\varepsilon }})$, there is $y \in \partial U$ such that $|x - y |< \varepsilon$. Since $\varepsilon <\delta$, we see that $y \in A$ and so $y \in \partial V$, giving

$$\begin{aligned} d_V(x) \le |x - y |< \varepsilon . \end{aligned}$$

Hence, $x \in A^{\delta } \cap (V \setminus \overline{V^{\varepsilon }})$, and taking the infimum over all such *y* yields that $d_V(x) \le d_U(x)$. By symmetry of *U* and *V*, the claim follows.

In particular,
by definition of the pairing from Theorem 2.7. Hence, by Theorem 4.4, it follows that, for any $\phi \in {W}^{1,\infty }_{\textrm{c}}(A^{\delta })$,

$$\begin{aligned} \begin{aligned} \langle {{\varvec{F}}}\cdot \nu , \,\phi \rangle _{\partial U}&= \lim _{\varepsilon \rightarrow 0} \frac{1}{\varepsilon } \int _{U \setminus \overline{U^{\varepsilon }}} \phi \,\textrm{d}\overline{\nabla d_U \cdot {{\varvec{F}}}} \\&= \lim _{\varepsilon \rightarrow 0} \frac{1}{\varepsilon } \int _{V \setminus \overline{V^{\varepsilon }}} \phi \,\textrm{d}\overline{\nabla d_V \cdot {{\varvec{F}}}} = \langle {{\varvec{F}}}\cdot \nu , \,\phi \rangle _{\partial V}. \end{aligned} \end{aligned}$$

Since $\delta >0$ is arbitrary, it follows that the above holds for any $\phi \in {W}^{1,\infty }_{\textrm{c}}(A)$. If the normal traces are represented by measures, (5.9) follows by a standard density argument. $\square$

### Remark 5.4

  One may wonder if the condition: $U \cap A = V \cap A$ can be modified to hold at the level of boundaries, that is, to impose $\partial U \cap A = \partial V \cap A$ instead. However, Example 5.2 shows that this is insufficient, even if the additional assumption that $U \subset V$ is made.

We conclude this section by recording that a maximal portion of the common intersection $\partial U \cap \partial V$ can be defined, where Theorem 5.3 applies; that is, we can define

$$\begin{aligned} S(U,V) = \{ x \in \partial U \cap \partial V\,:\,B_{\varepsilon }(x) \cap U = B_{\varepsilon }(x) \cap V \text { for some } \varepsilon >0 \}. \end{aligned}$$

Indeed, if *A* is any open set such that $A \cap U = A \cap V$, then, for every $x \in A \cap \partial U \cap \partial V$, there is $\varepsilon >0$ such that $B_{\varepsilon }(x) \subset A$. It follows that $x \in S(U,V)$ so that $A \cap \partial U \cap \partial V \subset S(U,V)$. Conversely, we can take ${{\tilde{A}}} = \bigcup _{x \in S(U,V)} B_{\varepsilon _x}(x)$, where $\varepsilon _x>0$ is as in the definition of set *S*(*U*, *V*).

## Cauchy Flux I: Main Results and Connections

The balance law postulates that the production of a quantity in any open set $U \Subset \Omega$ is balanced by the Cauchy flux of this quantity through boundary $\partial U$ of *U*. We assume the production is represented by a finite (signed) Radon measure $\sigma$ in $\Omega$ such that

$$\begin{aligned} \sigma (U) = {\mathcal {F}}(\partial U) \quad \text {for any } U \Subset \Omega , \end{aligned}$$

where ${\mathcal {F}}$ is the flux through the boundary of *U*. In this section, we introduce the conditions on the Cauchy flux ${\mathcal {F}}$ that guarantee the existence of an extended divergence-measure vector field ${{\varvec{F}}}$ satisfying

$$\begin{aligned} -\operatorname {div}{{{\varvec{F}}}}= \sigma \end{aligned}$$

and such that ${\mathcal {F}}$ can be recovered locally, on the boundary of *almost every open set*, through the measure normal trace of ${{\varvec{F}}}$. We begin by presenting the existing developments in this direction and emphasizing the remaining difficulties, before stating our main results.

### Connections to Other Formulations of the Cauchy Flux

The origin of the study of Cauchy fluxes dates back to the fundamental paper by Cauchy [6], who, in 1823, considered the balance law in a bounded domain $\Omega$ in Classical Physics:

6.1 $$\begin{aligned} \int _{U} p(x) \, \textrm{d}x = \int _{\partial U} f(x, \nu (x)) \,\textrm{d}{\mathcal {H}}^{n-1}(x) \quad \text{ for } \text{ any } U \subset \Omega . \end{aligned}$$

Here the production *p*(*x*) is a bounded function in $x \in \Omega$, and the *density function* $f(x,\nu )$ of the flux depends on point $x\in \partial U$ and the corresponding interior unit normal $\nu$. It was shown in [6] that, if *f* is continuous in *x*, then *f* must be linear in $\nu$. For self-containedness and subsequent development, we now present a brief description of Cauchy’s argument.

#### Theorem 6.1

(Cauchy’s tetrahedron argument) Consider the classical balance law (6.1) in ${\mathbb {R}}^n$, where *f* is continuous in *x* and $p \in L^{\infty }(\Omega )$. Then there exists a continuous vector field ${{\varvec{F}}}$ such that

$$\begin{aligned} f(x, \nu (x)) = {{\varvec{F}}}(x) \cdot \nu (x) . \end{aligned}$$

#### Proof

  Consider the standard orthonormal basis $\{e_i\}_{i=1}^n$ of ${\mathbb {R}}^n$. Let $\nu = (\nu _1,\nu _2,\cdots ,\nu _n)$ be any unit normal vector satisfying $\nu \cdot e_i \ne 0$ for each $1 \le i \le n$. Fix $\epsilon >0$ and consider the tetrahedron $T_{\varepsilon }$ with vertices $v_0 = 0$, $v_i = \varepsilon \frac{\nu _n}{\nu _i} e_i$ for each $1 \le i \le n-1$, and $v_n = \varepsilon e_n$. Then the boundary of $T_{\varepsilon }$ is composed of $(n+1)$ faces, denoted by $S_{i,\varepsilon }$ for $1 \le i \le n$ and $S_{\varepsilon }$. These are chosen so that $S_{i,\varepsilon }$ are contained in the planes: $x_i=0$, $1 \le i \le n$, and $S_{\varepsilon }$ is contained in the plane perpendicular to $\nu$ with the equation: $\sum _{i=1}^n \nu _i x_i= \varepsilon \nu _n$. For ${\tilde{x}} \in {\mathbb {R}}^n$, we consider the translated tetrahedron $T_{{\tilde{x}},\varepsilon }:= {\tilde{x}} + T_{\varepsilon }$, whose faces are denoted as $S_{{{\tilde{x}}},i,\varepsilon }$ and $S_{{{\tilde{x}}},\varepsilon }$.

Then the balance law gives

6.2 $$\begin{aligned} \int _{T_{{{\tilde{x}}}, \varepsilon }} p(x) \,\textrm{d}x = \sum _{i=1}^n \int _{S_{{{\tilde{x}}},i,\varepsilon }} f(x,e_i) \,\textrm{d}{\mathcal {H}}^{n-1} + \int _{S_{{{\tilde{x}}},\varepsilon }} f(x,-\nu ) \,\textrm{d}{\mathcal {H}}^{n-1}, \end{aligned}$$

where we split the boundary integral in terms of the contributions on the $(n+1)$ faces. Since the production is bounded, we can estimate

$$\begin{aligned} \Big |\int _{T_{{{\tilde{x}}},\varepsilon }} p(x) \,\textrm{d}x \Big |\le \Vert p \Vert _{L^{\infty }(\Omega )} {\mathcal {L}}^n(T_{{{\tilde{x}}},\varepsilon }). \end{aligned}$$

We then divide both sides of (6.2) by ${\mathcal {H}}^{n-1}(S_{{{\tilde{x}}},\varepsilon })$ and observe that

$$\begin{aligned} (\nu \cdot e_i) {\mathcal {H}}^{n-1} (S_{{{\tilde{x}}},\varepsilon })= {\mathcal {H}}^{n-1}(S_{{{\tilde{x}}},i, \varepsilon }) \end{aligned}$$

to deduce

6.3 $$\begin{aligned} \begin{aligned}&\left|\frac{\int _{S_{{{\tilde{x}}},\varepsilon }}f(x,-\nu ) \textrm{d}{\mathcal {H}}^{n-1}(x)}{{\mathcal {H}}^{n-1} (S_{{{\tilde{x}}},\varepsilon })} + \sum _{i=1}^n (\nu \cdot e_i) \frac{\int _{S_{{{\tilde{x}}},i,\varepsilon }}f(x, e_i) \textrm{d}{\mathcal {H}}^{n-1}(x)}{{\mathcal {H}}^{n-1}(S_{\tilde{x},i,\varepsilon })} \right|\\&\quad \le \Vert p \Vert _{L^{\infty }(\Omega )} \frac{{\mathcal {L}}^{n}(T_{{\tilde{x}},\varepsilon })}{{\mathcal {H}}^{n-1} (S_{{{\tilde{x}}},\varepsilon })} =C(n)\Vert p \Vert _{L^{\infty }(\Omega )} \, \varepsilon . \end{aligned} \end{aligned}$$

We let $\varepsilon \rightarrow 0$ in (6.3) and use the fact that $x \mapsto f(x,\nu )$ is continuous to obtain

6.4 $$\begin{aligned} f({\tilde{x}},-\nu ) + \sum _{i=1}^{n} \nu _i f({\tilde{x}}, e_i)=0. \end{aligned}$$

Define a vector field ${{\varvec{F}}}=(F_1,F_2,\cdots ,F_n)$ by

$$\begin{aligned} F_i(x): = f(x,e_i) \quad \text{ for } x \in \Omega . \end{aligned}$$

Since ${\tilde{x}} \in \Omega$ is arbitrary in (6.4), then ${{\varvec{F}}}$ satisfies

6.5 $$\begin{aligned} f(x,-\nu )= -{{\varvec{F}}}(x) \cdot \nu \end{aligned}$$

for any $x \in \Omega$ and $\nu \in {\mathbb {R}}^n$ such that $\nu _i \ne 0$ for all $1 \le i \le n$. To extend this to hold for any $\nu$, we can choose a different orthonormal basis $\{{{\tilde{e}}}_i\}_{i=1}^n$ and work in the associated coordinate system $({{\tilde{x}}}_1, \cdots , {{\tilde{x}}}_n)$ to allow for $\nu \in {\mathbb {R}}^n$ satisfying $\nu \cdot {{\tilde{e}}}_i \ne 0$ for all $1 \le i \le n$ in (6.5). Since *f* is continuous, it follows that ${{\varvec{F}}}$ is also continuous. $\square$

The above derivation assumes the existence of a continuous density *f*. However, in applications, we may naturally encounter solutions that are discontinuous or singular, thereby violating this continuity hypothesis. It was not until 1959 that Noll in [52] considered the problem of removing the continuity assumption of *f* and proposed an axiomatic scheme for Continuum Mechanics, which reduces to the familiar balance law for contact forces in the stationary case. In this setting, the body $\Omega$ is composed of parts that are sets with smooth boundary. In [52, Theorem IV], it was shown that the density function should depend only on the position *x* and the normal vector $\nu (x)$, independent of other properties of the surface such as the curvature. However, additional conditions are necessary to derive the linearity of *f*, as it was later remarked in [51, p. 79] that “*it is unfortunate that nobody has been able, so far, to *[*establish the linearity of f*]* under *[*the proposed axioms*]* without the ad-hoc continuity assumption...*”.

From Classical Mechanics, it follows that the object of study should be the total flux across a surface *S* contained in $\partial U$, that is,

6.6 $$\begin{aligned} {\mathcal {F}}(S)= \int _{S} f(x, \nu (x)) \,\textrm{d}{\mathcal {H}}^{n-1}(x). \end{aligned}$$

This viewpoint of studying the *Cauchy flux* ${\mathcal {F}}$ was proposed by Gurtin & Martin in [43]. Since they assumed the production *p* is bounded, the balance law (6.1) immediately implies the inequality:

6.7 $$\begin{aligned} |{\mathcal {F}} (\partial U)|\le K{\mathcal {L}}^{n}(U) \quad \text{ for } \text{ any } U \subset \Omega . \end{aligned}$$

Moreover, from (6.6), it also follows that the flux should be an additive function in the sense that

6.8 $$\begin{aligned} {\mathcal {F}} (S_1 \cup S_2) = {\mathcal {F}}(S_1) + {\mathcal {F}} (S_2) \quad \text{ for } \text{ disjoint } S_1, S_2 \subset \partial U. \end{aligned}$$

Therefore, Gurtin & Martins in [43] considered an additive set function ${\mathcal {F}}$ defined on (sufficiently regular) oriented surfaces $(S,\nu )$ which satisfies the properties of being *area-bounded*,

6.9 $$\begin{aligned} |{\mathcal {F}}(S)|\le C {\mathcal {H}}^{n-1}(S), \end{aligned}$$

and *weakly volume-bounded* in the sense that (6.7) holds. Under these assumptions, they deduced the existence of a density function $f(x,\nu )$ defined for ${\mathcal {L}}^n$–*a.e.* $x \in \Omega$ and all unit vectors $\nu$ for which (6.6) holds for every admissible surface. Moreover, the following linearity of *f* was proved: There exists a field ${{\varvec{F}}}\in L^{\infty }$ such that

$$\begin{aligned} f(x,\nu )= {{\varvec{F}}}(x) \cdot \nu \quad \text {for }{\mathcal {L}}^n\textit{--a.e.}\,\, x \in \Omega . \end{aligned}$$

Furthermore, the following uniform average density condition on ${\mathcal {F}}$ was introduced:

6.10 $$\begin{aligned} \{f_r(x,\nu )\}_{r >0} \quad \text{ with } f_r(x,\nu ):= \frac{ {\mathcal {F}} (D_r(x,\nu ))}{{\mathcal {H}}^{n-1}(D_r(x,\nu ))}. \end{aligned}$$

This converges uniformly as $r \rightarrow 0$ on any compact set $K \subset \Omega$, where $D_r(x,\nu )$ is the $(n-1)$-dimensional disk of radius *r* centered at *x* with normal $\nu$. It was proved that (6.10) is a necessary and sufficient condition for the Cauchy flux ${\mathcal {F}}$ to have a continuous density *f* that is linear for every *x*.

The class of admissible surfaces was extended by Ziemer in [67] to consider boundaries of sets of finite perimeter. We refer the reader to [50] for a detailed exposition on the theory of sets of finite perimeter, which gives a natural class of admissible surfaces that are closed under the set operations and provides a suitable measure-theoretic notion of the normal. Under the same assumptions (6.7)–(6.8), considered on sets of finite perimeter, the existence of a bounded density $f(x,\nu )$ satisfying (6.6) on every oriented surface *S* was established in [67, Theorem 3.3]. Once the existence of a density was established, the linearity of the flux followed from the results in [43]. Indeed, in both [43] and [67], the existence of the vector field is ultimately based (after suitable mollifications) on the *Cauchy’s tetrahedron argument*, as sketched above in Theorem 6.1. Moreover, it was proved in [67] that the constructed vector field ${{\varvec{F}}}\in L^{\infty }$ satisfies $\operatorname {div}{{\varvec{F}}}\in L^{\infty }$ and that the Gauss-Green formula

$$\begin{aligned} \int _{Q} \textrm{d}(\operatorname {div}{{\varvec{F}}}) = -\int _{\partial Q} {{\varvec{F}}}(x) \cdot \nu (x)\, \textrm{d}{\mathcal {H}}^{n-1}(x) \end{aligned}$$

holds for almost all cubes $Q = (a_1,b_1) \times \cdots \times (a_n,b_n)$.

The boundedness conditions (6.7) and (6.9) were relaxed by Šilhavý in [60, 61] to allow the Cauchy fluxes of the form:

6.11 $$\begin{aligned}&|{\mathcal {F}}(S) |\le \int _S h \,\textrm{d}{\mathcal {H}}^{n-1}, \end{aligned}$$

6.12 $$\begin{aligned}&|{\mathcal {F}}(\partial _{*} M)|\le \int _{M} k \,\textrm{d}x, \end{aligned}$$

for $h, k \in L^p$ and any Borel set $S \subset \partial _*M$, where *M* is assumed to be a normalized set of finite perimeter in $\Omega$ and $\partial _{*}M$ is the measure-theoretic normal. We denote the collection of such *M* by ${\mathscr {P}}$. We note that (6.11) holds only for a particular representative *h* in the $L^p$ class, which encodes fine properties of the underlying field, and the right-hand side may fail to be finite in general. As such the class of admissible surfaces must be relaxed to “almost all” surfaces, which is made precise by the class

6.13 $$\begin{aligned} {\mathscr {P}}_h = \left\{ M \in {\mathscr {P}}\, : \,\, M\Subset \Omega \, \text{ and } \,\int _{\partial _{*}M} h \,\textrm{d}{\mathcal {H}}^{n-1} < \infty \right\} . \end{aligned}$$

This leads to the establishment of a one-to-one correspondence between Cauchy fluxes and $L^p$–integrable fields ${{\varvec{F}}}$ with $L^p$ divergence in [61, Theorem 5.1]. It is shown that conditions (6.11)–(6.12) imply the existence of a vector field in $L^p$.

However, the $L^p$–regularity of the divergence rules out the important physical behavior where the Cauchy flux may *jump* across the boundary. One may naturally wonder whether the case of divergence-measure fields can be treated in this framework. This was answered by Degiovanni, Marzocchi & Musesti [31], providing a definition of Cauchy fluxes representable by $\mathcal{D}\mathcal{M}^p$–fields. For this, the upper bound (6.11) for the flux remains the same, while (6.12) is replaced by the measure bound:

$$\begin{aligned} |{\mathcal {F}}(\partial _{*}M) |\le \lambda (M) \end{aligned}$$

for some non-negative Radon measure $\lambda$ on $\Omega$. In this setting, the flux is recovered on the class of surfaces

$$\begin{aligned} {\mathscr {P}}_{h,\lambda } = \Big \{ M \in {\mathscr {P}}\,:\,M \Subset \Omega ,\,\, \int _{\partial _{*}M} h \,\textrm{d}{\mathcal {H}}^{n-1} < \infty ,\,\, \lambda (\partial _*M) = 0\Big \}. \end{aligned}$$

However, in general, the condition $\lambda (\partial _{*}M)=0$ rules out surfaces along which ${\mathcal {F}}$ has a jump, say, ${\mathcal {F}}(-\partial _{*}M) \ne -{\mathcal {F}}(\partial _*M)$. This requirement was removed by Schuricht [59], where the flux is defined for all subsets in ${\mathscr {P}}_h$, where a formulation in terms of contact interactions between two bodies is proposed.

We also refer to [19] for bounded divergence-measure fields in which *every* set of finite perimeter can be treated and to [9] in which the limit formula for any general open subset is obtained.

Nevertheless, a characterization of Cauchy fluxes in the extended case has still been missing. Note that a formulation was proposed in [63] in which the flux is defined only on planar polyhedra and involves the estimates valid for *almost all* translates, making the hypotheses difficult to verify. Instead, we now seek a construction in the spirit of [31, 43, 59, 61] that is also sufficiently robust to treat jumps across boundaries and one-sided singularities.

### Formulation of the Cauchy flux and statement of the main theorem for the Cauchy flux

We begin by precisely defining the admissible class of surfaces under consideration. This is because the class ${\mathscr {P}}_h$ from (6.13) has seemingly no natural replacement when *h* is replaced by a Radon measure $\mu$ and, moreover, we wish to depart from sets of finite perimeter and consider general open sets.

#### Definition 6.2

  For a non-negative and finite Radon measure $\mu$ in $\Omega$, denote that

6.14 $$\begin{aligned} {\mathcal {O}}_{\mu }:=\Big \{ U \Subset \Omega \,\,\,\text{ open }\, :\, \liminf _{\varepsilon \rightarrow 0} \frac{1}{\varepsilon }\,\mu \left( \{ x \in U\,:\,\textrm{dist}(x,\partial U)< \varepsilon \}\right) < \infty \Big \}. \end{aligned}$$

This condition is motivated by Theorem 4.4. We see later as a consequence of Lemma 7.4 that, for any open set $U \Subset \Omega$, $U^{\varepsilon } \in {\mathcal {O}}_{\mu }$ for ${\mathcal {L}}^1$–*a.e.* $\varepsilon >0$. Thus, in this sense, ${\mathcal {O}}_{\mu }$ contains *almost all* open sets.

#### Remark 6.3

  Recall that, for $U \subset \Omega$ and $\varepsilon >0,$ we write

6.15 $$\begin{aligned} U^{\varepsilon } = \{ x \in U\,:\,\textrm{dist}(x,\partial U) > \varepsilon \}. \end{aligned}$$

We also set $U^0 = U$ and

$$\begin{aligned} U^{-\varepsilon } = \{ x \in {\mathbb {R}}^n \,:\,\textrm{dist}(x,U) < \varepsilon \}. \end{aligned}$$

Using this notation, we can equivalently formulate (6.14) as

6.16 $$\begin{aligned} \liminf _{\varepsilon \rightarrow 0}\, \frac{\mu (U) - \mu ({\overline{U}}^{\varepsilon })}{\varepsilon } = \liminf _{\varepsilon \rightarrow 0}\, \frac{1}{\varepsilon }\,\mu (U \setminus \overline{U^{\varepsilon }}) < \infty . \end{aligned}$$

Notice that $\mu (\partial U^{\varepsilon }) = 0$ for ${\mathcal {L}}^1$–*a.e.* $\varepsilon >0$ and $\varepsilon \mapsto \mu ({{\overline{U}}}^{\varepsilon })$ is non-increasing and left-continuous. If $\varepsilon _k \rightarrow 0$ is a sequence realizing this limit inferior in (6.16), then, for each *k*, we can find $0<{{\tilde{\varepsilon }}}_k \le \varepsilon _k$ such that $\mu ({{\overline{U}}}^{{{\tilde{\varepsilon }}}_k}) - \frac{\varepsilon _k}{k} \le \mu ({{\overline{U}}}^{\varepsilon _k}) \le \mu ({{\overline{U}}}^{{{\tilde{\varepsilon }}}_k})$ and $\mu (\partial U^{{{\tilde{\varepsilon }}}_k})=0$ for each *k*. Thus, we always have

$$\begin{aligned} \liminf _{\varepsilon \rightarrow 0}\, \frac{\mu (U) - \mu ({\overline{U}}^{\varepsilon })}{\varepsilon } =\liminf _{\varepsilon \rightarrow 0}\, \frac{\mu (U) - \mu (U^{\varepsilon })}{\varepsilon }, \end{aligned}$$

regardless of whether both sides are finite or not. In addition, we may also consider the ${\mathcal {O}}_{\mu }$–condition for subsets $U \subset {\mathbb {R}}^n$ that are not necessarily compactly contained in $\Omega$; for this, we simply extend measure $\mu$ by zero to ${\mathbb {R}}^n$.

#### Definition 6.4

  A *Cauchy flux* in $\Omega$ is a mapping ${\mathcal {F}}$ defined on pairs (*S*, *U*) with $S \subset \partial U$ Borel and $U \Subset \Omega$ open, so that the balance law is satisfied:

6.17 $$\begin{aligned} {\mathcal {F}}_{U}(\partial U)=\sigma (U)\quad \text { for any }U \Subset \Omega , \end{aligned}$$

and there is a non-negative finite Radon measure $\mu$ such that the following conditions hold:

(i) Additivity property: For any $U \in {\mathcal {O}}_{\mu }$ and disjoint Borel subsets $S,T \subset \partial U$,
  $$\begin{aligned} {\mathcal {F}}_{ U}(S \cup T) = {\mathcal {F}}_{U}(S) + {\mathcal {F}}_{U}(T). \end{aligned}$$
(ii) Localization property: For any $U,V \in {\mathcal {O}}_{\mu }$ and any open set $A \subset \Omega$ such that $A \cap U = A \cap V$, then
  $$\begin{aligned} {\mathcal {F}}_{U}(A \cap \partial U) = {\mathcal {F}}_{V}(A \cap \partial V). \end{aligned}$$
(iii) Upper bound: For any $U \in {\mathcal {O}}_{\mu }$,
  6.18 $$\begin{aligned} |{\mathcal {F}}_{U}(S)|\le \mu ^{n-1}_{U}\left( S \right) \end{aligned}$$
  holds for any Borel set $S \subset \partial U$ and any limiting measure

In Remark 7.5, we will draw some connections to Condition (iii) in relation to the existing formulations in the $L^p$ setting. However, our condition appears to be fundamentally different to the class ${\mathscr {P}}_h$ that appears in the previously existing formulations.

#### Remark 6.5

  In Condition (iii), since $U \in {\mathcal {O}}_{\mu }$, then there is a sequence $\varepsilon _k \searrow 0$ for which the non-negative measure sequence:
satisfies

$$\begin{aligned} \lim _{k \rightarrow \infty } \nu _k(\Omega ) = \liminf _{\varepsilon \rightarrow 0} \frac{1}{\varepsilon } \, \mu \left( U \setminus \overline{U^{\varepsilon }}\right) < \infty . \end{aligned}$$

Thus, $\{\nu _k\}$ is uniformly bounded in ${\mathcal {M}}(\Omega )$ and, up to a subsequence, converges weakly ${}^*$ to a measure $\nu$. Moreover, since each $\nu _k$ vanishes on $(\Omega \setminus {\overline{U}}) \cup U^{\varepsilon _k}$ for each *k*, the limit measure $\nu$ is supported on $\partial U$. Therefore, such a limiting measure $\mu ^{n-1}_{U}$ always exists, even though it is non-unique in general, and we impose (6.18) for every limiting measure obtained in this way. However, it turns out that this limiting measure is unique on *most* surfaces, which will be made precise in Lemma 7.4.

The main result of this section is that Definition 6.4 implies the existence of a vector-valued Radon measure ${{\varvec{F}}}=(F_1,\cdots ,F_n)$ in $\Omega$, whose normal trace corresponds to ${\mathcal {F}}$. More precisely, we prove the following main theorem for the Cauchy flux:

#### Theorem 6.6

Let ${\mathcal {F}}$ be a Cauchy flux in $\Omega$. Then there exists a unique divergence-measure field ${{\varvec{F}}}\in \mathcal{D}\mathcal{M}^{{{\,\textrm{ext}\,}}}(\Omega )$ such that

$$\begin{aligned} -\operatorname {div}{{\varvec{F}}}= \sigma \end{aligned}$$

and

6.19 $$\begin{aligned} {\mathcal {F}}_{U}(S) = ({{\varvec{F}}}\cdot \nu )_{\partial U}(S) \end{aligned}$$

for any $U \in {\mathcal {O}}_{\mu }$ and any Borel set $S \subset \partial U$, where the measure $({{\varvec{F}}}\cdot \nu )_{\partial U}$ is the normal trace of ${{\varvec{F}}}$ on $\partial U$. Conversely, every ${{\varvec{F}}}\in \mathcal{D}\mathcal{M}^{{{\,\textrm{ext}\,}}}(\Omega )$ defines a Cauchy flux, by taking $\mu = |{{\varvec{F}}}|$ and $\sigma = -\operatorname {div}{{\varvec{F}}}$.

This makes precise the one-to-one correspondence

$$\begin{aligned} \left\{ \text {Cauchy fluxes } {\mathcal {F}} \text { in } \Omega \right\} \,\longleftrightarrow \, \mathcal{D}\mathcal{M}^{{{\,\textrm{ext}\,}}}(\Omega ) \end{aligned}$$

as we claimed in the introduction, and the local recovery is now precisely formulated via the class ${\mathcal {O}}_{\mu }$. We point out that there is no hope to obtain the recovery on all open sets *U*, as the normal trace appearing in (6.19) is not generally a measure on arbitrary open sets.

The converse statement of Theorem 6.6 is a consequence of the properties of the normal trace established in earlier sections, which we record here.

#### Theorem 6.7

Let $${{\varvec{F}}}\in \mathcal{D}\mathcal{M}^{{{\,\textrm{ext}\,}}}(\Omega )$$. Define

6.20 $$\begin{aligned} {\mathcal {F}}_{U}(S) := ({{\varvec{F}}}\cdot \nu )_{\partial U}(S) \end{aligned}$$

for any open set $U \Subset \Omega$ and any Borel set $S \subset \partial U$, whenever the normal trace $({{\varvec{F}}}\cdot \nu )_{\partial U}$ restricts to a measure on $\partial U$. Furthermore, for $U \Subset \Omega$, define globally

6.21 $$\begin{aligned} {\mathcal {F}}_{U}(\partial U) := \langle {{\varvec{F}}}\cdot \nu , \,\mathbbm {1}_{\Omega } \rangle _{\partial U}. \end{aligned}$$

Then ${\mathcal {F}}$ defines a Cauchy flux in the sense of Definition 6.4, with $\sigma = -\operatorname {div}{{\varvec{F}}}$ and any non-negative Radon measure $\mu$ such that $|{{\varvec{F}}}|\le \mu$.

#### Proof

  By definition of the normal trace, it follows that ${\mathcal {F}}_{U}(\partial U) = -\operatorname {div}{{\varvec{F}}}(U)$. Using (2.11), we see that the balance law holds for all $U \Subset \Omega$. Also, if $({\mathcal {F}}\cdot \nu )_{\partial U}$ is represented by a measure on $\partial U$, then (6.20) and (6.21) coincide when $S=\partial U$.

By Theorem 4.4, the normal trace $({{\varvec{F}}}\cdot \nu )_{\partial U}$ is represented by a measure whenever $U \in {\mathcal {O}}_{|{{\varvec{F}}}|}$, property (i) follows from the fact that ${\mathcal {O}}_{\mu } \subset {\mathcal {O}}_{|{{\varvec{F}}}|}$. The localization property (ii) follows from Theorem 5.3. Finally, for (iii), for any $U \in {\mathcal {O}}_{\mu }$, it follows from Remark 6.5 that there exists both $\varepsilon _k \rightarrow 0$ and a limiting measure $\mu _U^{n-1}$ such that
Since $|\overline{\nabla d \cdot {{\varvec{F}}}} |\le |{{\varvec{F}}}|\le \mu$ by (2.5) from the product rule, we see from Theorem 4.4 that
which implies that $|({{\varvec{F}}}\cdot \nu )_{\partial U}|\le \mu _{U}^{n-1}$ as measures. Therefore, ${\mathcal {F}}$ is a Cauchy flux. $\square$

The main implication of Theorem 6.6 will be proven in several steps, which will be broken up into separate theorems. After establishing several useful properties of the flux in Sect. 7, we will first construct a candidate field ${{\varvec{F}}}$ by integrating the Cauchy flux along hyperplanes. This will be done in Sect. 8.1, where the constructed field is shown to satisfy $-\operatorname {div}{{\varvec{F}}}= \sigma$, thereby establishing *global recovery* of the flux. Although the construction of ${{\varvec{F}}}$ involves a choice of measure to integrate the flux, in Sect. 8.2, we will justify our choice by showing that the said flux is uniquely determined; this will rely on a version of Theorem 4.1 valid for half-spaces. Finally, we will show that the flux and the normal trace coincide in the sense of (6.19), which will be a *local recovery* result, in Sect. 9. This will first be done on generic cubes, after which an approximation argument will extend this to general surfaces. Once the main theorem is proven, we will also collect a few consequences in Sect. 9.2.

## Cauchy Flux II: Properties of the Cauchy Flux

### General Properties of the Cauchy Flux

We now present some useful properties of the Cauchy flux, starting with some properties of sets in ${\mathcal {O}}_{\mu }$ and consequences of property (iii). Recall that we have defined the class of open subsets:

$$\begin{aligned} {\mathcal {O}}_{\mu } = \Big \{ U \Subset \Omega \text { open}\, :\, \liminf _{\varepsilon \rightarrow 0} \frac{1}{\varepsilon } \,\mu (U \setminus {\overline{U}}^{\varepsilon }) < \infty \Big \}. \end{aligned}$$

In what follows, we also consider the more restrictive class:

$$\begin{aligned} \widetilde{{\mathcal {O}}}_{\mu } = \Big \{ U \Subset \Omega \text { open}\,:\,\limsup _{\varepsilon \rightarrow 0} \frac{1}{\varepsilon } \,\mu (U \setminus {\overline{U}}^{\varepsilon }) < \infty \Big \}. \end{aligned}$$

#### Lemma 7.1

If $U \in {\mathcal {O}}_{\mu },$ then ${\mathcal {F}}_{U}(\cdot )$ is represented by a measure supported on $\partial U$.

#### Proof

  By Condition (i), the flux is finitely additive. By (iii), there exists some measure $\mu ^{n-1}_{U}$ such that the following upper bound holds:

$$\begin{aligned} |{\mathcal {F}}_{U}(S)|\le \mu ^{n-1}_{U}(S) \quad \text{ for } \text{ any } \text{ Borel } \text{ set } S \subset \partial U. \end{aligned}$$

Note that setting $S = \varnothing$ gives ${\mathcal {F}}_{U}(\varnothing ) = 0$. For countable additivity, let $\{S_k\}_{k=1}^{\infty }$ be disjoint Borel subsets of $\partial U$, and set $S = \bigcup _{k=1}^{\infty } S_k$. Then, for each $N \ge 1$, we can estimate

$$\begin{aligned} \begin{aligned} \Big |{\mathcal {F}}_{U}(S) - \sum _{k=1}^N {\mathcal {F}}_{U}(S_k)\Big |&= \Big |{\mathcal {F}}_{U}( \bigcup _{k=N+1}^{\infty } S_k ) \Big |\\&\le \mu ^{n-1}_{U}( \bigcup _{k=N+1}^{\infty } S_k) = \sum _{k=N+1}^{\infty } \mu ^{n-1}_{U}(S_k) \rightarrow 0 \quad \text{ as } N \rightarrow \infty , \end{aligned} \end{aligned}$$

where we have repeatedly used the additivity property (i), in addition to (iii) and the fact that $\mu _U^{n-1}$ is a measure. Thus, it follows that

$$\begin{aligned} {\mathcal {F}}_{U}(S) = \sum _{k=1}^{\infty } {\mathcal {F}}_{U}(S_k), \end{aligned}$$

from which the result follows. $\square$

#### Lemma 7.2

Suppose that $U_1 \in {\mathcal {O}}_{\mu }$ and $U_2 \in \widetilde{{\mathcal {O}}}_{\mu }$. Then $U_1 \cap U_2$ and $U_1 \cup U_2$ lie in ${\mathcal {O}}_{\mu }$. In addition, if $U_1 \in \widetilde{{\mathcal {O}}}_{\mu }$, then $U_1 \cap U_2,\, U_1 \cup U_2 \in \widetilde{{\mathcal {O}}}_{\mu }$.

#### Proof

  For any $U_1, U_2 \subset \Omega$, set $U = U_1 \cap U_2$ and $V = U_1 \cup U_2.$ Then we claim:

7.1 $$\begin{aligned} U\setminus \overline{U^{\varepsilon }}&\subset (U_1 \setminus \overline{U_1^{\varepsilon }}) \cup (U_2 \setminus \overline{U_2^{\varepsilon }}), \end{aligned}$$

7.2 $$\begin{aligned} V\setminus \overline{V^{\varepsilon }}&\subset (U_1 \setminus \overline{U_1^{\varepsilon }}) \cup (U_2 \setminus \overline{U_2^{\varepsilon }}). \end{aligned}$$

Indeed, if $x \in U \setminus \overline{U^{\varepsilon }}$, then there exists $y \in {\mathbb {R}}^n \setminus U$ such that $|x - y |< \varepsilon$. Since ${\mathbb {R}}^n \setminus U = ({\mathbb {R}}^n \setminus U_1) \cup ({\mathbb {R}}^n \setminus U_2)$, then $x \in U_1 \setminus \overline{U_1^{\varepsilon }}$ if $y \in {\mathbb {R}}^n \setminus U_1$, and $x \in U_2 \setminus \overline{U_2^{\varepsilon }}$ if $y \in {\mathbb {R}}^n \setminus U_2$. Similarly, if $x \in V \setminus V^{\varepsilon }$, there is $y \in {\mathbb {R}}^n \setminus V$ with $|x- y|< \varepsilon$. If $x \in U_1$, then $y \in {\mathbb {R}}^n {\setminus } U_1$ so that $x \in U_1 \setminus \overline{U_1^{\varepsilon }}$; similarly, if $x \in U_2$, then $x \in U_2 {\setminus } \overline{U_2^{\varepsilon }}$.

Thus, taking $\mu$ on both sides of (7.1), we obtain

7.3 $$\begin{aligned} \frac{1}{\varepsilon } \, \mu (U \setminus \overline{U^{\varepsilon }}) \le \frac{1}{\varepsilon } \, \mu (U_1 \setminus \overline{U_1^{\varepsilon }}) + \frac{1}{\varepsilon } \, \mu (U_2 \setminus \overline{U_2^{\varepsilon }})\quad \text{ for } \text{ any } \varepsilon >0, \end{aligned}$$

so that

$$\begin{aligned} \liminf _{\varepsilon \rightarrow 0} \frac{1}{\varepsilon } \, \mu (U \setminus \overline{U^{\varepsilon }}) \le \liminf _{\varepsilon \rightarrow 0} \frac{1}{\varepsilon } \, \mu (U_1 \setminus \overline{U_1^{\varepsilon }}) + \limsup _{\varepsilon \rightarrow 0} \frac{1}{\varepsilon } \, \mu (U_2 \setminus \overline{U_2^{\varepsilon }}) < \infty , \end{aligned}$$

which implies that $U \in {\mathcal {O}}_{\mu }$. Moreover, if $U_1 \in \widetilde{{\mathcal {O}}}_{\mu }$, we have

$$\begin{aligned} \limsup _{\varepsilon \rightarrow 0} \frac{1}{\varepsilon } \, \mu (U \setminus \overline{U^{\varepsilon }}) \le \limsup _{\varepsilon \rightarrow 0} \frac{1}{\varepsilon } \, \mu (U_1 \setminus \overline{U_1^{\varepsilon }}) + \limsup _{\varepsilon \rightarrow 0} \frac{1}{\varepsilon } \, \mu (U_2 \setminus \overline{U_2^{\varepsilon }}) < \infty , \end{aligned}$$

so $U \in \widetilde{{\mathcal {O}}}_{\mu }$. The same result holds for *V* by using (7.2) instead to obtain an estimate analogous to (7.3). $\square$

Adapting the arguments of the above proof allows us to establish the following upper bound for the limit measure $\mu _{\partial U}^{n-1}$:

#### Lemma 7.3

Let $U_1, \cdots , U_N \in {\mathcal {O}}_{\mu }$ such that there exists a common subsequence $\varepsilon _k \rightarrow 0$ so that
and set

$$\begin{aligned} U = \bigcap _{i=1}^N U_i, \quad V = \bigcup _{i=1}^N U_i. \end{aligned}$$

(i) If  then
  $$\begin{aligned} \mu ^{n-1}_{U}(S) \le \sum _{i=1}^N \mu ^{n-1}_{U_i}(S \cap \partial U_i \cap \bigcap _{j \ne i} \overline{U_j}) \quad \text{ for } \text{ any } \text{ Borel } \text{ set } S \subset \partial U. \end{aligned}$$
(ii) If , then
  $$\begin{aligned} \mu _V^{n-1}(S) \le \sum _{i=1}^N \mu _{U_i}^{n-1}( S \cap (\partial U_i \setminus \bigcup _{j \ne i} U_j)) \quad \text{ for } \text{ any } \text{ Borel } \text{ set } S \subset \partial V. \end{aligned}$$

#### Proof

  For (i), arguing as in (7.1) from the proof of Lemma 7.2, we have

$$\begin{aligned} U \setminus \overline{U^\varepsilon } \subset \bigcup _{i=1}^N ( U_i \setminus \overline{U_i^{\varepsilon }} ) \quad \text{ for } \varepsilon >0. \end{aligned}$$

Given any non-negative $\phi \in C_{\textrm{c}}(\Omega )$, we can integrate $\phi$ over these levels sets with respect to $\mu$ and take the limit as $\varepsilon _k \rightarrow 0$ to obtain

$$\begin{aligned} \int _{\partial U} \phi \,\textrm{d}\mu ^{n-1}_{U} \le \sum _{i=1}^N \int _{\partial U_i} \phi \,\textrm{d}\mu ^{n-1}_{U_i}. \end{aligned}$$

Then we can argue by density to infer that

$$\begin{aligned} \mu _U^{n-1}(S) \le \sum _{i=1}^N \mu _{U_i}^{n-1}(S \cap \partial U_i) \quad \text{ for } \text{ any } \text{ Borel } \text{ set } S\subset \Omega . \end{aligned}$$

Now, since $\mu ^{n-1}_{\partial U}$ is supported on $\partial U$, we can replace *S* by $S \cap \partial U$ on the right-hand side. Moreover, since

$$\begin{aligned} \partial U_i \cap \partial U \subset \partial U_i \cap \bigcap _{j \ne i} \overline{U_j}, \end{aligned}$$

the result follows. The argument for (ii) is analogous, based on the inclusions:

$$\begin{aligned} V \setminus \overline{V^{\varepsilon }} \subset \bigcup _{i=1}^N (U_1 \setminus \overline{U_1^{\varepsilon }}) \end{aligned}$$

for each $\varepsilon >0$ which is established analogously to (7.2) and

$$\begin{aligned} \partial V \subset \bigcup _{i=1}^N \partial U_i \setminus \bigcup _{j \ne i} U_j. \end{aligned}$$

$$\square $$

#### Lemma 7.4

Let ${\mathcal {F}}$ be a Cauchy flux, and let $U \subset {\mathbb {R}}^n$ be an open set. Consider the disintegration of $\mu$ along $\partial U^t$ given by
from Lemma 3.5, extending $\mu$ by zero to a measure on ${\mathbb {R}}^n$. Then, for ${\mathcal {L}}^1$–*a.e.* $t>0$,
7.4and $\mu (\Omega \cap \partial U^t) = 0$. In particular, if $U \Subset \Omega$, then $U^t, ({\overline{U}}^t)^{\textrm{c}} \in {\mathcal {O}}_{\mu }$, and $\mu ^{n-1}_{U^t}$ and $\mu ^{n-1}_{({\overline{U}}^t)^{\textrm{c}}}$ are uniquely determined as $\mu _t$ for ${\mathcal {L}}^{1}$–*a.e.* $t>0$.

#### Proof

  We apply Lemma 3.5, which asserts that, for any $\phi \in C_{\textrm{c}}(\Omega )$ and any $0<t<s$,

$$\begin{aligned} \frac{1}{s-t} \int _{U^t \setminus \overline{U^{s}}} \phi \,\textrm{d}\mu = \frac{1}{s-t} \int _t^{s} \int _{\partial U^r} \phi \,\textrm{d}\mu _r \,\textrm{d}r + \frac{1}{s-t}\int _{(t,s)} \int _{\partial U^r} \phi \,\textrm{d}{\tilde{\mu }}_r \,\textrm{d}\tau _{\textrm{sing}}(r). \end{aligned}$$

For $\varepsilon > 0$, we apply the above with $s=t+\varepsilon$. For any countable dense subset $\{\phi _j\}$ of $C_{\textrm{c}}(\Omega )$ (with respect to the uniform convergence), consider the partial mappings:

$$\begin{aligned} \Phi _j(t)&:= \int _{\partial U^t} \phi _j \,\textrm{d}\mu _t \quad \text {for each } j. \end{aligned}$$

Then we choose $t>0$ to be a Lebesgue point for each $\Phi _j$ and to satisfy $D_{{\mathcal {L}}^1}(\tau _{\textrm{sing}})(t) = 0$ (using Lemma 2.1), which forms a set whose complement is ${\mathcal {L}}^1$–null. For such *t*, sending $\varepsilon \rightarrow 0$ gives that, for each *j*,

$$\begin{aligned} \lim _{\varepsilon \rightarrow 0} \frac{1}{\varepsilon } \int _t^{t+\varepsilon } \int _{\partial U^s} \phi _j \,\textrm{d}\mu _s \,\textrm{d}s = \Phi (t) = \int _{\partial U^t} \phi _j \,\textrm{d}\mu _t, \end{aligned}$$

and

$$\begin{aligned}  &   \limsup _{\varepsilon \rightarrow 0} \frac{1}{\varepsilon } \Big |\int _{(t,t+\varepsilon )} \int _{\partial U^t} \phi _j \,\textrm{d}{\tilde{\mu }}_s\,\textrm{d}\tau _{\textrm{sing}}(s)\Big |\\  &   \quad \le \Vert \phi _j \Vert _{L^{\infty }(\Omega )} \limsup _{\varepsilon \rightarrow 0} \frac{1}{\varepsilon } \, |\tau _{\textrm{sing}}((t,t+\varepsilon ))|= 0. \end{aligned}$$

Combining the above, we deduce

7.5 $$\begin{aligned} \lim _{\varepsilon \rightarrow 0} \frac{1}{\varepsilon } \int _{U^t \setminus {{\overline{U}}}^{t+\varepsilon }} \phi _j\,\textrm{d}\mu = \int _{\partial U^t} \phi _j \,\textrm{d}\mu _t. \end{aligned}$$

Applying the same to $(t,s) = (t-\varepsilon ,t)$ with $0<\varepsilon <t$, we have

$$\begin{aligned} \lim _{\varepsilon \rightarrow 0} \frac{1}{\varepsilon } \int _{U^{t-\varepsilon } \setminus \overline{U^t}} \phi _j \,\textrm{d}\mu = \int _{\partial U^t} \phi _j \,\textrm{d}\mu _t \quad \text{ for } \text{ the } \text{ same } t. \end{aligned}$$

Discarding a null set if necessary, we can moreover assume that

$$\begin{aligned} \limsup _{\varepsilon \rightarrow 0}\frac{1}{2\varepsilon } \, \mu (U^{t-\varepsilon } \setminus {{\overline{U}}}^{t+\varepsilon }) < \infty , \end{aligned}$$

so that, in particular, $U^t, ({\overline{U}}^t)^{\textrm{c}} \in \widetilde{{\mathcal {O}}}_{\mu }$ and $\mu (\Omega \cap \partial U^t) = 0$. Then, for any sequence $\varepsilon _k \rightarrow 0$ giving rise to a limiting measure
testing against $\phi _j$ and using (7.5) yields

$$\begin{aligned} \int _{\Omega } \phi _j \,\textrm{d}\nu = \int _{\partial U^t} \phi _j \,\textrm{d}\mu _t \quad \text{ for } \text{ any } j. \end{aligned}$$

By density of $\{\phi _j\}$, it follows that . This uniquely determines the limit. We can similarly show that
as required. $$\square $$

#### Remark 7.5

  In the theory of $L^p$–integrable fields, one may consider $\mu = h \,{\mathcal {L}}^n$ with a non-negative function $h \in L^p(\Omega )$. In this case, the disintegration reads as
by using the coarea formula, where a representative of *h* is fixed. In this case, using Lemma 7.4, condition (iii) implies that

$$\begin{aligned} |{\mathcal {F}}_{ U^t}(S) |\le \int _{S} h \,\textrm{d}{\mathcal {H}}^{n-1} \quad \text{ for } {\mathcal {L}}^1\textit{--a.e.}\,\,t>0 \text{ and } \text{ any } \text{ Borel } \text{ set } S \subset \partial U^t. \end{aligned}$$

This is reminiscent of the flux bound appearing in [9, 31, 59, 61], even though the notions need not coincide for a general open set *U*.

For the next result, we introduce the sets

7.6 $$\begin{aligned} {{\widetilde{\Omega }}}^{\delta } = B_{1/\delta } \cap \Omega ^{\delta } = \Big \{ x \in \Omega \,:\,\,\textrm{dist}(x,\partial \Omega )>\delta , \ |x |< \frac{1}{\delta } \Big \} \quad \text{ for } \text{ each } \delta >0. \end{aligned}$$

Note that each ${{\widetilde{\Omega }}}^{\delta } \Subset \Omega$ and $\bigcup _{\delta >0} {{\widetilde{\Omega }}}^{\delta } = \Omega$. Using Lemmas 7.2 and 7.4, we see that ${{\widetilde{\Omega }}}^{\delta } \in \widetilde{{\mathcal {O}}}_{\mu }$ for ${\mathcal {L}}^1$–*a.e.* $\delta >0$.

#### Lemma 7.6

Suppose that $U \subset {\mathbb {R}}^n$ is an open set such that
7.7Then an extension of the Cauchy flux can be defined by

7.8 $$\begin{aligned} {\mathcal {F}}_{U}(S) := {\mathcal {F}}_{U \cap {{\widetilde{\Omega }}}^{\delta }}(S) \end{aligned}$$

for ${\mathcal {L}}^1$–*a.e.* $\delta >0$ such that ${{\widetilde{\Omega }}}^{\delta } \in \widetilde{{\mathcal {O}}}_{\mu }$ and for any Borel set $S \subset \partial U$ such that $S \subset {{\widetilde{\Omega }}}^{\delta } \cap \partial U$. Moreover, this extends uniquely to a measure on $\Omega \cap \partial U$ satisfying

7.9 $$\begin{aligned} |{\mathcal {F}}_{U}(S)|\le \mu ^{n-1}_{U}(S) \quad \text {for any Borel set } S \subset \Omega \cap \partial U. \end{aligned}$$

#### Proof

  First, the weak ${}^{*}$–convergence in (7.7) implies that

$$\begin{aligned} \limsup _{\varepsilon \rightarrow 0} \frac{1}{\varepsilon } \, \mu (\Omega \cap (U \setminus \overline{U^{\varepsilon }})) < \infty . \end{aligned}$$

By Lemma 7.2 applied in ${\mathbb {R}}^n$ via extending $\mu$ by zero outside $\Omega$, if $\delta >0$ such that ${{\widetilde{\Omega }}}^{\delta } \in \widetilde{{\mathcal {O}}}_{\mu }$, then ${{\widetilde{\Omega }}}^{\delta } \cap U \in \widetilde{{\mathcal {O}}}$. By property (ii), we see that

$$\begin{aligned} {\mathcal {F}}_{U \cap {{\widetilde{\Omega }}}^{\delta _1}}(A \cap \partial U) = {\mathcal {F}}_{U \cap {{\widetilde{\Omega }}}^{\delta _2}}(A \cap \partial U) \end{aligned}$$

for any $\delta _1$ and $\delta _2$ such that $0< \delta _1 < \delta _2$, as above, and $A \subset {{\widetilde{\Omega }}}_{\delta _2}$ open. Since the flux is a measure on ${\mathcal {O}}_{\mu }$ by Lemma 7.1, by approximation (Lemma 2.2), the above extends to general Borel sets $S \subset {{\widetilde{\Omega }}}^{\delta _2} \cap \partial U$. Hence, (7.8) is well-defined. To show that it extends to a measure, using Lemma 7.4, let $\delta >0$ such that
Then, since $U \cap {{\widetilde{\Omega }}}^{\delta } \in {\mathcal {O}}_{\mu }$ by Lemma 7.3, we can estimate
as measures. Then, if $S \subset \Omega \cap \partial U$ such that $S \Subset {{\widetilde{\Omega }}}^{\delta }$, we obtain the claimed bound

$$\begin{aligned} |{\mathcal {F}}_{U}(S) |\le \mu ^{n-1}_{U}(S). \end{aligned}$$

Since this estimate is independent of $\delta >0$, we claim that ${\mathcal {F}}_U$ extends uniquely to a measure on $\Omega \cap \partial U$. Indeed, take $\delta _j \searrow 0$ such that each ${{\widetilde{\Omega }}}^{\delta _j} \in \widetilde{{\mathcal {O}}}_{\mu }$ and, given $S \subset \Omega \cap \partial U$, define $S_1 = S \cap {{\widetilde{\Omega }}}^{\delta _1}$ and $S_j = S \cap ({{\widetilde{\Omega }}}^{\delta _j} {\setminus } \overline{{{\widetilde{\Omega }}}^{\delta _{j-1}}})$ for $j \ge 2$. Then, by additivity, we have

$$\begin{aligned} \sum _{j=1}^N {\mathcal {F}}_U(S_j) = {\mathcal {F}}_U( \bigcup _{j=1}^N S_j), \end{aligned}$$

which is uniformly bounded in *N* by noting that

$$\begin{aligned} \sum _{j=1}^{\infty }|{\mathcal {F}}_U(S_j)|\le \mu _U^{n-1}(S) < \infty . \end{aligned}$$

Thus, there is a unique limit

$$\begin{aligned} {\mathcal {F}}_U(S) := \sum _{j=1}^{\infty } {\mathcal {F}}_{U}(S_j), \end{aligned}$$

which also satisfies (7.9). Arguing analogously with disjoint sets $S_1, S_2\subset \partial U$ and considering $S_{i,j} = S_i \cap ({{\widetilde{\Omega }}}^{\delta _j} \setminus \overline{{{\widetilde{\Omega }}}^{\delta _{j-1}}})$, we see that this extension is also additive. Since this extension is additive and satisfies (7.9), arguing as in the proof of Lemma 7.1, we conclude that this extension ${\mathcal {F}}_U$ is a measure on $\Omega \cap \partial U$. $\square$

#### Remark 7.7

  In the case that $U = {\overline{V}}^{\textrm{c}} = {\mathbb {R}}^n \setminus {{{\overline{V}}}}$ for some $V \Subset \Omega$, it suffices to assume that

$$\begin{aligned} \liminf _{\varepsilon \rightarrow 0} \frac{1}{\varepsilon } \,\mu (\Omega \cap (V^{-\varepsilon } \setminus {\overline{V}})) < \infty , \end{aligned}$$

which is written as ${\overline{V}}^{\textrm{c}} \in {\mathcal {O}}_{\mu }$. In this case, we can set
7.10for any $\delta >0$ such that $\delta < \textrm{dist}(V,\partial \Omega )$, $V \subset B_{1/\delta }$, and ${{\widetilde{\Omega }}}^{\delta } \in \widetilde{{\mathcal {O}}}_{\mu }$. Indeed, by Lemma 7.2 (applied in ${\mathbb {R}}^n$ extending $\mu$ by zero), ${{\widetilde{\Omega }}}^{\delta } {\setminus } {{\overline{V}}} \in \mathcal O_{\mu }$, so it follows from Lemma 7.1 that ${\mathcal {F}}_{{{\widetilde{\Omega }}}^{\delta } \setminus {{\overline{V}}}}$ is a measure. By the localization property (ii), its restriction to $\partial V$ is independent of the choice of $\delta >0$, so (7.10) is well-defined. Also, for any such $\delta$, we can set , which satisfies

$$\begin{aligned} |{\mathcal {F}}_{{\overline{V}}^{\textrm{c}}}(S) |\le \mu _{{\overline{V}}^{\textrm{c}}}^{n-1}(S) \quad \text {for any Borel set } S \subset \partial V. \end{aligned}$$

###  Properties of the Cauchy Flux on Cubes and Half-Spaces

Our proof of Theorem 6.6 relies on considering the Cauchy flux ${\mathcal {F}}$ on the boundaries of half-spaces and cubes, and integrating along suitable hyperplanes to construct the desired field ${{\varvec{F}}}$. Since a general half-space $H = \{ x \in {\mathbb {R}}^n\,:\, a \cdot x > t\}$ need not be contained in $\Omega$, we use Lemma 7.6 to consider the flux on $\Omega \cap \partial H$ for *almost all* *H*. In doing so, it is useful to understand the behavior of the flux on cubes and, in particular, write ${\mathcal {F}}_{Q}(\cdot )$ as a sum of fluxes going through the respective faces. It turns out that this is not possible for arbitrary cubes, since the vector field may potentially concentrate at the corners, as illustrated for the normal traces in Example 5.1.

However, localization to faces is possible for *almost all* cubes, which we will make precise below. For this, it is useful to introduce some notation. For simplicity, we consider only cubes and hyperplanes subordinate to the coordinate axes $x_1, \cdots , x_n$, even though more general frames in ${\mathbb {R}}^n$ can also be considered as done in [31] with minimal modifications.

#### Definition 7.8

  For a cube $Q = Q(a,b):= (a_1,b_1) \times \cdots \times (a_n,b_n)$, define the 2-skeleton of *Q* as

$$\begin{aligned} \partial ^2Q = \big \{ x \in {{\overline{Q}}}\,:\,x_i \in \{a_i,b_i\}, x_j \in \{ a_j,b_j\} \text { for some } i \ne j \big \}. \end{aligned}$$

Note that, in two dimensions, $\partial ^2Q$ consists of the corner points, whereas it is the union of all of the edges of *Q* (including the vertices) in three dimensions.

Additionally, for $1 \le i \le n$, define the half-spaces

$$\begin{aligned} H_{i, +}^t = \big \{ x \in {\mathbb {R}}^n\,:\,x_i > t\big \}, \quad H_{i, -}^t = \big \{ x \in {\mathbb {R}}^n\,:\,x_i < t\big \}. \end{aligned}$$

Note that $(H_{i,+}^t)^{\varepsilon } = H_{i,+}^{t+\varepsilon }$ and $(H_{i,-}^t)^{\varepsilon } = H_{i,-}^{t-\varepsilon }$ by recalling definition (6.15). In particular, $\partial H_{i,+}^{a_i}$ and $\partial H_{i,-}^{b_i}$ contain the faces of *Q* for each $1 \le i \le n$.

We also define suitable null sets to make precise our notions of *almost all* cubes and half-spaces.

#### Definition 7.9

  Given the Radon measures $\mu$ and $\sigma$ from Definition 6.4, for $1 \le i \le n$, define ${\mathcal {N}}_{i,\mu } \subset {\mathbb {R}}$ to be the complement of the set of points $t \in {\mathbb {R}}$ for which $\mu (\partial H_{i,+}^t )=0$, and the limits:
exist as $\varepsilon \rightarrow 0$ with $\mu _{H_{i,+}^{t}}^{n-1} = \mu _{H_{i,-}^{t}}^{n-1}$, where $\mu$ is viewed as a measure on ${\mathbb {R}}^n$ by setting $|\mu |({\mathbb {R}}^n \setminus \Omega ) = 0$, and the set ${\mathcal {N}}_{i,\mu } \subset {\mathbb {R}}$ is ${\mathcal {L}}^1$-null by Lemma 7.4. Furthermore, define ${\mathcal {M}}_{i,\sigma } \subset {\mathbb {R}}$ as the complement of the set of points $t \in {\mathbb {R}}$ such that

$$\begin{aligned} |\sigma |( \partial H_{i,+}^t) = 0, \end{aligned}$$

which is at most countable and hence ${\mathcal {L}}^1$-null.

Observe that Lemma 7.6 applies to the half-spaces $H_{i,\pm }^t$, provided that $t \notin {\mathcal {N}}_{i,\mu }$, allowing us to make sense of ${\mathcal {F}}_{ H_{i,\pm }^t}$, which will frequently be used in what follows.

#### Lemma 7.10

Let $Q = Q(a,b) \Subset \Omega$ be a cube such that $a_i,b_i \notin {\mathcal {N}}_{i,\mu }$ and

7.11 $$\begin{aligned} \mu _{H_{i,+}^{a_i}}^{n-1}(\partial ^2Q) = \mu _{H_{i,-}^{b_i}}^{n-1}(\partial ^2Q) = 0 \quad \text{ for } \text{ any } 1 \le i \le n. \end{aligned}$$

Then $Q, {{\overline{Q}}}^{\textrm{c}} \in \widetilde{{\mathcal {O}}}_{\mu }$, and there is a unique measure $\mu _Q^{n-1}$ such that
7.12Furthermore, for each $$1 \le i \le n$$,
which uniquely determines $\mu _Q^{n-1}$. In particular, $\mu _Q^{n-1}(\partial ^2Q) = \mu _{\,{{\overline{Q}}}^{\mathrm c}}^{n-1}(\partial ^2Q) = 0$.

#### Proof

  Viewing $\mu$ as a measure on ${\mathbb {R}}^n$, we see that each $H_{i,+}^{a_i}, H_{i,-}^{b_i} \in \widetilde{{\mathcal {O}}}_{\mu }$. Then it follows from Lemma 7.2 that $Q \in \widetilde{{\mathcal {O}}}_{\mu }$. Now, for any $\varepsilon _k \searrow 0$, there exists a limit measure $\lambda$ such that
We now show this limit measure is uniquely determined, from which the full convergence (7.12) follows.

Since $Q = \bigcap _{i=1}^n (H_{i,+}^{a_i} \cap H_{i,-}^{b_i})$ by using Lemma 7.3(i) together with (7.11), we have

7.13 $$\begin{aligned} \lambda (\partial ^2 Q) = 0. \end{aligned}$$

Moreover, if $\phi \in C_{\textrm{c}}(\Omega \setminus \partial ^2Q)$, then $\phi$ is supported in some open subset $A \Subset \Omega \setminus \partial ^2Q$. Choosing $\varepsilon >0$ such that $\varepsilon < \textrm{dist}(A,\partial ^2Q)$ and $\varepsilon < \frac{1}{2}(b_j - a_j)$ for each $1 \le j \le n$, we have

$$\begin{aligned} A \cap (Q \setminus \overline{Q^{\varepsilon }}) = \bigcup _{j=1}^n \Big (A \cap (H_{j,+}^{a_j} \setminus \overline{H_{j,+}^{a_j+\varepsilon }})\Big ) \cup \bigcup _{j=1}^n \Big (A \cap (H_{j,-}^{b_j} \setminus \overline{H_{j,-}^{b_j-\varepsilon }})\Big ), \end{aligned}$$

and the sets of the right-hand side are disjoint. Hence, integrating over $\phi$ with respect to $\frac{1}{\varepsilon } \,\mu$ and sending $\varepsilon _k \searrow 0$ give

$$\begin{aligned} \int _{\partial Q} \phi \,\textrm{d}\lambda = \sum _{j=1}^n \int _{\partial H_{j,+}^{a_j}} \phi \,\textrm{d}\mu _{H_{j,+}^{a_j}}^{n-1} +\sum _{j=1}^n \int _{\partial H_{j,-}^{b_j}} \phi \,\textrm{d}\mu _{H_{j,-}^{b_j}}^{n-1}. \end{aligned}$$

This, combined with (7.13), uniquely determines $\lambda = \mu _{ Q}^{n-1}$. Similarly, for the complement, we obtain that ${{\overline{Q}}}^{\textrm{c}} \in \widetilde{{\mathcal {O}}}_{\mu }$ by using Lemma 7.2 and noting that it is the union of $H_{i,-}^{a_i}$ and $H_{i,+}^{b_i}$ for each $1 \le i \le n$. Then, if $\varepsilon _k \searrow 0$ for which
it follows from Lemma 7.3(b) that ${{\tilde{\lambda }}}(\partial ^2Q) = 0$. Moreover, we argue that, for each $A \Subset \Omega \setminus \partial ^2Q$,
from which we infer that ${{\tilde{\lambda }}} = \mu ^{n-1}_Q$, since $\mu _{\partial H_{j,-}^{a_j}}^{n-1} = \mu _{\partial H_{j,+}^{a_j}}^{n-1}$ and $\mu _{\partial H_{j,+}^{b_j}}^{n-1} = \mu _{\partial H_{j,-}^{b_j}}^{n-1}$ for each $1 \le j \le n$. $\square$

#### Lemma 7.11

Let $Q = Q(a,b) \Subset \Omega$ be a cube such that $a_i,b_i \notin {\mathcal {N}}_{i,\mu }$ for each $1 \le i \le n$ and that (7.11) holds. Then, for any Borel set $S \subset \partial Q$,

7.14 $$\begin{aligned}&{\mathcal {F}}_{Q}(S) = \sum _{i=1}^n \Big ( {\mathcal {F}}_{H_{i,+}^{a_i}}(S \cap \partial H_{i,+}^{a_i}) + {\mathcal {F}}_{H_{i,-}^{b_i}}(S \cap \partial H_{i,-}^{b_i}) \Big ), \end{aligned}$$

7.15 $$\begin{aligned}&{\mathcal {F}}_{\,{{\overline{Q}}}^{\textrm{c}}}(S) = \sum _{i=1}^n \Big ( {\mathcal {F}}_{H_{i,-}^{a_i}}(S \cap \partial H_{i,-}^{a_i}) + {\mathcal {F}}_{H_{i,+}^{b_i}}(S \cap \partial H_{i,+}^{b_i})\Big ). \end{aligned}$$

#### Proof

  By Lemma 7.6, the flux is defined on the hyperplanes associated with each face of *Q*. Also, by Lemma 7.10 and condition (iii), $Q, {{\overline{Q}}}^{\textrm{c}} \in \widetilde{{\mathcal {O}}}_{\mu }$ so that ${\mathcal {F}}_Q$ and ${\mathcal {F}}_{\,{{\overline{Q}}}^{\textrm{c}}}$ are measures majorized by $\mu _Q^{n-1}$ (by using Remark 7.7 for the complement).

Since $\Omega \setminus \partial ^2Q$ is open, for any open set $A \subset \Omega \setminus \partial ^2Q$, the additivity property (i) from Definition 6.4 gives

$$\begin{aligned} \begin{aligned} {\mathcal {F}}_{Q}(A \cap \partial Q)&= \sum _{i=1}^n \Big ( {\mathcal {F}}_{ Q}(A \cap \partial Q\cap \partial H_{i,+}^{a_i}) + {\mathcal {F}}_{Q}(A \cap \partial Q\cap \partial H_{i,-}^{b_i}) \Big ), \end{aligned} \end{aligned}$$

by noting that the collection $\{(\partial H_{i,+}^{a_i}, \partial H_{i,-}^{b_i}):\,1 \le i \le n\}$ is pairwise disjoint in ${\mathbb {R}}^n \setminus \partial ^2Q$.

We now claim that, for each $1 \le i \le n$,

$$\begin{aligned} {\mathcal {F}}_{Q}(A \cap \partial Q \cap \partial H_{i,+}^{a_i})&= {\mathcal {F}}_{ H_{i,+}^{a_i}}(A \cap \partial Q\cap \partial H_{i,+}^{a_i}), \\ {\mathcal {F}}_{Q}(A \cap \partial Q\cap \partial H_{i,-}^{b_i})&= {\mathcal {F}}_{H_{i,-}^{b_i}}(A \cap \partial Q\cap \partial H_{i,-}^{b_i}). \end{aligned}$$

Indeed, when $i=1$, writing $Q = (a_1,b_1) \times Q'$ with $Q' \subset {\mathbb {R}}^{n-1}$, for $\delta >0$ sufficiently small, we see that $Q_{1,\delta } = (a_1-\delta ,a_1+\delta ) \times Q'$ is an open cube such that

$$\begin{aligned} A \cap Q_{1,\delta } \cap Q = A \cap Q_{1,\delta } \cap H_{1,+}^{a_1}. \end{aligned}$$

Thus, applying property (ii) with the open set $A \cap Q_{1,\delta }$, we have

$$\begin{aligned} \begin{aligned} {\mathcal {F}}_{Q}(A \cap \partial Q \cap \partial H_{1,+}^{a_1})&= {\mathcal {F}}_{Q}(A \cap Q_{1,\delta } \cap \partial Q) \\&= {\mathcal {F}}_{ H_{1,+}^{a_1}}(A \cap Q_{1,\delta } \cap \partial H_{1,+}^{a_1}) = {\mathcal {F}}_{ H_{1,+}^{a_1}}(A \cap \partial Q \cap \partial H_{1,+}^{a_1}). \end{aligned} \end{aligned}$$

Arguing similarly for the remaining terms, the claim follows.

Therefore, we have

$$\begin{aligned} {\mathcal {F}}_{Q}(A \cap \partial Q) = \sum _{i=1}^n \Big ( {\mathcal {F}}_{ H_{i,+}^{a_i}}(A \cap \partial Q\cap \partial H_{i,+}^{a_i}) + {\mathcal {F}}_{H_{i,-}^{b_i}}(A\cap \partial Q \cap \partial H_{i,-}^{b_i}) \Big ) \end{aligned}$$

for any open set $A \subset \Omega \setminus \partial ^2Q$.

By Lemmas 7.1 and 7.6, we know that both sides of (7.14) are Radon measures on $\partial Q$, and the equality holds for any relatively open set $S \subset \partial Q \setminus \partial ^2Q$, so that this extends to all Borel subsets $S \subset \partial Q \setminus \partial ^2Q$ by Lemma 2.2. Moreover, since both sides of (7.14) vanish on $\partial ^2Q$ by property (iii), (7.11), and Lemma 7.10, this extends to hold for any Borel set $S \subset \partial Q$. The argument for the flux on the complement, namely (7.15), is analogous. $\square$

The previous lemma relies crucially on condition (7.11), which ensures that the flux does not concentrate on the corners of the cube. The following result asserts that this condition holds for *almost all* cubes in a suitable sense, which is used frequently in the sequel. We postpone the technical proof until the end of this section, as it is disjoint from the discussion which follows.

#### Lemma 7.12

Given a cube $Q=Q(a,b) \Subset \Omega$, for ${\mathcal {L}}^n$–*a.e.* $x \in {\mathbb {R}}^n$, (7.11) holds for $x+Q$:

$$\begin{aligned} \mu _{H_{i,+}^{x_i+a_i}}^{n-1}(\partial ^2(x+Q)) = \mu _{H_{i,-}^{x_i+b_i}}^{n-1}(\partial ^2(x+Q)) = 0 \quad \text {for any } 1 \le i \le n. \end{aligned}$$

#### Lemma 7.13

Consider a cube $Q = Q(a,b) \Subset \Omega$ with $a_i,b_i \notin {\mathcal {N}}_{i,\mu }$ which satisfies (7.11) for each $1 \le i \le n$. Then, for each $1 \le j \le n$, there exists an ${\mathcal {L}}^1$-null set ${\mathcal {N}}_{j,Q} \subset (a_j,b_j)$ such that

7.16 $$\begin{aligned} t \mapsto {\mathcal {F}}_{H_{j,+}^t}(Q \cap \partial H_{j,+}^t) \end{aligned}$$

is continuous when restricted to ${\mathbb {R}} \setminus {\mathcal {N}}_{j,Q}$ and hence is ${\mathcal {L}}^1$-measurable on ${\mathbb {R}}$, and

7.17 $$\begin{aligned} {\mathcal {F}}_{H_{j,+}^t}(Q \cap \partial H_{j,+}^t) = - {\mathcal {F}}_{H_{j,-}^{t}}(Q \cap \partial H_{j,-}^{t}) \quad \text {for any }t \in {\mathbb {R}} \setminus {\mathcal {N}}_{j,Q}. \end{aligned}$$

#### Proof

  We divide the proof into two steps.

**1**. By permuting the coordinates, we can assume that $j = 1$. We write $Q = (a_1,b_1) \times Q'$ and set $Q_{s,t} := (s,t) \times Q' \subset Q$ for $a_1< s< t < b_1$. This satisfies

$$\begin{aligned} \partial ^2 Q_{s,t} \subset \{s,t\} \times \partial Q' \cup \partial ^2Q, \end{aligned}$$

viewing $\partial Q'$ as the boundary of $Q' \subset {\mathbb {R}}^{n-1}$. Furthermore, notice that

$$\begin{aligned} \partial ^2 Q_{s,t} \cap (\partial H_{i,+}^{a_i} \cup \partial H_{i,-}^{b_i}) \subset \partial Q' \cup \partial ^2Q \quad \text {for any } 2 \le i \le n. \end{aligned}$$

combining this with (7.11), we obtain

7.18 $$\begin{aligned} \mu _{H_{i,+}^{a_i}}^{n-1}(\partial ^2 Q_{s,t}) = \mu _{H_{i,-}^{b_i}}^{n-1}(\partial ^2Q_{s,t}) = 0 \quad \text {for any } 2\le i \le n. \end{aligned}$$

Observe that, since $a_i,b_i \not \in {\mathcal {N}}_{i,\mu }$, we have

$$\begin{aligned} \mu ((a_1,b_1) \times \partial Q') = 0 \end{aligned}$$

owing to $\mu (\partial H_{i,+}^{a_i}) = \mu (\partial H_{i,-}^{b_i}) = 0$ for all $2 \le i \le n$ by Definition 7.9. Thus, for ${\mathcal {L}}^1$–*a.e.* $t \in (a_1,b_1)$,

$$\begin{aligned} \mu _{H_{i,+}^{t}}^{n-1}(((a_1,b_1) \times \partial Q') \cap \partial H_{i,+}^{t}) = \mu _{H_{i,-}^{t}}^{n-1}(((a_1,b_1) \times \partial Q') \cap \partial H_{i,-}^{t}) = 0. \end{aligned}$$

We denote by ${\mathcal {T}}$ the set of points $t \in (a_1,b_1) \setminus ({\mathcal {N}}_{1,\mu } \cup {\mathcal {M}}_{1,\sigma })$ for which the above holds. Then, for $s,t \in {\mathcal {T}}$ with $s<t$, we have

$$\begin{aligned} \mu _{H_{1,+}^{s}}^{n-1}(\partial ^2Q_{s,t}) = \mu _{H_{1,-}^{t}}^{n-1}(\partial ^2Q_{s,t}) = 0. \end{aligned}$$

Then, combined with (7.18), Lemmas 7.10–7.11 apply to $Q_{s,t}$.

**2**. We now show the result holds with ${\mathcal {N}}_{1,Q} = (a_1,b_1) {\setminus } {\mathcal {T}}$.

Let $\varepsilon _k,\delta _k>0$ such that $\varepsilon _k,\delta _k \searrow 0$ and $t - \varepsilon _k, t + \delta _k \in {\mathcal {T}}$ for each *k*. Then, applying Lemma 7.11 and the balance law (6.17) to cube $Q_{t-\varepsilon _k,t}$, we deduce

$$\begin{aligned} \begin{aligned} \sigma (Q_{t-\varepsilon _k,t})&= {\mathcal {F}}_{Q_{t-\varepsilon _k,t}}(\partial Q_{t-\varepsilon _k,t}) \\  &= {\mathcal {F}}_{H_{1,+}^{t-\varepsilon _k}}(Q \cap \partial H_{1,+}^{t-\varepsilon _k}) + {\mathcal {F}}_{H_{1,-}^{t}}(Q \cap \partial H_{1,-}^{t}) \\  &\quad \, + \sum _{i=2}^n \Big ( {\mathcal {F}}_{H_{i,+}^{a_i}}(Q_{t-\varepsilon _k,t} \cap \partial H_{i,+}^{a_i}) + {\mathcal {F}}_{H_{i,-}^{b_i}}(\partial Q_{t-\varepsilon _k,t} \cap \partial H_{i,-}^{b_i})\Big ) \\  &=: I_{1,+}^k + I_{1,-} + \sum _{i=2}^n \big ( I_{i,+}^k + I_{i,-}^k \big ). \end{aligned} \end{aligned}$$

By Lemma 7.1, the fluxes on the respective hyperplanes are measures. It follows that $I_{i,\pm }^k \rightarrow 0$ as $k \rightarrow \infty$ for each $2 \le i \le n$. Similarly, $\sigma (Q_{t-\varepsilon _k,t}) \rightarrow 0$. Then we see that

$$\begin{aligned} \lim _{k \rightarrow \infty } {\mathcal {F}}_{H_{1,+}^{t-\varepsilon _k}}(Q \cap \partial H_{1,+}^{t-\varepsilon _k}) = \lim _{k \rightarrow \infty } I_{1,+}^k = - I_{1,-} =- {\mathcal {F}}_{H_{1,-}^{t}}(Q \cap \partial H_{1,-}^{t}). \end{aligned}$$

Analogously, for $Q_{t,t+\delta _k}$, it follows that

$$\begin{aligned} \lim _{k \rightarrow \infty } {\mathcal {F}}_{H_{1,-}^{t+\delta _k}}(Q \cap \partial H_{1,-}^{t+\delta _k})=- {\mathcal {F}}_{H_{1,+}^{t}}(Q \cap \partial H_{1,+}^{t}), \end{aligned}$$

and, for $Q_{t-\varepsilon _k,t+\delta _k}$, we apply that $\sigma (\partial H_{1,+})=0$ to obtain

$$\begin{aligned} \lim _{k \rightarrow \infty } {\mathcal {F}}_{H_{1,-}^{t+\delta _k}}(Q \cap \partial H_{1,-}^{t+\delta _k}) + \lim _{k \rightarrow \infty } {\mathcal {F}}_{H_{1,+}^{t-\varepsilon _k}}(Q \cap \partial H_{1,+}^{t-\varepsilon _k}) = 0. \end{aligned}$$

Thus, it follows that

$$\begin{aligned} {\mathcal {F}}_{H_{1,+}^{t}}(Q \cap \partial H_{1,+}^{t}) = - {\mathcal {F}}_{H_{1,-}^{t}}(Q \cap \partial H_{1,-}^{t}) \end{aligned}$$

establishing (7.17), which implies

$$\begin{aligned} \lim _{k \rightarrow \infty } {\mathcal {F}}_{H_{1,-}^{t+\delta _k}}(Q \cap \partial H_{1,-}^{t+\delta _k})&= {\mathcal {F}}_{H_{1,-}^{t}}(Q \cap \partial H_{1,-}^{t}), \\ \lim _{k \rightarrow \infty } {\mathcal {F}}_{H_{1,+}^{t-\varepsilon _k}}(Q \cap \partial H_{1,+}^{t-\varepsilon _k})&= {\mathcal {F}}_{H_{1,+}^{t}}(Q \cap \partial H_{1,+}^{t}), \end{aligned}$$

leading to the continuity of (7.16) restricted to ${\mathbb {R}} \setminus {\mathcal {N}}_{j,Q}$. Finally, the ${\mathcal {L}}^1$–measurability follows from a general principle; indeed, denoting this mapping by *g*, if $A \subset {\mathbb {R}}$ is open, then

$$\begin{aligned} g^{-1}(A) = \big (g|_{{\mathbb {R}} \setminus {\mathcal {N}}_{j,Q}}\big )^{-1}(A) \cup \big (g^{-1}(A) \cap {\mathcal {N}}_{j,Q}\big ). \end{aligned}$$

This is the union of a relatively open subset of the ${\mathcal {L}}^1$-measurable set ${\mathbb {R}} \setminus N_{j,Q}$ and an ${\mathcal {L}}^1$-null set, so both are ${\mathcal {L}}^1$-measurable by using the completeness of the Lebesgue measure. Therefore, *g* is ${\mathcal {L}}^1$-measurable, as required. $\square$

Combining Lemma 7.12 with measure-theoretic argument allows us to extend Lemma 7.13 to hold for general Borel subsets. This provides a key step in the construction of an associated field ${{\varvec{F}}}$ in Sect. 8.1.

#### Lemma 7.14

For each $1 \le j \le n$, there is a null set ${\mathcal {K}}_j \subset {\mathbb {R}}$ containing ${\mathcal {N}}_{j,\mu }\cup {\mathcal {M}}_{j,\sigma }$ such that, for $t \notin {\mathcal {K}}_j$,

7.19 $$\begin{aligned} {\mathcal {F}}_{H_{j,+}^t}(S \cap \partial H_{j,+}^t) = -{\mathcal {F}}_{H_{j,-}^{t}}(S \cap \partial H_{j,-}^{t}) \quad \text {for any Borel set }S \subset \Omega . \end{aligned}$$

Moreover, for any such *S*, the mapping:

7.20 $$\begin{aligned} t \mapsto {\left\{ \begin{array}{ll} {\mathcal {F}}_{H_{j,+}^t}(S \cap \partial H_{j,+}^t) & \text {if } t \notin {\mathcal {K}}_j, \\ 0 & \text {if } t \in {\mathcal {K}}_j, \end{array}\right. } \end{aligned}$$

is ${\mathcal {L}}^1$-integrable satisfying

7.21 $$\begin{aligned} \int _{{\mathbb {R}}} |{\mathcal {F}}_{H_{j,+}^t}(S \cap \partial H_{j,+}^t)|\,\textrm{d}t \le \mu (S). \end{aligned}$$

#### Proof

  Consider the collection $\{ Q(a,b) :\, a,b \in {\mathbb {Q}}^n\}$ of cubes with rational endpoints in ${\mathbb {R}}^n$. Then, for ${\mathcal {L}}^n$–*a.e.* $x \in {\mathbb {R}}^n$, the conclusion of Lemma 7.12 is satisfied for all $x+Q(a,b)$ with *a*, *b* rational, and $x_i+a_i, x_i+b_i \notin {\mathcal {N}}_{i,\mu } \cup {\mathcal {M}}_{i,\sigma }$ for all $1 \le i \le n$. We then let ${\mathcal {Q}}_{\Omega }$ be the collection of such cubes $x+Q \Subset \Omega$ that are compactly contained in $\Omega$. Observe that ${\mathcal {Q}}_{\Omega }$ is a countable collection of open cubes generating the Borel $\sigma$-algebra of $\Omega$, which is also closed under finite intersections.

Writing ${\mathcal {Q}}_{\Omega } = \{Q_k\}_{k \in {\mathbb {N}}}$ and applying Lemma 7.13 to each $Q_k$, we obtain an associated null set ${\mathcal {N}}_{j,Q_k}$ for each $1 \le j \le n$, which contains ${\mathcal {N}}_{j,\mu } \cup {\mathcal {M}}_{j,\sigma }$ by construction. We set ${\mathcal {K}}_j:= \bigcup _k {\mathcal {N}}_{j,Q_k}$, which is a null set for each *j*. Then, for all $t \notin {\mathcal {K}}_j$ and $k \in {\mathbb {N}}$, (7.19) holds with $S = Q_k$, and the mapping in (7.20) with $S = Q_k$ is ${\mathcal {L}}^1$-measurable.

Now, for any $t \notin {\mathcal {K}}_j$, Lemma 7.6 ensures that ${\mathcal {F}}_{H_{j,\pm }^t}(\,\cdot \cap \partial H_{j,\pm }^t)$ are measures on $\Omega$, so we can extend (7.19) that holds for any $S = Q_k$ to all Borel sets $S \subset \Omega$ by using Lemma 2.2. For the second part, let ${\mathcal {D}}_j$ denote the set of Borel subsets $S \subset \Omega$ for which the result holds; that is, (7.20) is ${\mathcal {L}}^1$-integrable satisfying (7.21).

We now show that ${\mathcal {D}}_j$ contains all Borel subsets of $\Omega$, by a similar argument to that in [2, Remark 1.9], thereby establishing the result. For this, observe first that, if $S \subset \Omega$ is a Borel set, then, by property (iii) and Lemma 7.4,

$$\begin{aligned} |{\mathcal {F}}_{H_{j,+}^t}(S \cap \partial H_{j,+}^t) |\le \mu _t(S \cap \partial H_{j,+}^t) \quad \text{ for } {\mathcal {L}}^1\textit{--a.e.}\,\,t, \end{aligned}$$

where $\mu _t$ is given by the disintegration
Hence, if the mapping (7.20) is measurable, we can integrate in *t* to deduce (7.21) and thus infer that $S \in {\mathcal {D}}_j$. In particular, ${\mathcal {Q}}_{\Omega } \subset {\mathcal {D}}_j$, since the associated mapping is measurable.

We now claim that ${\mathcal {D}}_j$ is closed under the set differences and countable increasing unions. Indeed, if $A, B \in {\mathcal {D}}_j$ with $A \subset B$, then, for $t \notin {\mathcal {K}}_j$, we can write

$$\begin{aligned} {\mathcal {F}}_{H^t_{j,+}}((B \setminus A) \cap \partial H_{j,+}^t) = {\mathcal {F}}_{H^t_{j,+}}(B \cap \partial H_{j,+}^t) - {\mathcal {F}}_{H^t_{j,+}}(A \cap \partial H_{j,+}^t), \end{aligned}$$

which is evidently measurable in *t*, so that $B \setminus A \in {\mathcal {D}}_j$. Also, if $\{S_j\} \subset {\mathcal {D}}_j$ is any countable collection of pairwise disjoint Borel subsets in $\Omega$, then, setting $S = \bigcup _j S_j$, we have

$$\begin{aligned} \sum _{j}{\mathcal {F}}_{H^t_{j,+}}(S_j \cap \partial H_{j,+}^t) = {\mathcal {F}}_{H^t_{j,+}}(S \cap \partial H_{j,+}^t) \quad \text{ for } \text{ any } t \notin {\mathcal {K}}_j, \end{aligned}$$

so this is also measurable in *t*. Now, if $\{A_j\}\subset {\mathcal {D}}_j$ is a countable collection of Borel subsets which is not necessarily disjoint, define $\{S_k\}$ by setting $S_1 = A_1$ and $S_k = A_k {\setminus } \big ( \bigcup _{j=1}^{k-1} S_j \big )$ for each $k \ge 2$. Then $\{S_k\}$ are pairwise disjoint such that $\bigcup _k S_k = \bigcup _k A_k =:A$ and, by induction, each $S_k \in {\mathcal {D}}_j$, so that $A \in {\mathcal {D}}_j$. Using this, we also obtain $\Omega \in {\mathcal {D}}_j$, so ${\mathcal {D}}_j$ is a $\sigma$-algebra containing ${\mathcal {Q}}_{\Omega }$ and hence contains all Borel subsets $S \subset \Omega$, thereby establishing the result. $\square$

#### Lemma 7.15

Suppose that $Q = Q(a,b) \Subset \Omega$ such that $a_i,b_i \notin {\mathcal {K}}_{i}$ for all $1 \le i \le n$ and (7.11) holds. Then

$$\begin{aligned} {\mathcal {F}}_{Q}(S) = \sum _{i=1}^n \Big ( {\mathcal {F}}_{H_{i,+}^{a_i}}(S \cap \partial H_{i,+}^{a_i}) - {\mathcal {F}}_{H_{i,+}^{b_i}}(S \cap \partial H_{i,+}^{b_i}) \Big ) = - {\mathcal {F}}_{{{\overline{Q}}}^{\textrm{c}}}(S) \end{aligned}$$

for any Borel set $S \subset \partial Q$. In particular, for any cube *Q*, this holds for $x+Q$ for ${\mathcal {L}}^n$–*a.e.* $\,x \in {\mathbb {R}}^n$.

#### Proof

  The representations of ${\mathcal {F}}_Q(S)$ and ${\mathcal {F}}_{{{\overline{Q}}}^{\textrm{c}}}(S)$ follow by combining Lemma 7.11 with (7.19) from Lemma 7.14 and recalling that ${\mathcal {N}}_{j,\mu } \cup {\mathcal {M}}_{j,\sigma } \subset {\mathcal {K}}_j$ for each $1 \le j \le n$. For the last part, we apply Lemma 7.12 to see that (7.11) is satisfied for ${\mathcal {L}}^n$–a.e. $x \in {\mathbb {R}}^n$. By increasing the null set if necessary, we also require that $x_j+a_j, x_j+b_j \notin {\mathcal {K}}_j$ for all $1 \le j \le n$, where $Q = Q(a,b)$. $\square$

We now conclude this section with the proof of Lemma 7.12.

#### Proof of Lemma 7.12

Extending $\mu$ by zero, we view $\mu$ as a measure on ${\mathbb {R}}^n$ in what follows: Let $v, w \in {\mathbb {R}}^n$ be unit vectors with non-zero components such that, for all $1 \le i < j \le n$, the vectors $(v_i,v_j)$ and $(w_i,w_j)$ are linearly independent. For instance, we can define ${{\tilde{v}}}, {{\tilde{w}}} \in {\mathbb {R}}^n$ by setting ${{\tilde{v}}}_j = j$ and ${{\tilde{w}}}_j = j^2$ for each $1 \le j \le n$, which can then be normalized by letting $v = \frac{{{\tilde{v}}}}{|{{\tilde{v}}} |}$ and $w = \frac{{{\tilde{w}}}}{|{{\tilde{w}}} |}$. For this choice, we can readily verify that $v_iw_j \ne v_jw_i$ for all $i \ne j$, which implies linear independence. Now we define

7.22 $$\begin{aligned} \mathrm C:= \bigcup _{\beta \in {\mathbb {R}}} \big (\beta w +\partial ^2Q\big ). \end{aligned}$$

Then we divide the rest of the proof into three steps.

**1**. We first prove that, for the set $\mathrm C$ as defined in (7.22) above,

7.23 $$\begin{aligned} \mu (\alpha v + \textrm{C}) = 0 \quad \text{ for } {\mathcal {L}}^1\textit{--a.e.}\,\,\alpha \in {\mathbb {R}}. \end{aligned}$$

We establish this in two substeps, starting with the following algebraic result.

**1**.**1**. We show that there is $M \in {\mathbb {N}}$ sufficiently large such that, for any $\alpha _1,\alpha _2,\cdots ,\alpha _M \in {\mathbb {R}}$ distinct,

7.24 $$\begin{aligned} \bigcap _{k=1}^{M} ( \alpha _k v + \textrm{C}) = \varnothing . \end{aligned}$$

In fact, we can take $M = 2n(n-1) + 1$.

To see this, observe that, for each $x \in \alpha v + \textrm{C}$, there is some $\beta \in {\mathbb {R}}$ such that $y := x - \alpha v - \beta w \in \partial ^2Q$. Then there exist *i* and *j* with $1 \le i < j \le n$ such that $y_i \in \{ a_i, b_i\}$ and $y_j \in \{ a_j, b_j\}$. We can associate *y* as lying in the *corner* associated to $((i,j),(y_i,y_j))$, for which there are $$2^2 \left( {\begin{array}{c}n\\ 2\end{array}}\right) = 2n(n-1) = M-1$$ distinct choices.

Now, for this choice of *M*, suppose that there are distinct $\alpha _1,\cdots ,\alpha _M \in {\mathbb {R}}$ for which the claim fails to hold, so that there exists some $x \in \bigcap _{k=1}^M (\alpha _kv + \textrm{C})$. For each *k*, we can find $\beta _k$ such that

$$\begin{aligned} y_k := x - \alpha _{k} v - \beta _k w \in \partial ^2Q. \end{aligned}$$

By the pigeonhole principle, there exists two points that lie in the same corner, that is, there exist $k_1, k_2 \in \{1,2,\cdots ,M\}$ with $k_1 \ne k_2$ and $1 \le i < j \le n$ such that $(y_1)_i = (y_2)_i \in \{a_i,b_i\}$ and $(y_1)_j = (y_2)_j \in \{a_j,b_j\}$. We denote these common values by $c_i$ and $c_j$, respectively:

$$\begin{aligned} (y_q)_i&= x_i - \alpha _{k_q}v_i - \beta _{k_q} w_i= c_i, \\ (y_q)_j&= x_j - \alpha _{k_q}v_j - \beta _{k_q} w_j = c_j, \end{aligned}$$

for $q=1,2$. Rearranging for $x_i-c_i$ and $x_j-c_j$, we obtain the following equations:

$$\begin{aligned} \alpha _{k_1}v_i + \beta _{k_1} w_i&= \alpha _{k_2}v_i + \beta _{k_2} w_i, \\ \alpha _{k_1}v_j + \beta _{k_1} w_j&= \alpha _{k_2}v_j + \beta _{k_2} w_j. \end{aligned}$$

Since *v* and *w* have been chosen so that $(v_i,v_j)$ and $(w_i,w_j)$ are linearly independent, it follows that $\alpha _{k_1} = \alpha _{k_2}$ and $\beta _{k_1}=\beta _{k_2}$. However, this contradicts the fact that $\alpha _k$ are distinct, which implies that (7.24) holds as claimed.

**1**.**2**. For each $1 \le k \le M$, we will show there exists a countable set ${\mathcal {N}}_k$ with ${\mathcal {N}}_M \subset {\mathcal {N}}_{M-1} \subset \cdots \subset {\mathcal {N}}_1$ such that

$$\begin{aligned} \mu \big (\bigcap _{i=1}^k (\alpha _i v + \textrm{C})\big ) = 0 \end{aligned}$$

for all $\alpha _1, \cdots , \alpha _k \in {\mathbb {R}} \setminus {\mathcal {N}}_k$ distinct. Note that claim (7.23) is precisely the case $k=1$.

We show this by induction descending in *k*. First, we notice that this holds for $k = M$ with ${\mathcal {N}}_M = \varnothing$, since the previous step ensures that the intersection is empty.

For general $k < M$, suppose that there is a $\mu$-null set ${\mathcal {N}}_{k+1}$ as claimed, and consider any finite collection *F* of subsets $\Lambda = \{\alpha _1,\cdots ,\alpha _k\}$ containing *k* distinct elements taking values in ${\mathbb {R}} \setminus {\mathcal {N}}_{k+1}$. Observe that, if $\Lambda _1,\Lambda _2 \in F$ are distinct, then the union $\Lambda _1 \cup \Lambda _2$ contains at least $k+1$ elements and, by assumption on ${\mathcal {N}}_{k+1}$,

$$\begin{aligned} \mu \big (\bigcap _{\alpha \in \Lambda _1 \cup \Lambda _2} (\alpha v + \textrm{C})\big ) = 0. \end{aligned}$$

Now the inclusion–exclusion principle gives

7.25 $$\begin{aligned}&\mu \big (\bigcup _{\Lambda \in F} \bigcap _{\alpha \in \Lambda } (\alpha v + \textrm{C})\big ) \nonumber \\&\quad = \sum _{\Lambda \in F} \mu \big (\bigcap _{\alpha \in \Lambda } (\alpha v + \textrm{C})\big ) + \sum _{\begin{array}{c} S \subset F \\ |S |> 1 \end{array}} (-1)^{1+|F |}\sum _{\Lambda \in F} \mu \big (\bigcap _{\alpha \in \bigcup S} (\alpha v + \textrm{C})\big ). \end{aligned}$$

We see that all terms in the second sum are zero, since $\bigcup S$ always contains at least $k+1$ distinct elements of ${\mathbb {R}} \setminus {\mathcal {N}}_{k+1}$. Thus, we deduce from (7.25) that

$$\begin{aligned} \sum _{\Lambda \in F} \mu \big (\bigcap _{\alpha \in \Lambda }(\alpha v + \textrm{C}) \big ) =\mu \big ( \bigcup _{\Lambda \in F} \bigcap _{\alpha \in \Lambda }(\alpha _i^m v + \textrm{C}) \big ) \le \mu (\Omega ) < \infty . \end{aligned}$$

Since *F* is arbitrary, it follows that there are at most countably many collections $\Lambda _m = \{\alpha _1^m,\cdots ,\alpha _k^m\}$ of subsets of *k* distinct elements in ${\mathbb {R}} \setminus {\mathcal {N}}_{k+1}$ such that

$$\begin{aligned}\mu \big (\bigcup _{\alpha \in \Lambda _m} (\alpha v + \textrm{C})\big ) \ne 0. \end{aligned}$$

Thus, we take ${\mathcal {N}}_k = {\mathcal {N}}_{k+1} \cup \bigcup _m \Lambda _m$ which is ${\mathcal {L}}^1$-null, and observe that any collection $\alpha _1, \cdots , \alpha _k \in {\mathbb {R}}\setminus {\mathcal {N}}_k$ necessarily does not coincide with any of $\Lambda _m$, which implies that

$$\begin{aligned} \mu \big (\sum _{i=1}^k (\alpha _iv+\textrm{C})\big )=0. \end{aligned}$$

Therefore, the claim follows by induction.

**2**: We now show that $(\alpha v + \beta w) +Q$ satisfies (7.11) for ${\mathcal {L}}^2$–*a.e.* $(\alpha ,\beta ) \in {\mathbb {R}}^2$,

Let $\alpha \in {\mathbb {R}} \setminus {\mathcal {N}}_1$. For $\beta \in {\mathbb {R}}$ to be determined, let $x = \alpha v + \beta w$. Then $x+Q$ has faces intersecting with $\partial H^{\alpha v_i + \beta w_i + a_i}_{i,+}$ and $\partial H^{\alpha v_i + \beta w_i + b_i}_{i,-}$ for each $1 \le i \le n$. Moreover, by Lemma 7.4, we can estimate

$$\begin{aligned} \int _{{\mathbb {R}}} \mu ^{n-1}_{H^{\alpha v_i + t + a_i}_{i,+}} \big ( (\alpha v + \textrm{C}) \cap \partial H^{\alpha v_i + t + a_i}_{i,+}\big ) \,\textrm{d}t \le \mu (\alpha v + \textrm{C}) = 0, \end{aligned}$$

and similarly for $\partial H^{\alpha v_i + t + b_i}_{i,-}$. Thus considering $t = \beta w_i$ in the respective cases, by noting that $w_i \ne 0$,

$$\begin{aligned} \mu _{H^{\alpha v_i +\beta w_i + a_i}_{i,+}}^{n-1}(\alpha v+\textrm{C}) =\mu _{H^{\alpha v_i + \beta w_i + b_i}_{i,-}}^{n-1}(\alpha v+\textrm{C}) = 0 \quad \text{ for } {\mathcal {L}}^1\textit{--a.e.}\,\,\beta \in {\mathbb {R}}. \end{aligned}$$

For such $\beta$, we see that $x+Q$ with $x= \alpha v + \beta w$ satisfies (7.11), provided that $x+Q \Subset \Omega$. That is, for ${\mathcal {L}}^2$–*a.e.* $(\alpha ,\beta )\in {\mathbb {R}}^2$, the result holds for $x = \alpha v + \beta w$.

**3**: We now show that $x+Q$ satisfies (7.11) for ${\mathcal {L}}^n$–*a.e.* $x \in {\mathbb {R}}^n$.

For each $y \in P:= (\operatorname {span}\{v,w\})^{\perp }$, applying the above to $y+Q$, we obtain an ${\mathcal {L}}^2$-null set $\mathcal M_y \subset {\mathbb {R}}^2$ such that the result holds for $(\alpha v + \beta w) + y + Q$, whenever $(\alpha ,\beta ) \notin {\mathcal {M}}_y$. Then, for $\widetilde{{\mathcal {M}}}_y :=\{ y+ \alpha v + \beta w \,:\, (\alpha ,\beta ) \in {\mathcal {M}}_y\}$, $\mathcal H^2(\widetilde{{\mathcal {M}}}_y)=0$ by the area formula (cf. ([2, Theorem 2.71]). Then, setting ${\mathcal {M}} = \bigcup _{y \in P} {{\widetilde{M}}}_y$, by the Fubini Theorem, we have

$$\begin{aligned} {\mathcal {L}}^n({\mathcal {M}}) = \int _{P} {\mathcal {H}}^2(\widetilde{{\mathcal {M}}}_y) \,\textrm{d}{\mathcal {H}}^{n-2}(y) = 0. \end{aligned}$$

Therefore, (7.11) holds for all $x \notin {\mathcal {M}}$, provided that $x+Q \Subset \Omega$. $\square$

## Cauchy Flux III: Construction and Uniqueness of the Representing Divergence-Measure Field

We now employ the properties of the flux established in Sect. 7 to work towards the proof of Theorem 6.6. In particular, we define a candidate vector field ${{\varvec{F}}}$ to represent the flux, recover the balance law, and show that any such field must take this form.

### Construction of a Divergence-Measure Field

#### Theorem 8.1

Given a Cauchy flux, there exists a measure-valued vector field

$$\begin{aligned} {{\varvec{F}}}= (F_1,F_2,\cdots ,F_n) \in {\mathcal {M}}(\Omega ,{\mathbb {R}}^n) \end{aligned}$$

such that $|F_j |\le \mu$ for each $1 \le j \le n$ and

8.1 $$\begin{aligned} F_j(S) = \int _{{\mathbb {R}}} {\mathcal {F}}_{H_{j,+}^t}(S \cap \partial H_{j,+}^t) \,\textrm{d}t \quad \text{ for } \text{ any } \text{ Borel } \text{ set } S \subset \Omega . \end{aligned}$$

#### Proof

  By Lemma 7.14, we know that integral (8.1) is well-defined for any Borel set $S \subset \Omega$ (understanding that it is zero on the null set ${\mathcal {K}}_j$) and satisfies the estimate:

8.2 $$\begin{aligned} |F_j(S) |\le \mu (S). \end{aligned}$$

From this, it also follows that $F_j(\varnothing ) = 0$. By the additivity property (i) of the flux and the linearity of the integral, we know that $F_j$ is additive on Borel sets. To show the countable additivity, let $\{S_k\}$ be a collection of pairwise disjoint Borel subsets of $\Omega$, and let $S = \bigcup _k S_k$. Then, by finite additivity and (8.2), we have

$$\begin{aligned} \Big |F_j(S) - \sum _{k=1}^M F_j(S_k) \Big \vert = \Big |F_j\big (S \setminus \bigcup _{k=1}^M S_k \big )\Big \vert \le \mu \big (S \setminus \bigcup _{k=1}^M S_k \big ) \rightarrow 0 \quad \text{ as } M \rightarrow \infty . \end{aligned}$$

This shows that each $F_j$ is a signed measure on $\Omega$, as required. $\square$

#### Theorem 8.2

(Recovery of the balance law) The field ${{\varvec{F}}}$ constructed in Theorem 8.1 lies in $\mathcal{D}\mathcal{M}^{{{\,\textrm{ext}\,}}}(\Omega )$ and satisfies

$$\begin{aligned} -\operatorname {div}{{\varvec{F}}}= \sigma \quad \text {in } \Omega . \end{aligned}$$

In particular, for any $U \Subset \Omega$,

8.3 $$\begin{aligned} {\mathcal {F}}_U(\partial U) = \sigma (U) = \langle {{\varvec{F}}}\cdot \nu , \,\mathbbm {1}_{\Omega } \rangle _{\partial U}. \end{aligned}$$

#### Proof

To simplify notation, we write $H_{j,+}^t = H_j^t$. Let $\rho _{\delta }$ be a standard mollifier, and define ${{\varvec{F}}}_{\delta }:= {{\varvec{F}}}*\rho _{\delta }$. Then, for any cube $Q = Q(a,b) \Subset \Omega$ and $0< \delta < \textrm{dist}(Q,\partial \Omega )$, since ${{\varvec{F}}}_{\delta }$ is smooth on ${{\overline{Q}}}$, the classical Divergence Theorem gives

$$\begin{aligned} \begin{aligned} \int _{Q} \operatorname {div}{{\varvec{F}}}_{\delta } (x) \,\textrm{d}x&= -\int _{\partial Q} {{\varvec{F}}}_{\delta }(x) \cdot \nu _{\partial Q} (x)\,\textrm{d}{\mathcal {H}}^{n-1} (x)\\&= - \int _{\partial Q} \int _{{\mathbb {R}}^n} \rho _{\delta } (x-y)\,\nu _{\partial Q} (x) \cdot \textrm{d}{{\varvec{F}}}(y)\,\textrm{d}{\mathcal {H}}^{n-1} (x), \end{aligned} \end{aligned}$$

where $\nu _{\partial Q}$ is the inwards normal to $\partial Q$.

We consider the face: $\partial Q \cap \partial H_1^{a_1}$ on which $\nu _{\partial Q}= e_1$. Then, by construction of $F_j$ from Theorem 8.1, we know that the disintegration

$$\begin{aligned} F_j = {\mathcal {L}}^1 \otimes _{\partial H_{j}^t} F_{j,t} = {\mathcal {L}}^1 \otimes _{\partial H_{j}^t} {\mathcal {F}}_{H_{j}^t}(\,\cdot \cap \partial H_{j}^t) \end{aligned}$$

holds. Using this and writing $Q = (a_1,b_1) \times Q'$, we can write

$$\begin{aligned} \begin{aligned}&\int _{\partial Q \cap \partial H_1^{a_1}} \int _{{\mathbb {R}}^n} \rho _{\delta } (x-y)\,\nu _{\partial Q}(x)\cdot \textrm{d}{{\varvec{F}}}(y)\,\textrm{d}{\mathcal {H}}^{n-1}(x)\\&\quad = \int _{{\mathbb {R}}^{n-1}} \int _{{\mathbb {R}}} \int _{{\mathbb {R}}^{n-1}} \chi _{Q'}(x')\rho _{\delta }(a_1-y_1,x'-y')\, \textrm{d}F_{1,y_1}(y) \,\textrm{d}y_1\,\textrm{d}x' \\&\quad = \int _{{\mathbb {R}}^{n-1}} \int _{{\mathbb {R}}} \int _{{\mathbb {R}}^{n-1}} \chi _{Q'-z'}(y')\rho _{\delta }(z_1,z')\, \textrm{d}F_{1,a_1-z_1}(a_1-z_1,y') \,\textrm{d}z_1\,\textrm{d}z' \\&\quad = \int _{{\mathbb {R}}^n} \rho _{\delta }(z) F_{1,a_1-z_1}(\{a_1 - z_1\} \times (Q'-z'))\,\textrm{d}z \\&\quad = \int _{{\mathbb {R}}^n} \rho _{\delta }(z) {\mathcal {F}}_{-z + H_1^{a_1}}(-z+(\partial Q \cap \partial H_1^{a_1})) \,\textrm{d}z, \end{aligned} \end{aligned}$$

where we have made the change of variables $y_1 \mapsto z_1 = a_1-y_1$ and $x' \mapsto z' = x'-y'$. The remaining sides can be computed similarly. Writing $Q_z = z+Q$ that has faces $H_{i}^{a_i-z_i}$ and $H_i^{b_i-z_i}$, we deduce

$$\begin{aligned} \begin{aligned} \int _{Q} \operatorname {div}{{\varvec{F}}}_{\delta } (x) \,\textrm{d}x&= \sum _{i=1}^n \int _{{\mathbb {R}}^n} \rho _{\delta }(z) {\mathcal {F}}_{H_j^{a_j-z_j}}(\partial Q_{-z} \cap \partial H_j^{a_j-z_j}) \,\textrm{d}z \\&\quad - \sum _{i=1}^n \int _{{\mathbb {R}}^n} \rho _{\delta }(z) {\mathcal {F}}_{H_j^{b_j-z_j}}(\partial Q_{-z} \cap \partial H_j^{b_j-z_j})\,\textrm{d}z. \end{aligned} \end{aligned}$$

Now, by Lemmas 7.12 and 7.15, for ${\mathcal {L}}^n$–*a.e.*  *z* with $|z|\le \textrm{dist}(Q,\partial \Omega )$, we have

$$\begin{aligned} \begin{aligned}&\sum _{i=1}^n \int _{{\mathbb {R}}^n} \Big ({\mathcal {F}}_{H_j^{a_j-z_j}}(\partial Q_{-z} \cap \partial H_j^{a_j-z_j}) - {\mathcal {F}}_{H_j^{b_j-z_j}}(\partial Q_{-z} \cap \partial H_j^{b_j-z_j}) \Big )\\&\quad = {\mathcal {F}}_{Q_{-z}}(\partial Q_{-z}) = \sigma (Q_{-z}). \end{aligned} \end{aligned}$$

Thus, we obtain

$$\begin{aligned} \int _Q \operatorname {div}{{\varvec{F}}}_{\delta }(z) \,\textrm{d}x = -\int _{{\mathbb {R}}^n} \rho _{\delta }(z) \sigma (Q+z) \,\textrm{d}z = -\int _Q \sigma *{{\tilde{\rho }}}_{\delta }(x) \,\textrm{d}x, \end{aligned}$$

where ${{\tilde{\rho }}}_{\delta }(x) = \rho _{\delta }(-x)$ is also a mollifier. Since this holds for all cubes $Q \Subset \Omega$ whenever $0<\delta < \textrm{dist}(Q,\partial \Omega )$, it follows that

$$\begin{aligned} -\operatorname {div}{{\varvec{F}}}*\rho _{\delta } = \sigma *{{\tilde{\rho }}}_{\delta } \quad \text {in } \Omega ^{\delta }. \end{aligned}$$

Sending $\delta \rightarrow 0$, we deduce that ${{\varvec{F}}}\in \mathcal{D}\mathcal{M}^{{{\,\textrm{ext}\,}}}(\Omega )$ with $-\operatorname {div}{{\varvec{F}}}= \sigma$. Finally, it follows from the balance law (6.17) that, for any $U \Subset \Omega$,

$$\begin{aligned} {\mathcal {F}}_U(\partial U) = \sigma (U) = -\int _U \textrm{d}(\operatorname {div}{{\varvec{F}}}), \end{aligned}$$

and, by (2.11), this is equal to $\langle {{\varvec{F}}}\cdot \nu , \,\mathbbm {1}_{\Omega }\rangle _{\partial U}$, thereby establishing (8.3). $\square$

### Uniqueness of the Field

We now show that the constructed field ${{\varvec{F}}}$ is unique. Note that, in Theorem 8.1, the divergence-measure field ${{\varvec{F}}}$ has been constructed componentwise by integrating the fluxes on $\partial H_{j,+}^t$ with respect to the Lebesgue measure. However, this choice of the measure is seemingly arbitrary, and one may wonder if there is a *singular part* that is missed with this construction. This possibility is essentially ruled out by Theorem 4.1; however, this cannot be directly applied with $U = H_{j,+}^t$ since . Nevertheless, using the localization results from Sect. 5 and Sect. 7.1, we can adapt the result as follows:

#### Lemma 8.3

Let ${{\varvec{F}}}= (F_1,F_2,\cdots ,F_n) \in \mathcal{D}\mathcal{M}^{{{\,\textrm{ext}\,}}}(\Omega )$. Consider the disintegration:

$$\begin{aligned} F_j = \tau \otimes _{\partial H_{j,+}^t} {{\tilde{F}}}_{j,t} \quad \text{ for } 1 \le j \le n, \end{aligned}$$

where $H_{j,+}^t = \{ x \in {\mathbb {R}}^n: x_j > t\}$, extending $F_j$ by zero to a measure on ${\mathbb {R}}^n$. Then $\tau \ll {\mathcal {L}}^1$ and, for ${\mathcal {L}}^1$–*a.e.* $t \in {\mathbb {R}}$, $F_{j,t}:= D_{{\mathcal {L}}^1}\tau (t) {{\tilde{F}}}_{j,t}$ is well-defined and satisfies
as measures. In particular, $F_j = {\mathcal {L}}^1 \otimes _{\partial H_{j,+}^t} F_{j,t}.$

#### Proof

  By Theorem 6.7, the divergence-measure field ${{\varvec{F}}}$ defines a Cauchy flux $\widetilde{{\mathcal {F}}}$ via its normal trace with $\mu = |{{\varvec{F}}}|$. Fix $t \in {\mathbb {R}}$ be such that
to some limiting measure $\mu _t^{n-1}$, which is satisfied for ${\mathcal {L}}^1$–*a.e.* $t\,$ by Lemma 7.4 applied with $\mu = |{{\varvec{F}}}|$. Then, by Lemma 7.6, there is a well-defined measure $\widetilde{{\mathcal {F}}}_{H_{j,+}^t}(\cdot )$ on $\Omega$ such that

8.4 $$\begin{aligned} \widetilde{{\mathcal {F}}}_{H_{j,+}^t}(S) =({{\varvec{F}}}\cdot \nu )_{\partial ({{\widetilde{\Omega }}}^{\delta } \cap H_{j,+}^t)}(S) \end{aligned}$$

for ${\mathcal {L}}^1$–*a.e.* $\delta \in (0,1)$ with ${{\widetilde{\Omega }}}^{\delta } \in {\mathcal {O}}_{|{{\varvec{F}}}|}$ and for any Borel set $S \subset {{\widetilde{\Omega }}}^{\delta } \cap \partial H_{j,+}^t$, where ${{\widetilde{\Omega }}}^{\delta }$ is defined in (7.6). For any such $\delta >0$, we can apply Theorem 4.1 to $U:= {{\widetilde{\Omega }}}^{\delta } \cap H_{j,+}^t \Subset \Omega$, which gives

8.5 $$\begin{aligned} \int _0^{\delta } \langle {{\varvec{F}}}\cdot \nu , \,\phi \rangle _{\partial ({{\widetilde{\Omega }}}^{\delta } \cap H_{j,+}^t)^{\varepsilon }} \,\textrm{d}\varepsilon = \int _{U \setminus \overline{U^{\delta }}} \,\phi \,\textrm{d}\overline{\nabla d_U \cdot {{\varvec{F}}}} \quad \text {for any } \phi \in \textrm{Lip}_{\textrm{c}}(\Omega ). \end{aligned}$$

Now suppose further that $\phi$ is compactly supported in ${{\widetilde{\Omega }}}^{2\delta }$, and set

$$\begin{aligned} V_{t,\delta } := {{\widetilde{\Omega }}}^{2\delta } \cap (H_{j,+}^t \setminus \overline{H_{j,+}^{t+\delta }}). \end{aligned}$$

Then, for each $\varepsilon \in (0,\delta )$, observe that $d_U(x) = d_{H_{j,+}^t}(x) = \varepsilon$ for $x \in {{\widetilde{\Omega }}}^{2\delta } \cap \partial H_{j,+}^{t+\varepsilon }$, since $\textrm{dist}(x,\partial {{\widetilde{\Omega }}}^{\delta }) \ge \min \{\delta ,\delta ^{-1}\}$ and $\delta <1$. Hence, $V_{t,\delta } \subset U \setminus {\overline{U}}^{\delta }$ and $d_U = d_{H_{j,+}^t}$ on $V_{t,\delta }$ that is open. Then, by the product rule (Theorem 2.7), we infer
8.6as measures. In addition, for each $\varepsilon \in (0,\delta )$, the above implies that

$$\begin{aligned} ({{\widetilde{\Omega }}}^{\delta } \cap H_{j,+}^t)^{\varepsilon } \cap V_{\delta } = {{\widetilde{\Omega }}}^{\delta } \cap H_{j,+}^{t+\varepsilon } \cap V_{\delta }. \end{aligned}$$

By localization (Theorem 5.3), we have
8.7by using (8.4). Combining (8.7) with (8.6) in (8.5), we arrive at

$$\begin{aligned} \int _0^{\delta } \int _{\Omega \cap \partial H_{j,+}^{t+\varepsilon }} \phi \,\textrm{d}\widetilde{{\mathcal {F}}}_{H_{j,+}^{t+\varepsilon }} \,\textrm{d}\varepsilon = \int _{H_{j,+}^t \setminus \overline{H_{j,+}^{t+\delta }}} \,\phi \,\textrm{d}F_j \quad \text {for any } \phi \in \textrm{Lip}_{\textrm{c}}(\Omega ^{2\delta }). \end{aligned}$$

This shows that
as measures. Since $\delta \in (0,1)$ is arbitrary and ${\mathcal {L}}^1$–*a.e.* $t \in {\mathbb {R}}$ can be taken, it follows that

$$\begin{aligned} F_j = {\mathcal {L}}^1 \otimes _{\partial H_{j,+}^t} \widetilde{{\mathcal {F}}}_{H_{j,+}^{t}} \end{aligned}$$

as measures in ${\mathbb {R}}^n$. Then the result follows by using (8.4). $\square$

#### Theorem 8.4

(Uniqueness of the representing field) Given a Cauchy flux ${\mathcal {F}}$, suppose that there exists a field ${{\varvec{G}}}\in \mathcal{D}\mathcal{M}^{{{\,\textrm{ext}\,}}}(\Omega )$ such that

$$\begin{aligned} {\mathcal {F}}_{U}(S) = ({{\varvec{G}}}\cdot \nu )_{\partial U}(S) \quad \text{ for } \text{ any } U \in {\mathcal {O}}_{\mu } \text{ and } \text{ any } \text{ Borel } \text{ set } S \subset \partial U. \end{aligned}$$

Then ${{\varvec{F}}}= {{\varvec{G}}}$ as measures, where ${{\varvec{F}}}$ is the field constructed in Theorem 8.1.

#### Proof

  By Theorem 6.7, the field ${{\varvec{G}}}= (G_1,G_2,\cdots ,G_n)$ defines a Cauchy flux via the normal trace, which is denoted by ${\mathcal {G}}$. Let $1 \le j \le n$, and consider $t \in {\mathbb {R}}$ for which both limits:
exist as $\varepsilon \rightarrow 0$, which is satisfied by ${\mathcal {L}}^1$–*a.e.* $t \in {\mathbb {R}}$. For any such *t*, applying Lemma 7.6 to ${\mathcal {F}}$ and ${\mathcal {G}}$ with $U = H_{j,+}^t$, we see that

$$\begin{aligned} {\mathcal {F}}_{H_{j,+}^t}(\,\cdot \cap \Omega \cap \partial H_{j,+}^t) = {\mathcal {G}}_{H_{j,+}^t}(\,\cdot \cap \Omega \cap \partial H_{j,+}^t) \end{aligned}$$

are well-defined and agree as measures, since they agree on ${{\widetilde{\Omega }}}^{\delta } \cap \partial H_{j,+}^t$ for ${\mathcal {L}}^1$–*a.e.* $\delta >0$. Then, applying Lemma 8.3 above, we conclude
This is precisely how ${{\varvec{F}}}$ has been defined in Theorem 8.1. Therefore, ${{\varvec{F}}}= {{\varvec{G}}}$. $\square$

## Cauchy Flux IV: Local Recovery and Applications

In Sect. 8, we have constructed an associated field ${{\varvec{F}}}\in \mathcal{D}\mathcal{M}^{{{\,\textrm{ext}\,}}}(\Omega )$ and have shown that any field representing the flux ${\mathcal {F}}$ must take this form. It remains to show that the field ${{\varvec{F}}}$ represents the flux in the sense of Theorem 6.6, which is the content of the following subsection.

### Local Recovery of the Flux

The precise statement of the local recovery result is following:

#### Theorem 9.1

(Local recovery of the flux)  Let ${\mathcal {F}}$ be a Cauchy flux, and let ${{\varvec{F}}}\in \mathcal{D}\mathcal{M}^{{{\,\textrm{ext}\,}}}(\Omega )$ be the associated field from Theorem 8.1. Then

9.1 $$\begin{aligned} {\mathcal {F}}_{U}(S) = ({{\varvec{F}}}\cdot \nu )_{\partial U}(S) \end{aligned}$$

for any $U \in {\mathcal {O}}_{\mu }$ and any Borel set $S \subset \partial U$.

We first show this local recovery result holds on *almost all* cubes, before extending to general open sets in ${\mathcal {O}}_{\mu }$.

#### Lemma 9.2

Let ${\mathcal {F}}$ be a Cauchy flux, and let ${{\varvec{F}}}$ be the associated field from Theorem 8.1. Then, for any cube *Q*, there exists a null set ${\mathcal {N}} \subset {\mathbb {R}}^n$ such that, for any $x \notin {\mathcal {N}}$, if $Q_x = x+Q\Subset \Omega$, then

9.2 $$\begin{aligned} {\mathcal {F}}_{Q_x}(S) = ({{\varvec{F}}}\cdot \nu )_{\partial Q_x}(S) = - {\mathcal {F}}_{\,{\overline{Q}}_x^{\textrm{c}}}(S) = - ({{\varvec{F}}}\cdot \nu )_{\partial {\overline{Q}}_x^{\textrm{c}}}(S) \end{aligned}$$

for any Borel set $S \subset \partial Q_x$, where the flux on ${\overline{Q}}^{\textrm{c}}_x$ in (9.2) is defined in Remark 7.7.

#### Proof

  By Theorem 6.7, ${{\varvec{F}}}$ defines a Cauchy flux $\widetilde{{\mathcal {F}}}$ via its normal trace. Also, by construction of ${{\varvec{F}}}=(F_1,F_2,\cdots ,F_n)$ from Theorem 8.1 and Lemma 8.3 applied to ${{\varvec{F}}}$, we know that there is a null set ${\mathcal {N}}_j \subset {\mathbb {R}}$ such that

9.3 $$\begin{aligned} \widetilde{{\mathcal {F}}}_{ H_{j,+}^t} = F_j = {\mathcal {F}}_{ H_{j,+}^t} \end{aligned}$$

as measures whenever $t \notin {\mathcal {N}}_j$. Now, for any cube $Q = Q(a,b)$, consider $Q_x = x+Q$ for $x \in {\mathbb {R}}^n$ such that $Q_x \Subset \Omega$, Lemma 7.15 holds for both ${\mathcal {F}}$ and $\widetilde{{\mathcal {F}}}$, and Lemma 7.14 applies at the endpoints $x_j+a_j$ and $x_j+b_j$ for each $1\le j \le n$ (so, in particular, they do not lie in ${\mathcal {N}}_j$). By Lemma 7.12, this holds for ${\mathcal {L}}^n$–*a.e.*  *x* such that $Q_x \Subset \Omega$. Then, for such *x*, using (9.3), we infer that, for any Borel set $S \subset \partial Q_x$,

$$\begin{aligned} \begin{aligned} {\mathcal {F}}_{Q_x}(S)&= \sum _{i=1}^n \Big ( {\mathcal {F}}_{H_{i,+}^{x_i+a_i}}(S \cap \partial H_{i,+}^{x_i+a_i}) - {\mathcal {F}}_{H_{i,+}^{x_i+b_i}}(S \cap \partial H_{i,+}^{x_i+ b_i}) \Big ) \\&= \sum _{i=1}^n \Big ( \widetilde{{\mathcal {F}}}_{H_{i,+}^{x_i+a_i}}(S \cap \partial H_{i,+}^{x_i+a_i}) - \widetilde{{\mathcal {F}}}_{H_{i,+}^{x_i+b_i}}(S \cap \partial H_{i,+}^{x_i+ b_i}) \Big ) \\&= \widetilde{{\mathcal {F}}}_{Q_x}(S) = ({{\varvec{F}}}\cdot \nu )_{\partial Q_x}(S). \end{aligned} \end{aligned}$$

Since Lemma 7.15 also gives ${\mathcal {F}}_{Q_x} = - {\mathcal {F}}_{\,{\overline{Q}}_x^{\textrm{c}}}$ and similarly for $\widetilde{{\mathcal {F}}}$, (9.2) holds as required. $\square$

We now extend Lemma 9.2 by approximating a general domain $U \in {\mathcal {O}}_{\mu }$ via finite unions of *good* cubes for which the local recovery holds, and argue that we can pass to the limit. To do this, we first show the existence of a family of the said good cubes.

#### Lemma 9.3

There exists a countable collection ${\mathcal {Q}}_{\Omega }$ of cubes such that

(i) For each $Q \in {\mathcal {Q}}_{\Omega }$, $Q, {\overline{Q}}^{\textrm{c}} \in \widetilde{{\mathcal {O}}}_{\mu }$ and $Q \Subset \Omega$.
(ii) If $Q_1, Q_2 \in {\mathcal {Q}}_{\Omega }$ intersect non-trivially, then $Q_1 \cap Q_2 \in {\mathcal {Q}}_{\Omega }$.
(iii) Every open set $U \Subset \Omega$ can be written as a union of cubes in ${\mathcal {Q}}_{\Omega }$.
(iv) For each $Q \in {\mathcal {Q}}_{\Omega }$, the recovery of the flux on *Q* and its complement can be achieved in the sense that
  $$\begin{aligned} {\mathcal {F}}_{Q} = ({{\varvec{F}}}\cdot \nu )_{\partial Q} = - \mathcal F_{\,{\overline{Q}}^{\textrm{c}}} = - ({{\varvec{F}}}\cdot \nu )_{\partial {\overline{Q}}^{\textrm{c}}} \end{aligned}$$
  holds as measures on $\partial Q$.
(v) For each $Q \in {\mathcal {Q}}_{\Omega }$, the localization and two-sided properties of Lemmas 7.11 and 7.15 hold for both the flux ${\mathcal {F}}$ and the normal trace ${{\varvec{F}}}\cdot \nu$.
(vi) $\mu (\partial Q) = 0$ and $\mu _{Q}^{n-1}(\partial ^2Q)=\mu _{{\overline{Q}}^{\,\textrm{c}}}^{n-1}(\partial ^2Q)=0$ for each $Q \in {\mathcal {Q}}_{\Omega }$.

#### Proof

  Consider the collection $\{ Q(a,b):\,a,b \in {\mathbb {Q}}^n\}$ of open cubes with rational endpoints in ${\mathbb {R}}^n$, which is countable and closed under finite intersections.

Then, for ${\mathcal {L}}^n$–*a.e.* $x \in {\mathbb {R}}^n$, Lemma 9.2 applies to each $Q_x := x + Q(a,b)$ such that $Q_x \Subset \Omega$:

9.4 $$\begin{aligned} {\mathcal {F}}_{Q_x}(S)&= \sum _{i=1}^n \Big ( {\mathcal {F}}_{H_{i,+}^{x_i+a_i}}(S \cap \partial H_{i,+}^{x+a_i}) - {\mathcal {F}}_{H_{i,+}^{x_i+b_i}}(S \cap \partial H_{i,+}^{x_i+b_i})\Big ) \nonumber \\&= \sum _{i=1}^n \Big (- {\mathcal {F}}_{H_{i,-}^{x_i+a_i}}(S \cap \partial H_{i,-}^{x+a_i}) + {\mathcal {F}}_{H_{i,-}^{x_i+b_i}}(S \cap \partial H_{i,-}^{x_i+b_i})\Big )\nonumber \\&= - {\mathcal {F}}_{\,\overline{Q_x}^{\textrm{c}}}(S) = ({{\varvec{F}}}\cdot \nu )_{\partial Q_x}(S) = - ({{\varvec{F}}}\cdot \nu )_{\partial \overline{Q_x}^{\textrm{c}}}(S) \end{aligned}$$

for each Borel set $S \subset \partial Q_x$. Moreover, we can ensure that (7.11) holds for each $Q_x$ by Lemma 7.12. We can also choose *x* such that (9.4) holds with the normal trace ${{\varvec{F}}}\cdot \nu$ in place of the flux ${\mathcal {F}}$, by using Theorem 6.7, so that each $Q_x$ satisfies properties (iv) and (v). In addition, we can ensure that the endpoints $x_j+a_j$ and $x_j+b_j$ do not lie in ${\mathcal {N}}_{j,\mu }$ from Definition 7.9 for each $1 \le j \le n$ so that $\mu (\partial Q_x) = 0$. Also, Lemma 7.10 holds for $Q_x$ as (7.11) is satisfied, which ensures that $\mu _{Q}^{n-1}(\partial ^2Q)=\mu _{\,{\overline{Q}}^{\textrm{c}}}^{n-1}(\partial ^2Q)=0$. Hence, $Q_x$ satisfies (vi). Now, for such *x*, we let

$$\begin{aligned} {\mathcal {Q}}_{\Omega } = \{ Q_x = x+Q(a,b)\,:\,Q_x \Subset \Omega , a, b \in {\mathbb {Q}}^n\}. \end{aligned}$$

By construction, (i) and (ii) hold. Since every open set *U* can be written as the union of cubes with rational endpoints, by considering $-x + U$, we see that (iii) also holds. Therefore, ${\mathcal {Q}}_{\Omega }$ satisfies (i)–(vi) as required. $\square$

Next, we show that local recovery holds for unions of cubes from this good collection.

#### Lemma 9.4

Given ${\mathcal {Q}}_{\Omega }$ as in Lemma 9.3, denote ${\mathcal {V}}_{\Omega }$ as the set of all finite unions *V* of cubes in ${\mathcal {Q}}_{\Omega }$ given by

9.5 $$\begin{aligned} V = \textrm{int}\big ( \bigcup _{i=1}^k \overline{Q_i} \big ) \quad \textrm{for }\, Q_1, \cdots , Q_k \in {\mathcal {Q}}_{\Omega }. \end{aligned}$$

Then, for any $V \in {\mathcal {V}}_{\Omega }$, $\,V, {{\overline{V}}}^{\textrm{c}} \in \widetilde{{\mathcal {O}}}_{\mu }$ and

9.6 $$\begin{aligned} {\mathcal {F}}_{V} = - {\mathcal {F}}_{\,{\overline{V}}^{\textrm{c}}} = ({{\varvec{F}}}\cdot \nu )_{\partial V} = - ({{\varvec{F}}}\cdot \nu )_{\partial {\overline{V}}^{\textrm{c}}} \end{aligned}$$

as measures on $\Omega$.

#### Proof

  Since $|{{\varvec{F}}}|\le \mu$, by Theorem 6.7, we can view the normal trace ${{\varvec{F}}}\cdot \nu$ as a flux $\widetilde{{\mathcal {F}}}$ with the same $\mu$. Now we divide the proof into four steps.

**1.** We first show that every $V \in {\mathcal {V}}_{\Omega }$ can be written as a union of disjoint cubes. It suffices to establish this claim in the case of two cubes, since the general case follows by inductively replacing the cubes that overlap. Suppose that $Q_1 = Q(a,b)$ and $Q_2 = Q(c,d)$ are contained in the union of *V* and intersect non-trivially. Denote ${\mathcal {S}}$ as the set of cubes $Q(x,y) \subset Q_1 \cup Q_2$ such that $x_j,y_j \in \{a_j,b_j,c_j,d_j\}$ and $x_j < y_j$ for all $1 \le j \le n$, which defines a finite collection of cubes in ${\mathcal {Q}}_{\Omega }$. We use ${\mathcal {S}}$ to define ${{\widetilde{S}}}$ by discarding all cubes $Q \in {\mathcal {S}}$ for which there is ${{\widetilde{Q}}} \in {\mathcal {S}}$ with ${{\widetilde{Q}}} \subset Q$ (this can be done inductively in any order). Then ${{\widetilde{S}}}$ is a disjoint collection of cubes such that

$$\begin{aligned} \text {int}\big ( \bigcup _{Q \in {{\widetilde{S}}}} {{\overline{Q}}} \big ) = \text {int}\big (\overline{Q_1} \cup \overline{Q_2}\big ), \end{aligned}$$

so that $Q_1$ and $Q_2$ can be replaced by this collection ${{\widetilde{S}}}$.

**2**. We now show that, for any $V \in {\mathcal {V}}_{\Omega }$,

9.7 $$\begin{aligned} \limsup _{\varepsilon \rightarrow 0} \frac{1}{2\varepsilon } \, \mu (V^{-\varepsilon } \setminus \overline{V^\varepsilon }) < \infty . \end{aligned}$$

That is, $\mu (\partial V) = 0$ and $V, {{\overline{V}}}^{\textrm{c}} \in \widetilde{{\mathcal {O}}}_{\mu }$.

We induct on the number of cubes in the union to show this. If $V = Q$, this follows by properties (i) and (vi) of ${\mathcal {Q}}_{\Omega }$. Assuming that this holds for some *V*, we consider a disjoint cube $Q \in {\mathcal {Q}}_{\Omega }$ and put ${{\widetilde{V}}} = \text {int}\big ({{\overline{V}}} \cup {{\overline{Q}}}\big )$. Then, arguing analogously as in the proof of Lemma 7.2, we have

$$\begin{aligned} {{\widetilde{V}}}^{-\varepsilon } \setminus \overline{{{\widetilde{V}}}^\varepsilon } \subset (V^{-\varepsilon } \setminus \overline{V^{\varepsilon }}) \cup (Q^{-\varepsilon } \setminus \overline{Q^{\varepsilon }}) \quad \text {for any } \varepsilon >0. \end{aligned}$$

Integrating this over $\frac{1}{2\varepsilon }\, \mu$ and sending $\varepsilon \rightarrow 0$ yield (9.7), by noting that both *Q* and *V* satisfy (9.7).

**3**. We next show that, for every $V \in {\mathcal {V}}_{\Omega }$ and any cube $Q \in {\mathcal {Q}}_{\Omega }$ contained in the union,
and the same holds with $\widetilde{{\mathcal {F}}}$ in place of ${\mathcal {F}}$.

Writing $V \in {\mathcal {V}}_{\Omega }$ as a union of disjoint cubes $\{Q_i\}_{i=1}^k$, by property (vi) of ${\mathcal {Q}}_{\Omega }$, we know that

$$\begin{aligned} \mu _{Q_i}^{n-1}(\partial ^2Q_i)=\mu _{{\overline{Q}}_i^{\textrm{c}}}^{n-1}(\partial ^2Q_i)=0 \quad \text { for each } 1 \le i \le k. \end{aligned}$$

Then, by Lemma 7.3(ii), we see that

$$\begin{aligned} \mu _V^{n-1}(\partial V \cap \partial ^2Q_i) =\mu _{\,{\overline{V}}^{\textrm{c}}}^{n-1}(\partial V \cap \partial ^2Q_i)=0\quad \text { for each } 1 \le i \le k. \end{aligned}$$

By Definition 6.4(iii) applied to both ${\mathcal {F}}$ and $\widetilde{{\mathcal {F}}}$, the result follows.

**4**. We prove that, if $V \in {\mathcal {V}}_{\Omega }$ is the disjoint union of cubes $\{Q_i\}_{i=1}^k$ in ${\mathcal {Q}}_{\Omega }$, then

9.8 $$\begin{aligned} {\mathcal {F}}_{V} = \sum _{i=1}^k {\mathcal {F}}_{Q_k}, \quad {\mathcal {F}}_{\,{{\overline{V}}}^{\textrm{c}}} = \sum _{i=1}^k {\mathcal {F}}_{\,{{\overline{Q}}}_k^{\textrm{c}}} \end{aligned}$$

as measures in $\Omega$, and the same holds with $\widetilde{{\mathcal {F}}}$ in place of ${\mathcal {F}}$.

We prove (9.8) by inducting on the number of cubes, where the case $k=1$ follows by Lemma 9.3(iv). While we only consider ${\mathcal {F}}$, the argument for $\widetilde{{\mathcal {F}}}$ is analogous since it is a Cauchy flux with the same $\mu$ and $\sigma$, and agrees with ${\mathcal {F}}$ on cubes $Q \in {\mathcal {Q}}_{\Omega }$.

Assuming that $V \in {\mathcal {V}}_{\Omega }$ satisfies (9.8) and $Q \in {\mathcal {Q}}_{\Omega }$ is disjoint from *V*, define ${{\widetilde{V}}}:= \text {int}({{\overline{V}}} \cup {{\overline{Q}}})$. By the localization property (ii) applied with $A=\Omega \setminus {{\overline{Q}}}$, we see that ${\mathcal {F}}_{{{\widetilde{V}}}}$ and ${\mathcal {F}}_{V}$ agree on $\partial {{\widetilde{V}}} \setminus \partial Q$ and similarly for the complement by localizing on *Q*, so that (9.8) holds on $\partial V\setminus \partial Q$. Similarly, by localizing on $\Omega {\setminus } {{\overline{V}}}$ and *V*, we deduce that (9.8) holds on $\partial Q \setminus \partial V$.

It remains to show that (9.8) holds on the intersection $\partial Q \cap \partial V$. Let *H* be a half-space such that $\partial H$ intersects both $\partial V$ and $\partial Q$. We assume that $H = H_{j,+}^{a_j}$ for some *j*, since the case $H = H_{j,-}^{b_j}$ is analogous. Suppose that ${{\widetilde{Q}}} \subset V$ is one of the disjoint cubes whose union gives *V*, and set

$$\begin{aligned} I := \partial Q \cap \partial {{\widetilde{Q}}} \cap \partial H \setminus ( \partial ^2Q \cup \partial ^2{{\widetilde{Q}}}). \end{aligned}$$

Since the fluxes vanish on $\partial ^2Q \cup \partial ^2{{\widetilde{Q}}}$, we know that (9.8) holds there so that, if $I = \varnothing$, there is nothing further to show. Otherwise, if $I \ne \varnothing$, observe the projections of *Q* and ${{\widetilde{Q}}}$ to $\partial H_j$ intersect non-trivially. Then it follows that ${{\widetilde{Q}}} \subset H_{j,-}^{a_j}$ since *Q* and ${{\widetilde{Q}}}$ are disjoint. Moreover, writing $Q = (a_1,b_1) \times Q'$ with $Q' \subset {\mathbb {R}}^{n-1}$ as an open cube, there is $\kappa >0$ such that $(a_1-\kappa ,a_1)\times Q' \subset {{\widetilde{Q}}}$. Therefore, setting

$$\begin{aligned} A = (a_1-\kappa ,b_1) \times Q' \end{aligned}$$

which is open, we see that $A \cap H = A \cap Q$ and $A \cap {{\overline{H}}}^{\textrm{c}} = A \cap {{\widetilde{Q}}}$. Hence, by the localization property (ii) and the two-sided property of the flux on $\partial H$ from Lemma 9.3(v), we deduce that
and, for the complement, we have
Since $I \cap \partial {{{\widetilde{V}}}} = \varnothing$, it follows that
and similarly for the complement. Thus, by inductive hypotheses with *Q* and *V*, (9.8) holds for ${{\widetilde{V}}}$ on *I*, so the result follows by induction.

Finally, given $V \in {\mathcal {V}}_{\Omega }$, by the first step, we can write *V* as a disjoint union of cubes $\{Q_i\}_{i=1}^k$ in ${\mathcal {Q}}_{\Omega }$ and, by Lemma 9.3(iv), (9.6) holds for each $Q_i$. By summation, we have

$$\begin{aligned} \sum _{i=1}^k {\mathcal {F}}_{Q_i} = \sum _{i=1}^k ({{\varvec{F}}}\cdot \nu )_{\partial Q_i} = - \sum _{i=1}^k {\mathcal {F}}_{\,\overline{Q_i}^{\textrm{c}}} = - \sum _{i=1}^k ({{\varvec{F}}}\cdot \nu )_{\partial \overline{Q_i}^{\textrm{c}}}. \end{aligned}$$

Combining this with (9.8) as proved, we obtain (9.6) as required. $\square$

#### Proof of Theorem 9.1

Let ${\mathcal {U}}_{\Omega }$ be the set of $U \Subset \Omega$ such that conclusion (9.1) holds. Then ${\mathcal {V}}_{\Omega } \subset {\mathcal {U}}_{\Omega }$ by Lemma 9.4. We fix $U \in {\mathcal {O}}_{\mu }$ and then show that $U \in {\mathcal {U}}_{\Omega }$.

To achieve this, we approximate *U* by elements in ${\mathcal {V}}_{\Omega }$ as follows: For each $k \in {\mathbb {N}}$, take a finite covering of $\overline{U^{2^{-k}}}$ by cubes $Q \in {\mathcal {Q}}_{\Omega }$ such that each $Q \Subset U^{2^{-(k+1)}}$. Let $V_k$ be the union of this covering in the sense of (9.5) so that $U^{2^{-k}} \Subset V_k \Subset U^{2^{-(k+1)}}$ and $V_k \in {\mathcal {V}}_{\Omega } \subset {\mathcal {U}}_{\Omega }$. We now divide the remaining proof into three steps.

**1**. We first show that, for any open cube $Q \Subset \Omega$ and ${\mathcal {L}}^1$–*a.e.* $\varepsilon >0$,

9.9 $$\begin{aligned} {\mathcal {F}}_{U}(Q^{\varepsilon } \cap \partial U) = \lim _{k \rightarrow \infty } {\mathcal {F}}_{V_k}(Q^{\varepsilon } \cap \partial V_k). \end{aligned}$$

To achieve this, we set

$$\begin{aligned} A_{\varepsilon ,k} := Q^{\varepsilon } \cap (U \setminus \overline{V_k}), \end{aligned}$$

and apply the balance law on this open set. By discarding a null set, we can assume that $Q^{\varepsilon } \in \widetilde{{\mathcal {O}}}_{\mu }$, so $A_{\varepsilon ,k} \in {\mathcal {O}}_{\mu }$ by Lemma 7.2. Then, by the balance law (6.17) and the additivity property (i),

9.10 $$\begin{aligned} \sigma (A_{\varepsilon ,k})&= {\mathcal {F}}_{A_{\varepsilon ,k}}(Q^{\varepsilon } \cap \partial U) +{\mathcal {F}}_{A_{\varepsilon ,k}}(Q^{\varepsilon } \cap \partial V_k)\nonumber \\&\quad \,+{\mathcal {F}}_{A_{\varepsilon ,k}}(\partial Q^{\varepsilon } \cap (U \setminus \overline{V_k}))\nonumber \\&\quad \,+{\mathcal {F}}_{A_{\varepsilon ,k}}(\partial A_{\varepsilon ,k} \cap \partial Q^{\varepsilon } \cap \partial (U \setminus \overline{V_k}))\nonumber \\&=: I_1 + I_2 + I_3 + I_4. \end{aligned}$$

Since sets $A_{\varepsilon ,k}$ are nested in *k* (as $V_{k} \subset U^{2^{-(k+1)}} \subset V_{k+1}$), we have

$$\begin{aligned} \lim _{k \rightarrow \infty } \sigma (A_{\varepsilon ,k}) = \sigma \big (Q^{\varepsilon } \cap (\cap _{k \in {\mathbb {N}}} (U \setminus \overline{V_k}))\big ) = \sigma (\varnothing )= 0 \quad \text{ for } \text{ each } \varepsilon . \end{aligned}$$

Moreover, by the localization property (ii) applied with $A=Q^{\varepsilon }$ and since each $V_k \in {\mathcal {V}}_{\Omega }$, we have

$$\begin{aligned} I_1&= {\mathcal {F}}_{U}(Q^{\varepsilon } \cap \partial U),\\ I_2&= {\mathcal {F}}_{(U \setminus \overline{V_k})}(Q^{\varepsilon } \cap \partial V_k) = - {\mathcal {F}}_{V_k}(Q^{\varepsilon } \cap \partial V_k). \end{aligned}$$

By property (ii) applied with $A =U \setminus \overline{V_k}$ and since ${\mathcal {F}}_{Q^{\varepsilon }}$ is a measure on $\partial Q^{\varepsilon }$ (by Lemma 7.1), we see that

$$\begin{aligned} \lim _{k \rightarrow \infty } I_3 = \lim _{k \rightarrow \infty } {\mathcal {F}}_{Q^{\varepsilon }}(\partial Q^{\varepsilon } \cap (U \setminus V_k)) = {\mathcal {F}}_{Q^{\varepsilon }}(\partial Q^{\varepsilon } \cap (\cap _{k \in {\mathbb {N}}} (U \setminus V_k))) = 0. \end{aligned}$$

For the final term, we set $B_{\varepsilon ,k}:= \partial A_{\varepsilon ,k} \cap \partial Q^{\varepsilon } \cap \partial (U \setminus \overline{V_k})$ and claim that

9.11 $$\begin{aligned} \mu _{A_{\varepsilon ,k}}^{n-1}(B_{\varepsilon ,k}) = 0 \quad \text{ for } \text{ any } k \text{ and } {\mathcal {L}}^1\textit{--a.e.}\,\,\varepsilon >0. \end{aligned}$$

Indeed, let $\delta _j \searrow 0$ such that
Then, by arguing as in the proof of Lemma 7.2, we see that $\frac{1}{\delta _j} \mu (U \setminus \overline{U^{\delta _j}})$ is uniformly bounded in *j* and hence, passing to a subsequence (depending on $\varepsilon$ and *k*), we obtain a limiting measure
Since ${\overline{V}}_k^{\textrm{c}}$, $Q^{\varepsilon } \in \widetilde{{\mathcal {O}}}_{\mu }$, passing to a further subsequence, we can also assume that there exist limit measures:
By Lemma 7.3(i),

9.12 $$\begin{aligned} \mu _{A_{\varepsilon ,k}}^{n-1}(B_{\varepsilon ,k}) \le \mu _{U}^{n-1}(\partial Q^{\varepsilon } \cap \partial U) + \mu _{\,\overline{V_k}^{\textrm{c}}}^{n-1}(\partial Q^{\varepsilon } \cap \partial V_k) + \mu _{Q^{\varepsilon }}^{n-1}(B_{\varepsilon ,k}) \end{aligned}$$

for each *k* and ${\mathcal {L}}^1$–*a.e.* $\varepsilon >0$. We now show that (9.12) vanishes for any *k* and ${\mathcal {L}}^1$–*a.e.* $\varepsilon >0$.

Indeed, by considering  and noting that $\mu _{A_{\varepsilon ,k}}^{n-1} = {{\widetilde{\mu }}}_{A_{\varepsilon ,k}}^{n-1}$ for all $\varepsilon$ and *k*, we can assume that $\mu (\partial U) = 0$ in what follows. Since $\{\partial Q^{\varepsilon }\}_{\varepsilon >0}$ are pairwise disjoint in $\varepsilon >0$, for all but countably many $\varepsilon >0$, we have

$$\begin{aligned} \mu ^{n-1}_{U}(\partial Q^{\varepsilon } \cap \partial U) + \mu ^{n-1}_{\overline{V_k}^{\textrm{c}}}(\partial Q^{\varepsilon } \cap \partial V_k) = 0 \quad \text {for any }k. \end{aligned}$$

Also, by disintegration along $\partial Q^{\varepsilon }$, we obtain

$$\begin{aligned} \int _0^{\infty }\mu ^{n-1}_{Q^{\varepsilon }}(\partial Q^{\varepsilon } \cap (\partial U \cap \partial V_k)) \,\textrm{d}\varepsilon \le \mu (Q \setminus Q^{\varepsilon _0} \cap (\partial U \cap \partial V_k)) = 0, \end{aligned}$$

by noting that $\mu (\partial U) = 0$ as assumed and $\mu (\partial V_k) = 0$ by Lemma 9.3(vi). Then, for ${\mathcal {L}}^1$–*a.e.* $\varepsilon >0$, $\mu ^{n-1}_{Q^{\varepsilon }}(B_{\varepsilon ,k}) = 0$ for all *k* which, combining with (9.12), leads to (9.11) as claimed. Hence, by property (iii), we have

$$\begin{aligned} |I_4|= |{\mathcal {F}}_{A_{\varepsilon ,k}}(B_{\varepsilon ,k})|\le \mu ^{n-1}_{A_{\varepsilon ,k}}(B_{\varepsilon ,k}) = 0, \end{aligned}$$

giving $I_4 \rightarrow 0$ for this choice of $\varepsilon$. Thus, combining everything in (9.10), we conclude

$$\begin{aligned} \begin{aligned} 0 = \lim _{k \rightarrow \infty } \sigma (A_{\varepsilon ,k})&= I_1 + \lim _{k \rightarrow \infty } I_2 + \lim _{k \rightarrow \infty } (I_3+I_4) \\&= {\mathcal {F}}_{U}(Q^{\varepsilon } \cap \partial U) - \lim _{k \rightarrow \infty } {\mathcal {F}}_{V_k}(Q^{\varepsilon } \cap \partial V_k), \end{aligned} \end{aligned}$$

which rearranges to give (9.9) as claimed.

**2**. Notice that the normal trace ${{\varvec{F}}}\cdot \nu$ defines a Cauchy flux $\widetilde{{\mathcal {F}}}$ with the same $\mu$ and $\sigma$ by Theorem 6.7. By restricting the allowed parameter $\varepsilon >0$ if necessary, we can ensure that the above claim also holds for $\widetilde{{\mathcal {F}}}$, that is,

$$\begin{aligned} ({{\varvec{F}}}\cdot \nu )_{\partial U}(Q^{\varepsilon } \cap \partial U) = \lim _{k \rightarrow \infty } ({{\varvec{F}}}\cdot \nu )_{\partial V_k}(Q^{\varepsilon } \cap \partial V_k). \end{aligned}$$

Since $$V_k \in {\mathcal {V}}_{\Omega } \subset {\mathcal {U}}_{\Omega }$$, then

9.13 $$\begin{aligned} {\mathcal {F}}_{U}(Q^{\varepsilon } \cap \partial U) = ({{\varvec{F}}}\cdot \nu )_{\partial U}(Q^{\varepsilon } \cap \partial U) \end{aligned}$$

for all cubes $Q \Subset \Omega$ and for ${\mathcal {L}}^1$–*a.e.* $\varepsilon >0$, where the null set depends on *Q*. We can further assume that
9.14by restricting the allowed parameter $\varepsilon >0$.

**3**. To complete the proof, we need to extend (9.13) to hold for all Borel subsets of $\partial U$. To do this, for each $\varepsilon >0$, define

9.15 $$\begin{aligned} {\mathcal {D}}_{\Omega }^{\varepsilon } = \big \{ Q(a,b)^{\varepsilon }\, :\, a,b \in {\mathbb {Q}}^n,\, Q(a,b) \Subset \Omega \big \}, \end{aligned}$$

and let $\widetilde{{\mathcal {D}}}_{\Omega }^{\varepsilon }$ be the collection of all finite unions of cubes in ${\mathcal {D}}_{\Omega }^{\varepsilon }$ in the sense of (9.5). Since this is a countable collection, for ${\mathcal {L}}^1$–*a.e.* $\varepsilon >0$, we can ensure that (9.13)–(9.14) hold for each $Q^{\varepsilon } \in {\mathcal {D}}_{\Omega }^{\varepsilon }$.

We first claim that ${\mathcal {D}}_{\Omega }^{\varepsilon }$ is closed under finite intersections. Indeed, observe that

$$\begin{aligned} Q(a,b)^{\varepsilon } = (a_1+\varepsilon ,b_1-\varepsilon ) \times \cdots \times (a_n + \varepsilon , b_n - \varepsilon ) = Q(a+\varepsilon \pmb {1},b-\varepsilon \pmb {1}), \end{aligned}$$

where $\pmb {1} = (1,\cdots ,1) \in {\mathbb {R}}^n$. Using this, we can verify that $(Q_1 \cap Q_2)^{\varepsilon } = Q_1^{\varepsilon } \cap Q_2^{\varepsilon }$ in general, from which the assertion follows. Now, let $V \in \widetilde{{\mathcal {D}}}_{\Omega }^{\varepsilon }$, which can be written as a union $V = \text {int}(\bigcup _{i=1}^k {{\overline{Q}}}_i)$, where $Q_i \in {\mathcal {D}}_{\Omega }^{\varepsilon }, i=1,\cdots , k$, are pairwise disjoint. Then

$$\begin{aligned} {\mathcal {F}}_U(V \cap \partial U) = \sum _{i=1}^k {\mathcal {F}}_U(Q_i \cap \partial U) = \sum _{i=1}^k ({{\varvec{F}}}\cdot \nu )_{\partial U}(Q_i \cap \partial U) = ({{\varvec{F}}}\cdot \nu )_{\partial U}(V \cap \partial U) \end{aligned}$$

by using (9.13)–(9.14). Thus, (9.14) holds if $Q^{\varepsilon }$ is replaced by the elements in $\widetilde{{\mathcal {D}}}_{\Omega }^{\varepsilon }$.

Now, let $A \subset \Omega ^{\varepsilon }$ be open. We claim that, for each $x \in A$, there is a cube in $Q^{\varepsilon } \in {\mathcal {D}}_{\Omega }^{\varepsilon }$ such that $x \in Q^{\varepsilon } \Subset A$. Indeed, there exists $\delta >0$ such that $Q_0:= Q(x-\delta \pmb {1},x+\delta \pmb {1}) \Subset A$. Then we can choose $y \in {\mathbb {Q}}^n$ so that $|x- y|< \frac{\delta }{4}$ and $r \in {\mathbb {Q}} \cap (\varepsilon +\frac{\delta }{4},\varepsilon +\frac{\delta }{2})$. Letting $Q_1 = Q(y-r\pmb {1},y+r\pmb {1})$, we see that $Q_1^{\varepsilon } \in {\mathcal {D}}_{\Omega }^{\varepsilon }$. Since $\frac{\delta }{4}< r - \varepsilon < \frac{\delta }{2}$, we obtain the following inclusions:

$$\begin{aligned} x \in Q_1^{\varepsilon } = Q(y-(r-\varepsilon )\pmb {1},y+(r-\varepsilon )\pmb {1}) \Subset Q_0 \Subset A, \end{aligned}$$

as required. Performing this about each point and passing to a countable subcover give a collection $\{Q_j\} \subset {\mathcal {D}}_{\Omega }^{\varepsilon }$ such that $\bigcup _j Q_j = A$. Then, since (9.13) holds for elements in $\widetilde{{\mathcal {D}}}_{\Omega }^{\varepsilon }$, it follows that

$$\begin{aligned} {\mathcal {F}}_U\big (\bigcup _{j=1}^J Q_j \cap \partial U\big ) = ({{\varvec{F}}}\cdot \nu )_{\partial U}\big (\bigcup _{j=1}^J Q_j \cap \partial U\big ) \quad \text {for any } J, \end{aligned}$$

so passing to the limit gives

9.16 $$\begin{aligned} {\mathcal {F}}_U(A \cap \partial U) = ({{\varvec{F}}}\cdot \nu )_{\partial U}(A \cap \partial U) \end{aligned}$$

for any $A \subset \Omega ^{\varepsilon }$ open and ${\mathcal {L}}^1$–*a.e.* $\varepsilon >0$. Since both sides are Radon measures and $\varepsilon >0$ can be chosen to be arbitrarily small, it follows from Lemma 2.2 that this holds when *A* is replaced by an arbitrary Borel set $B \subset \Omega$. From this, we infer that $U \in {\mathcal {U}}_{\Omega }$, establishing the result. $\square$

We can now collect the results established in the previous results to prove the main theorem.

#### Proof of Theorem 6.6

Given a Cauchy flux ${\mathcal {F}}$, Theorem 8.1 gives the existence of a measure-valued field ${{\varvec{F}}}$, which further satisfies the global and local recovery properties by Theorems 8.2 and 9.1, respectively. Moreover, Theorem 8.4 shows that this flux is unique and the converse statement is precisely the content of Theorem 6.7. $\square$

### Consequences of the Main Theorem

Using the equivalence given by Theorem 6.6, we can infer the properties of the Cauchy flux based on the results about the normal traces established in earlier sections. We now list two of such consequences.

#### Theorem 9.5

Let ${\mathcal {F}}$ be a Cauchy flux, and let $U \Subset \Omega$ such that $U, {\overline{U}}^{\textrm{c}} \in {\mathcal {O}}_{\mu }$ and $\mu (\partial U) = |\sigma |(\partial U) = 0$. Then

$$\begin{aligned} {\mathcal {F}}_U(S) = - {\mathcal {F}}_{\,{{\overline{U}}}^{\textrm{c}}}(S) \quad \text {for all Borel subsets } S \subset \partial U, \end{aligned}$$

where ${\mathcal {F}}_{\,{{\overline{U}}}^{\textrm{c}}}$ is defined through Remark 7.7.

#### Proof

  Let ${{\varvec{F}}}$ be the corresponding $\mathcal{D}\mathcal{M}^{{{\,\textrm{ext}\,}}}$–field given by Theorem 6.6. Then the corresponding result for the normal traces holds by Remark 2.12, which asserts that

9.17 $$\begin{aligned} \langle {{\varvec{F}}}\cdot \nu ,\, \cdot \,\rangle _{\partial U} = \langle {{\varvec{F}}}\cdot \nu ,\, \cdot \, \rangle _{\partial {{\overline{U}}}} \end{aligned}$$

as distributions, whenever $|{{\varvec{F}}}|(\partial U) = |\operatorname {div}{{\varvec{F}}}|(\partial U) = 0$, and this is satisfied since $|{{\varvec{F}}}|\ll \mu$ and $|\operatorname {div}{{\varvec{F}}}|\ll |\sigma |$. Since $U \in {\mathcal {O}}_{\mu }$, both sides of (9.17) can be represented as measures, which remain equal. By Theorem 6.6, ${\mathcal {F}}_{U}(\cdot ) = \langle {{\varvec{F}}}\cdot \nu , \,\cdot \,\rangle _{\partial U}$ as measures. On the other hand, since ${{\overline{U}}}^{\textrm{c}} \in {\mathcal {O}}_{\mu }$, using the notation from Remark 7.7, for ${\mathcal {L}}^1$–*a.e.* $\delta >0$ such that $U \Subset {{\tilde{\Omega }}}^{\delta }$, we have

$$\begin{aligned} \langle {{\varvec{F}}}\cdot \nu , \,\cdot \,\rangle _{\partial ( {{\tilde{\Omega }}}^{\delta }\setminus {{\overline{U}}})} = \langle {{\varvec{F}}}\cdot \nu ,\,\cdot \,\rangle _{\partial {{\tilde{\Omega }}}^{\delta }} - \langle {{\varvec{F}}}\cdot \nu , \,\cdot \,\rangle _{\partial {{\overline{U}}}}. \end{aligned}$$

Restricting to ${{\tilde{\Omega }}}^{\delta }$ by using Theorem 5.3 and Remark 7.7, we obtain
We combine this with (9.17) to complete the proof. $\square$

#### Theorem 9.6

For any open set $U \Subset \Omega$,

9.18 $$\begin{aligned} \lim _{\varepsilon \rightarrow 0} {\mathcal {F}}_{U^{\varepsilon }}(\partial U^{\varepsilon }) = {\mathcal {F}}_U(\partial U). \end{aligned}$$

If, in addition, $U \in {\mathcal {O}}_{\mu }$, then there exists $t_k \rightarrow 0$ such that each $U^{t_k} \in {\mathcal {O}}_{\mu }$, and


#### Proof

  The global convergence of the flux sequence in (9.18) is immediate from the balance law (6.17), since $\bigcup _{\varepsilon >0} U^{\varepsilon } = U$. For the local convergence, since $U \in {\mathcal {O}}_{\mu }$, there is $\varepsilon _k \searrow 0$ such that

$$\begin{aligned} M := \sup _{k \in {\mathbb {N}}} \frac{1}{\varepsilon _k} \,\mu (U \setminus {\overline{U}}^{\varepsilon _k}) < \infty . \end{aligned}$$

By Lemma 7.4, we can disintegrate $\mu$ along $\partial U^t$ as
Consider the function:

$$\begin{aligned} T(t) = |\mu _{U^t}^{n-1} |(\partial U^t) \end{aligned}$$

defined ${\mathcal {L}}^1$–*a.e.* $t>0$ such that (7.4) holds, which also holds for any *t* outside a null set ${\mathcal {N}} \subset [0,\infty )$. We set $T(t) = 0$ whenever $t \in {\mathcal {N}}$. Then $T \in L^1([0,\infty ))$ and

$$\begin{aligned} \frac{1}{\varepsilon _k} \int _0^{\varepsilon _k} T(t) \,\textrm{d}t \le M \quad \text{ for } \text{ any } k. \end{aligned}$$

Applying the Markov inequality gives

$$\begin{aligned} {\mathcal {L}}^1(\{t \in (0,\varepsilon _k) \setminus {\mathcal {N}}_k : T(t) \ge 2M\}) \le \frac{1}{2M} \int _0^{\varepsilon _k} T(t) \,\textrm{d}t \le \frac{\varepsilon _k}{2}, \end{aligned}$$

so that there exists a sequence $t_k \in (0,\varepsilon _k)$ of distinct values such that $T(t_k) \le 2\,M$ for all *k*. Then $t_k \rightarrow 0$ and

$$\begin{aligned} |{\mathcal {F}}_{U^{t_k}} |(\partial U^{t_k}) \le \eta _{U^{t_k}}(\partial U^{t_k}) = T(t_k) \le 2M \quad \text{ for } \text{ any } k. \end{aligned}$$

Passing to a subsequence, we see that ${\mathcal {F}}_{U^{t_k}}$ converges weakly ${}^*$ in $\Omega$.

Now, by Theorem 6.6, ${\mathcal {F}}$ is represented by the normal trace of a field ${{\varvec{F}}}\in \mathcal{D}\mathcal{M}^{{{\,\textrm{ext}\,}}}(\Omega )$, so that the weak ${}^{*}$–limit of these measures can be identified with ${\mathcal {F}}_U = ({{\varvec{F}}}\cdot \nu )_{\partial U}$ by Theorem 3.3. $\square$

## Extension of the Normal Trace

So far, we have restricted our attention to the normal trace on the boundary of a set *E* that is compactly contained in $\Omega$. However, in applications, it is useful to consider a set $E \subset \Omega$ whose boundary may touch $\partial \Omega$, for which we introduce the following definition:

### Definition 10.1

  Let $\Omega \subset {\mathbb {R}}^n$ be open and ${{\varvec{F}}}\in \mathcal{D}\mathcal{M}^{{{\,\textrm{ext}\,}}}(\Omega )$. For a Borel set $E \subset \Omega$, define the *normal trace* of ${{\varvec{F}}}$ on the boundary of *E* as

$$\begin{aligned} \langle {{\varvec{F}}}\cdot \nu , \,\phi \rangle _{\partial E} = - \int _{E} \textrm{d}\overline{\nabla \phi \cdot {{\varvec{F}}}} - \int _E \phi \,\textrm{d}(\operatorname {div}{{\varvec{F}}}) \quad \text {for any } \phi \in {W}^{1,\infty }(\Omega ), \end{aligned}$$

where the middle term is defined from the product rule (Theorem 2.7).

If $E \Subset \Omega$, then this coincides with Definition 2.4 when testing against $\phi \in C^1_{\textrm{c}}(\Omega )$, and it is precisely how we extended the normal trace to ${W}^{1,\infty }(\Omega )$ in Corollary 2.9. We do not require that $\phi$ vanishes on $\partial \Omega$, since $\partial E \cap \partial \Omega$ may be non-empty in general. We can also generalize (2.11) as

10.1 $$\begin{aligned} \langle {{\varvec{F}}}\cdot \nu , \,\mathbbm {1}_{\Omega }\rangle _{\partial U} = (\operatorname {div}{{\varvec{F}}})(U) \quad \text {for any } U \subset \Omega . \end{aligned}$$

We record that the normal trace remains supported on $\partial U$ in the sense of Theorem 2.15.

### Lemma 10.2

Let $U \subset \Omega$ be open, ${{\varvec{F}}}\in \mathcal{D}\mathcal{M}^{\textrm{ext}}(\Omega )$, and let $\phi \in \textrm{Lip}_{\textrm{b}}(\Omega )$ vanish on $\partial U$. Then

$$\begin{aligned} \langle {{\varvec{F}}}\cdot \nu , \,\phi \rangle _{\partial U} = 0. \end{aligned}$$

### Proof

  Since the argument is similar to the proof of Theorem 2.15, we only outline the main modifications. Observe that, since any function $\phi \in \textrm{Lip}_{\textrm{b}}(\Omega )$ admits a unique continuous extension to $\partial \Omega$, the condition: $\phi |_{\partial U} = 0$ is well-posed.

Given $0< \delta < 1$, let $d_{\delta }$ as in (2.14) with *U* in place of *E* so that $d_{\delta }$ is supported in $\Omega ^{\delta }$. Also, let $\chi _{\delta } \in {W}^{1,\infty }({\mathbb {R}}^n)$ be 1-Lipschitz such that $\chi _{\delta } \equiv 1$ in $B_{1/(2\delta )}$ with support in $B_{1/\delta }$. Thus, letting $\psi _{\delta }:= \chi _{\delta }d_{\delta }$, we see that $\psi _{\delta }$ is $\frac{1}{\delta }$-Lipschitz and is supported on ${{\widetilde{\Omega }}}^{\delta } = B_{1/\delta } \cap \Omega ^{\delta } \Subset \Omega$.

Then, by Theorem 2.15 with $\psi _{\delta }\phi$ on $U \cap {{\widetilde{\Omega }}}^{\delta }$, we have

$$\begin{aligned} \begin{aligned} 0&= \langle {{\varvec{F}}}\cdot \nu , \,\psi _{\delta }\phi \rangle _{\partial (U \cap {{\widetilde{\Omega }}}^{\delta })}\\&= \int _{U \cap {{\widetilde{\Omega }}}^{\delta }} \psi _{\delta }\phi \,\textrm{d}(\operatorname {div}{{\varvec{F}}}) + \int _{U \cap {{\widetilde{\Omega }}}^{\delta }} \textrm{d}\overline{\nabla (\psi _{\delta }\phi ) \cdot {{\varvec{F}}}} \\&= \int _{U} \psi _{\delta }\phi \,\textrm{d}(\operatorname {div}{{\varvec{F}}}) + \int _{U}\textrm{d}\overline{\nabla (\psi _{\delta }\phi ) \cdot {{\varvec{F}}}} \\&= \langle {{\varvec{F}}}\cdot \nu , \,\psi _{\delta }\phi \rangle _{\partial U}, \end{aligned} \end{aligned}$$

by noting that both integrals vanish outside ${{\widetilde{\Omega }}}^{\delta }$.

We now pass to the limit in $\delta \searrow 0$. Using the definition of the pairing from Theorem 2.7,

$$\begin{aligned} \overline{\nabla (\psi _{\delta }\phi )\cdot {{\varvec{F}}}} = \psi _{\delta }\,\overline{\nabla \phi \cdot {{\varvec{F}}}} + \phi \,\overline{\nabla \psi _{\delta } \cdot {{\varvec{F}}}}, \end{aligned}$$

and since $\nabla \psi _{\delta }$ is supported on $A_{\delta } = {{\widetilde{\Omega }}}^{\delta } \setminus \Omega ^{2\delta }$ and $|\phi |\le 2 \delta \Vert \nabla \phi \Vert _{L^{\infty }(\Omega )}$ on $A_{\delta }$, analogously to (2.16), we obtain

$$\begin{aligned} \Big |\int _U \phi \,\textrm{d}\overline{\nabla \psi _{\delta }\cdot {{\varvec{F}}}} \Big |\le 2 \Vert \nabla \phi \Vert _{L^{\infty }(\Omega )} |{{\varvec{F}}}|(A_{\delta }), \end{aligned}$$

which vanishes as $\delta \rightarrow 0$. Finally, since $\psi _{\delta }(x) \rightarrow 1$ for any $x \in U$, by the Dominated Convergence Theorem and the above bounds, we have

$$\begin{aligned} 0 = \lim _{\delta \rightarrow 0} \langle {{\varvec{F}}}\cdot \nu , \,\psi _{\delta }\phi \rangle _{\partial U} = \langle {{\varvec{F}}}\cdot \nu , \,\phi \rangle _{\partial U} \end{aligned}$$

as required. $$\square $$

Equipped with this result, we can also argue as in Corollary 2.17 and view the normal trace on an open set $U \subset \Omega$ as a linear functional $N_U \in \textrm{Lip}_{\textrm{b}}(\partial U)^{*}$.

### Remark 10.3

  While the corresponding result for $U \Subset \Omega$ (Theorem 2.15) is stated for any function $\phi \in {W}^{1,\infty }(\Omega )$, we have stated Lemma 10.2 for the test function in $\textrm{Lip}_{\textrm{b}}(\Omega )$. This is because, for a general function $\phi \in {W}^{1,\infty }(\Omega )$, we only know that $\phi$ is continuous in $\Omega$, which means that the condition: $\phi |_{\partial U} = 0$ may be ill-posed if $\partial U \not \subset \Omega$. However, if we take $\phi \in {W}^{1,\infty }(\Omega )$ and additionally impose that

$$\begin{aligned} |\phi (x) |\le C \delta \quad \text {for any } x \in \partial U^{\delta } \end{aligned}$$

for some $C>0$ and all $\delta > 0$, then we can argue analogously as in the above proof.

We can also extend Theorems 3.3, 4.1, and 4.4 to hold for general $U \subset \Omega$.

### Theorem 10.4

Let ${{\varvec{F}}}\in \mathcal{D}\mathcal{M}^{{{\,\textrm{ext}\,}}}(\Omega )$, and let $U \subset \Omega$ be open and $d(x):= \textrm{dist}(x,\partial U)$. Then there exists an ${\mathcal {L}}^1$-null set ${\mathcal {N}} \subset (0,\infty )$ such that

(i) For any $\varepsilon \notin {\mathcal {N}}$, the normal trace $\langle {{\varvec{F}}}\cdot \nu , \,\cdot \,\rangle _{\partial U^{\varepsilon }}$ is represented by a measure on $\partial U^{\varepsilon }$, denoted by $({{\varvec{F}}}\cdot \nu )_{\partial U^{\varepsilon }}$.
(ii) For any $\phi \in {W}^{1,\infty }(\Omega )$ and any sequence $\varepsilon _k \rightarrow 0$ with $\varepsilon _k \notin {\mathcal {N}}$,
  $$\begin{aligned} \langle {{\varvec{F}}}\cdot \nu , \,\phi \rangle _{\partial U} = \lim _{k \rightarrow \infty } \int _{\partial U^{\varepsilon _k}} \phi \,\textrm{d}({{\varvec{F}}}\cdot \nu )_{\partial U^{\varepsilon _k}}. \end{aligned}$$
(iii) For any $\phi \in {W}^{1,\infty }(\Omega )$,
  $$\begin{aligned} \langle {{\varvec{F}}}\cdot \nu , \,\phi \rangle _{\partial U} = \lim _{\varepsilon \rightarrow 0} \frac{1}{\varepsilon } \int _{U \setminus \overline{U^{\varepsilon }}} \phi \,\textrm{d}\overline{\nabla d \cdot {{\varvec{F}}}}. \end{aligned}$$
(iv) For any bounded Borel function $\phi$ on $\Omega$,
  $$\begin{aligned} \int _0^{\infty } \int _{\partial U^t} \phi \,\textrm{d}({{\varvec{F}}}\cdot \nu )_{\partial U^t} \,\textrm{d}t = \int _U \phi \,\textrm{d}\overline{\nabla d \cdot {{\varvec{F}}}}, \end{aligned}$$
  understanding that the integrand is defined for $t \in (0,\infty ) \setminus {\mathcal {N}}$.

### Proof

  For $0< t < s$, define $\psi ^U_{t,s}$ by (3.4), which lies in $\textrm{Lip}_{\textrm{b}}(\Omega ) \subset {W}^{1,\infty }(\Omega )$ and vanishes on $\partial U$. Choose *s* and *t* such that $|\overline{\nabla d\cdot {{\varvec{F}}}}|(\partial U^t) =|\overline{\nabla d\cdot {{\varvec{F}}}}|(\partial U^s) = 0$, which holds for all but countably many *s* and *t*. By Lemma 10.2 and the product rule (Theorem 2.7), we have

$$\begin{aligned} \begin{aligned} 0&= \langle {{\varvec{F}}}\cdot \nu , \,\psi _{t,s}^U\phi \rangle _{\partial U} \\&= \int _U \psi _{s,t}^U \,\textrm{d}(\operatorname {div}(\phi {{\varvec{F}}})) + \int _U \textrm{d}\overline{\nabla \psi _{t,s}^U \cdot (\phi {{\varvec{F}}})} \\&= \int _U \psi _{t,s}^U \,\textrm{d}(\operatorname {div}(\phi {{\varvec{F}}})) + \int _{U^t \setminus \overline{U^s}} \phi \,\textrm{d}\overline{\nabla d \cdot {{\varvec{F}}}}. \end{aligned} \end{aligned}$$

We can then argue as in the proof of Theorem 3.3 to show that the disintegration of $\overline{\nabla d \cdot {{\varvec{F}}}}$ takes the form:

$$\begin{aligned} \overline{\nabla d \cdot {{\varvec{F}}}} = {\mathcal {L}}^1 \otimes _{\partial U^t} ({{\varvec{F}}}\cdot \nu )_{\partial U^t} + \tau _{\textrm{sing}} \otimes _{\partial U^t} \mu _t, \end{aligned}$$

from which (i) and (ii) follow. For (iv), we can argue as in the proof of Theorem 4.1 to show that $\tau _{\textrm{sing}}=0$, by observing the test function $\phi (x) = \psi (d(x)) g(x)$ remains compactly supported when $U \subset \Omega$. Finally, (iii) follows from (iv) by arguing exactly as in the proof of Theorem 4.4. $\square$

The localization result from Sect. 5 also extends to this setting as follows:

### Theorem 10.5

Let $\Omega \subset {\mathbb {R}}^n$ be open and ${{\varvec{F}}}\in \mathcal{D}\mathcal{M}^{{{\,\textrm{ext}\,}}}(\Omega )$. For $U, V \subset \Omega$, suppose that an open set $A \subset {\mathbb {R}}^n$ satisfies

$$\begin{aligned} U \cap A = V \cap A. \end{aligned}$$

Then

$$\begin{aligned} \langle {{\varvec{F}}}\cdot \nu , \,\phi \rangle _{\partial U} = \langle {{\varvec{F}}}\cdot \nu , \,\phi \rangle _{\partial V} \quad \text {for any } \phi \in {W}^{1,\infty }_{\textrm{c}}(A). \end{aligned}$$

As in Theorem 5.3, if the normal traces on $\partial U$ and $\partial V$ are represented by measures, then a density argument implies that
as measures. The proof of Theorem 10.5 is analogous to that of Theorem 5.3, by noting that *A* can be replaced by a bounded open set ${\tilde{A}}$ containing $A \cap \operatorname {spt}(\phi )$, since $\phi$ is compactly supported in *A*, and that Theorem 10.4(iii) can be applied in place of Theorem 4.4.

Finally, we can formulate the Cauchy flux to be defined up to the boundary of $\Omega$. Given a non-negative measure $\mu$ on $\Omega$, we introduce the set

$$\begin{aligned} \overline{{\mathcal {O}}}_{\mu } = \Big \{ U \subset \Omega \text { open}\,:\,\liminf _{\varepsilon \rightarrow 0}\frac{1}{\varepsilon }\, \mu (U \setminus \overline{U^{\varepsilon }}) < \infty \Big \}. \end{aligned}$$

### Definition 10.6

  An *extended Cauchy flux* in $\Omega$ is a mapping ${\mathcal {F}}$ defined on pairs (*S*, *U*) with $S \subset \partial U$ Borel and $U \subset \Omega$ open, which satisfies the balance law (6.17) for any $U \subset \Omega$ so that there is a Radon measure $\mu$ such that properties (i)–(iii) hold with ${\mathcal {O}}_{\mu }$ replaced by $\overline{{\mathcal {O}}}_{\mu }$.

### Theorem 10.7

Let ${\mathcal {F}}$ be an extended Cauchy flux in $\Omega$. Then there exists a unique divergence-measure field ${{\varvec{F}}}\in \mathcal{D}\mathcal{M}^{{{\,\textrm{ext}\,}}}(\Omega )$ representing ${\mathcal {F}}$ in the sense that both the global recovery:

10.2 $$\begin{aligned} {\mathcal {F}}_{U}(\partial U) = \langle {{\varvec{F}}}\cdot \nu , \,\mathbbm {1}_{\Omega } \rangle _{\partial U} \quad \text {for any open set } U \subset \Omega , \end{aligned}$$

and the local recovery:

10.3 $$\begin{aligned} {\mathcal {F}}_U(S) = (F \cdot \nu )_{\partial U}(S) \quad \text{ for } \text{ any } U \in \overline{{\mathcal {O}}}_{\mu } \text{ and } \text{ any } \text{ Borel } \text{ Set } S \subset \partial U \end{aligned}$$

hold. Conversely, any ${{\varvec{F}}}\in \mathcal{D}\mathcal{M}^{{{\,\textrm{ext}\,}}}(\Omega )$ defines an extended Cauchy flux by (10.2)–(10.3), and every Cauchy flux ${\mathcal {F}}$ in the sense of Definition 6.4 extends uniquely to an extended Cauchy flux.

### Proof

Since an extended Cauchy flux ${\mathcal {F}}$ is also a Cauchy flux in the sense of Definition 6.4, the existence and uniqueness of a representing field ${{\varvec{F}}}\in \mathcal{D}\mathcal{M}^{{{\,\textrm{ext}\,}}}(\Omega )$ satisfying $-\operatorname {div}{{\varvec{F}}}= \sigma$ follows from Theorems 8.1–8.2 and 8.4. Then, from (10.1), we obtain (10.2).

It remains to extend the local recovery result (Theorem 9.1) to hold for any $U \in \overline{{\mathcal {O}}}_{\mu }$. For this, we closely follow the proof of Theorem 9.1, approximating $U \in \overline{{\mathcal {O}}}_{\mu }$ by subsets $V_k \in {\mathcal {V}}_{\Omega }$; since each $V_k \Subset \Omega$, the localization and two-sided properties are guaranteed by Lemma 9.4. The key difference is to show that (9.9) holds for all cubes $Q \subset {\mathbb {R}}^n$ and ${\mathcal {L}}^1$–*a.e.* $\varepsilon >0$, regardless of whether $Q^{\varepsilon }$ is contained in $\Omega$. This is possible because $A_{\varepsilon ,k} = Q^{\varepsilon } \cap (U \setminus \overline{V_k}) \subset \Omega$, so that the balance law (6.17) can be applied in this extended setting. We choose $\varepsilon >0$ such that

$$\begin{aligned} \limsup _{\delta \rightarrow 0} \frac{1}{\delta } \, \mu \big (\Omega \cap (Q^{\varepsilon } \setminus Q^{\varepsilon +\delta })\big ) < \infty , \end{aligned}$$

which is valid for ${\mathcal {L}}^1$–*a.e.* $\varepsilon >0$ by Lemma 7.4, extending $\mu$ by zero to $\mathbb R^n$. Then we can argue analogously as in the proof of Theorem 9.1 (especially (9.9)) that

$$\begin{aligned} {\mathcal {F}}_U(Q^{\varepsilon } \cap \partial U) = \lim _{k \rightarrow \infty } {\mathcal {F}}_{V_k}(Q^{\varepsilon } \cap \partial V_k) \quad \text{ for } \text{ any } \text{ such } Q^{\varepsilon }. \end{aligned}$$

We then consider the collection of cubes ${\mathcal {D}}_{{\mathbb {R}}^n}^{\varepsilon }$ as in (9.15) and observe that, for ${\mathcal {L}}^1$–*a.e.* $\varepsilon >0$, both (9.13)–(9.14) hold for each $Q \in {\mathcal {D}}_{{\mathbb {R}}^n}^{\varepsilon }$. Then, covering $A \subset {\mathbb {R}}^n$ by cubes in ${\mathcal {D}}_{{\mathbb {R}}^n}^{\varepsilon }$, we can infer that the local recovery (9.16) holds for any open set *A*, from which a density argument extends the result to all Borel subsets.

Finally, the fact that a flux ${{\varvec{F}}}\in \mathcal{D}\mathcal{M}^{{{\,\textrm{ext}\,}}}(\Omega )$ defines an extended Cauchy flux follows analogously as in the proof of Theorem 6.7, by using (10.1) and Theorems 10.4–10.5 if necessary. Also, if ${\mathcal {F}}$ is a Cauchy flux, letting ${{\varvec{F}}}\in \mathcal{D}\mathcal{M}^{{{\,\textrm{ext}\,}}}(\Omega )$ be the associated field guaranteed by Theorem 6.6, the extended Cauchy flux $\widetilde{{\mathcal {F}}}$ defined via the normal trace of ${{\varvec{F}}}$ is the unique extension of ${\mathcal {F}}$. $\square$

## Remarks on the Existence of Divergence-Measure Fields

The characterization of the solvability of the equation with a Radon measure $\sigma$:

11.1 $$\begin{aligned} -\operatorname {div}{{\varvec{F}}}= \sigma \end{aligned}$$

has been obtained in Phuc-Torres [53, 54] in several spaces of functions, including continuous vector fields and vector fields in $L^{p}$, $1 \le p \le \infty$.

The next theorem provides a way to show the existence of a solution of (11.1) that is a vector-valued measure, that is, an extended divergence-measure field.

### Theorem 11.1

Given a finite signed Radon measure $\sigma$ in a bounded open set $\Omega \subset {\mathbb {R}}^{n}$, there exists a vector-valued measure ${{\varvec{F}}}=(F_1,\cdots ,F_n) \in {\mathcal {M}}(\Omega ;{\mathbb {R}}^n)$ such that $-\operatorname {div}{{\varvec{F}}}=\sigma$ in the sense of distributions.

### Proof

  Let $C_{\textrm{c}}(\Omega )$ be the vector space of all real continuous functions with compact support defined in $\Omega$, equipped with the norm:

$$\begin{aligned} \left\Vert u\right\Vert _{C_0(\Omega )} = \sup _{x \in \Omega }\,|u(x) |. \end{aligned}$$

Let $C_0(\Omega )$ denote the completion of $C_{\textrm{c}}(\Omega )$ in this norm. Similarly, let $C_{\textrm{c}}^{1}(\Omega )$ be the vector space of all differentiable functions with compact support in $\Omega$ such that the derivatives are also continuous in $\Omega$, which is equipped with the norm:

$$\begin{aligned} \left\Vert u\right\Vert _{C_0^1(\Omega )} = \sup _{x \in \Omega }\,|u(x)|+ \sup _{x \in \Omega }\,|\nabla u(x)|. \end{aligned}$$

Let $C_0^{1}(\Omega )$ denote the completion of $C_{\textrm{c}}^{1}(\Omega )$ with this norm. Observe that $\sigma \in C_0^1(\Omega )^{*}$, since we can estimate

11.2 $$\begin{aligned} \Big |\int _{\Omega } \varphi \,\textrm{d}\sigma \Big |\le |\sigma |(\Omega )\,\sup _{\Omega }\, |\varphi |= |\sigma |(\Omega ) \left\Vert \varphi \right\Vert _{C_0^1(\Omega )} \quad \text{ for } \text{ any } \varphi \in C_0^1(\Omega ). \end{aligned}$$

We define

$$\begin{aligned} A: C_0^1(\Omega ) \rightarrow C_0(\Omega ), \quad A(u)= \nabla u, \end{aligned}$$

which is a bounded linear operator, since

$$\begin{aligned} \left\Vert Au\right\Vert _{C_0(\Omega )}= &   \left\Vert \nabla u\right\Vert _{C_0(\Omega )} \le \left\Vert u\right\Vert _{C_0^1(\Omega )}\quad \text{ for } \text{ every } u \in C_0^1(\Omega ). \end{aligned}$$

Since $C_0^1(\Omega )$ is complete, the range *R*(*A*) of *A* is a closed subspace of $C_0(\Omega )$. We now define the functional

$$\begin{aligned} {\tilde{L}}:R(A) \rightarrow {\mathbb {R}}, \quad R(A) \subset C_0(\Omega ) \end{aligned}$$

as

$$\begin{aligned} {\tilde{L}}(\nabla u)= \sigma (u). \end{aligned}$$

We claim that ${\tilde{L}}$ is well defined.

Indeed, suppose that $\nabla u_1=\nabla u_2$. Then, on each connected component, $u_1=u_2 +C$ for some constant *C*. We recall that $u \in C_0^1(\Omega )$ if and only if both *u* and $\nabla u$ are continuous in $\Omega$ and, for any $\varepsilon >0$, there exists $K \Subset \Omega$ such that $|u(x)|\le \varepsilon$ and $|\nabla u(x)|\le \varepsilon$ for any $x \in \Omega \setminus K$. This characterization of $C_0^1(\Omega )$ implies that $C=0$ on each connected component.

We now show that ${\tilde{L}}$ is continuous on $R(A) \subset C_0(\Omega )$. For any $u \in C_0^1(\Omega )$, using the first part in (11.2), we compute

$$\begin{aligned} |{\tilde{L}}(\nabla u)|= |\sigma (u)|\le \left| {\sigma }\right| (\Omega ) \left\Vert u\right\Vert _{C_0(\Omega )}. \end{aligned}$$

By the Hahn-Banach Theorem, ${\tilde{L}}$ can be extended to a continuous linear functional

$$\begin{aligned} L:\,C_0(\Omega ) \rightarrow {\mathbb {R}}. \end{aligned}$$

Hence, by the Riesz Representation Theorem, there exists a unique ${\mathbb {R}}^n$-valued finite Radon measure ${{{\varvec{F}}}}=(F_1,F_2,\cdots ,F_n)$ such that

$$\begin{aligned} L(u)=\sum _{i=1}^{n} \int _{\Omega } u_i \,\textrm{d}F_i \quad \text {for any } u \in C_0(\Omega ). \end{aligned}$$

In particular, for any $\varphi \in C_{\textrm{c}}^{\infty }(\Omega )$, we have

11.4 $$\begin{aligned} \sigma (\varphi )= {\tilde{L}}(\nabla \varphi )= L(\nabla \varphi ) =\int _{\Omega } \nabla \varphi \cdot \, \textrm{d}{{\varvec{F}}}. \end{aligned}$$

Since the distributional divergence of ${{\varvec{F}}}$ is given by

11.5 $$\begin{aligned} \langle -\operatorname {div}{{\varvec{F}}}, \,\varphi \rangle = \int _{\Omega } \nabla \varphi \cdot \textrm{d}{{\varvec{F}}}, \end{aligned}$$

we conclude from (11.4)–(11.5) that

$$\begin{aligned} \langle -\operatorname {div}{{\varvec{F}}}, \,\varphi \rangle = \sigma (\varphi ) \quad \text {for any } \varphi \in C_{\textrm{c}}^{\infty }(\Omega ), \end{aligned}$$

that is, $$-\operatorname {div}{{\varvec{F}}}= \sigma $$. $$\square $$

### Remark 11.2

  A more classical method for solving equation (11.1) is to use first the Newtonian potential to solve the equation: $-\Delta u =\sigma$ and then define ${{\varvec{F}}}:= \nabla u$. This approach is used in [62, Example 3.3(i)], where it is shown that, if $\sigma$ has compact support in ${\mathbb {R}}^n$, a solution to (11.1) is given by ${{\varvec{F}}}(x) = -\frac{1}{n\omega _n} \int _{{\mathbb {R}}^n} \frac{\textrm{d}\sigma (y)}{|x - y |^{n-1}}$, where $\omega _n$ is the volume of the unit ball in ${\mathbb {R}}^n$. Note that, if $\sigma$ is a measure on a bounded domain $\Omega$, we can apply this by taking a zero extension of $\sigma$ to ${\mathbb {R}}^n$. Moreover, *u* belongs to ${W}^{1,p}_{\textrm{loc}}({\mathbb {R}}^n)$ for all $1 \le p < \frac{n}{n-1}$ so that ${{\varvec{F}}}= \nabla u \in L^{p}_{\text {loc}}({\mathbb {R}}^n, {\mathbb {R}}^n)$. If $\sigma =0$, the Newtonian potential approach clearly gives ${{\varvec{F}}}=0$.

Without the assumption of the measure having compact support, using similar techniques as in Theorem 11.1, it was shown in [53, Theorem 3.1] that, if $1 \le p \le \frac{n}{n-1}$ and $\sigma$ is any positive Radon measure in ${\mathbb {R}}^n$, equation (11.1) has a solution ${{\varvec{F}}}\in L^p({\mathbb {R}}^n,{\mathbb {R}}^n)$ if and only if $\sigma =0$. Moreover, it was indicated in [53, Theorem 3.2] that, for $\frac{n}{n-1}< p< \infty$, (11.1) has a solution ${{\varvec{F}}}\in L^p({\mathbb {R}}^n,{\mathbb {R}}^n)$ if and only if $I_1 \sigma \in L^p({\mathbb {R}}^n)$, where $I_1\sigma$ is the Riesz potential of order 1 of $\sigma$ defined as $I_1 \sigma (x)= \int _{{\mathbb {R}}^n} \frac{\textrm{d}\sigma (y)}{|x-y|^{n-1}}$. A characterization of this type is still unknown if $\sigma$ is a signed measure.

If $\sigma$ is represented by a function in $L^p$ for $1< p < \infty$, there exists a function $u \in W^{2,p}(\Omega )$ satisfying $-\Delta u =\sigma \in L^p (\Omega )$, and ${{\varvec{F}}}=\nabla u \in W^{1,p} (\Omega , {\mathbb {R}}^n)$ solves (11.1). For $p=1$, there are examples of functions $\sigma \in L^1(\Omega )$ for which there is no solution for (11.1) with ${{\varvec{F}}}\in W^{1,1}(\Omega ,{\mathbb {R}}^n)$ ([5, §2.1]). For $p =\infty$, there are functions $\sigma \in L^{\infty }(\Omega )$ for which there is no solution for (11.1) in $W^{1,\infty }(\Omega , {\mathbb {R}}^n)$ (see [5, §2.2] and [49]).

For the critical case $\sigma \in L^n(\Omega )$, even though there exists $u \in W^{2,n}(\Omega )$ that solves $-\Delta u=\sigma$, and hence $\nabla u \in W^{1,n}(\Omega , {\mathbb {R}}^n)$ solves (11.1), we can not conclude that $\nabla u \in L^{\infty } (\Omega , {\mathbb {R}}^n)$ since it is a limiting case of the Sobolev imbedding. Moreover, if we consider the function $u(x)= \varphi (x)x_1 |\ln |x||^{\alpha }, 0< \alpha < \frac{n-1}{n}$, where $\varphi$ is a smooth cut-off function with support near 0, then it holds that $\Delta u \in L^n$, but $\nabla u \notin L^{\infty }$ (see [5, Remark 7]). If $-\Delta u =\sigma \in L^p(\Omega )$ for $n< p < \infty$ and $u \in W^{2,p}(\Omega )$, then $\nabla u \in C^{0, 1-\frac{n}{p}}(\Omega , {\mathbb {R}}^n)$ (see [34, §5.6.2, Theorem 5]), which implies that, for the case $p=n$, we can not conclude that $\nabla u$ is continuous. Bounded and continuous vector fields that solve equation (11.1) with $\sigma \in L^n(\Omega )$ were constructed in Bourgain-Brezis [5, Proposition 1, Theorem 1, and Theorem 1’] by using other techniques.

For general distributions $\sigma ,$ a characterization of the solvability of (11.1) in the class of continuous vector fields was obtained in Pfeffer-De Pauw [32] and De Pauw-Torres [33]. It was shown in [32] that there exists a continuous vector field in $\Omega$ that solves the equation if and only if $\sigma$ belongs to a space of distribution denoted as the space of strong charges (see [32, Theorem 4.7]. In [33], it was proved that there exists a continuous vector field ${{\varvec{F}}}\in C_0({\mathbb {R}}^n,{\mathbb {R}}^n)$ (i.e., vanishing at infinity) if and only if $\sigma$ belong to the space of charges vanishing at infinity (see [33, Theorem 6.1]). The space $L^n$ belongs to both spaces of distributions, in particular solving equation (11.1) in the class of continuous vector fields when $\sigma \in L^n(\Omega )$.

Our construction in Theorem 11.1 provides a *characterization* of all fields ${{\varvec{F}}}$ such that $-\operatorname {div}{{\varvec{F}}}= \sigma$, as all possible extensions of ${{\tilde{L}}}$ from *R*(*A*) to $C_0(\Omega )$. Indeed, if ${{\varvec{F}}}$ is any such field, then, for any $\varphi \in C_{\textrm{c}}^{\infty }(\Omega )$,

$$\begin{aligned} \int _{\Omega } \nabla \varphi \cdot \textrm{d}{{\varvec{F}}}= \sigma (\varphi ) = {{\tilde{L}}}(\nabla \varphi ). \end{aligned}$$

Our proof relies on the Hahn-Banach Theorem to produce an extension and hence is non-constructive.

Similar techniques as in Theorem 11.1 have been used in [53, Theorem 4.5 and Theorem 3.5] and [54, Theorem 4.4 and Theorem 7.4] to obtain the necessary and sufficient condition on the measure $\sigma$ to solve $-\operatorname {div}{{\varvec{F}}}= \sigma$ in the spaces ${{\varvec{F}}}\in L^{\infty }(\Omega , {\mathbb {R}}^n)$ or ${{\varvec{F}}}\in C(\Omega ,{\mathbb {R}}^n)$.

## Equivalence between Entropy Solutions of Nonlinear PDEs of Divergence Form and the Mathematical Formulation of Physical Balance Laws

In this section, we employ the results obtained in Sects. 2–11 to establish the equivalence between solutions of nonlinear PDEs of divergence form and the mathematical formulation of physical balance laws, and to analyze entropy solutions of hyperbolic conservation laws.

### Mathematical Formulation of Physical Balance Laws

As stated in Sect. 6, a physical balance law on an open set $\Omega$ of ${\mathbb {R}}^n$ postulates that the *production* of a vector-valued extensive quantity in any bounded open set $U\Subset \Omega$ is balanced by the *Cauchy flux* of this quantity through the boundary $\partial U$ of the open set *U*; see also Dafermos [27] and the references cited therein.

Like the Cauchy flux, the *production* is introduced through a functional ${\mathcal {P}}$, defined on any bounded open set $U\Subset \Omega$, taking values in ${\mathbb {R}}^N$ and satisfying the conditions:

$$\begin{aligned}&{\mathcal {P}}(U_1\cup U_2)={\mathcal {P}}(U_1)+{\mathcal {P}}(U_2) \quad \text{ if } U_1\cap U_2=\emptyset ,\\&|{\mathcal {P}}(U)|\le {{\widetilde{\mu }}}(U), \end{aligned}$$

where ${\widetilde{\mu }}$ is a given Radon measure. It follows (from, for example, [42]) that ${\mathcal {P}}$ extends to a measure, in which there is a production measure $\sigma \in {\mathcal {M}}(\Omega ;{\mathbb {R}}^N)$, with $\sigma \ll {{\widetilde{\mu }}}$, such that

12.1 $$\begin{aligned} {\mathcal {P}}(U)= \sigma (U) \quad \text{ for } \text{ any } U \Subset \Omega \text{ open }. \end{aligned}$$

Then the physical principle of balance law on $\Omega$ can be mathematically formulated as

12.2 $$\begin{aligned} {\mathcal {F}}(\partial U)= {\mathcal {P}}(U) =\sigma (U) \end{aligned}$$

for any bounded open set $U\Subset \Omega$.

### Equivalence Between Weak Solutions of the Physical Balance Laws and Nonlinear PDEs of Divergence Form

First, combining Theorem 6.6 with (12.1)–(12.2), we conclude that there exists a unique divergence-measure field ${{\varvec{F}}}\in \mathcal{D}\mathcal{M}^{\textrm{ext}}(\Omega )$ such that

12.3 $$\begin{aligned} -\operatorname {div}{{\varvec{F}}}=\sigma \quad \text{ in } \text{ the } \text{ sense } \text{ of } \text{ distributions }, \end{aligned}$$

and, for any Borel subset $S\subset \partial U$ with $U\in {\mathcal {O}}_{\mu }$,

12.4 $$\begin{aligned} {\mathcal {F}}_U(S)=({{\varvec{F}}}\cdot \nu )_{\partial U}(S), \end{aligned}$$

where $({{\varvec{F}}}\cdot \nu )_{\partial U}$ is the normal trace of ${{\varvec{F}}}$ on $\partial U$. By considering a vector-valued flux and working component-wise, we can obtain a matrix-valued field ${{\varvec{F}}}= ({{\varvec{F}}}_1,{{\varvec{F}}}_2,\cdots ,{{\varvec{F}}}_N)^{\intercal }$ with each row ${{\varvec{F}}}_i$ lying in $\mathcal{D}\mathcal{M}^{{{\,\textrm{ext}\,}}}(\Omega )$, so (12.3) becomes a *system* of balance laws (understood with a row-wise divergence).

Consider the state of the medium under consideration that is described by a state vector field ${\textbf{u}} = (u_1,\cdots , u_N)^{\intercal }$ taking values in ${\mathbb {R}}^N$, which determines both the flux density field ${{\varvec{F}}}$ and the production density field $\sigma$ at point $y\in \Omega$ by the constitutive relations:

12.5 $$\begin{aligned} {{\varvec{F}}}(y)= {{\varvec{F}}}({\textbf{u}}(y),y), \quad \sigma (y):=\sigma ({\textbf{u}}(y), y), \end{aligned}$$

where ${{\varvec{F}}}({\textbf{u}},y)$ and $\sigma ({\textbf{u}}, y)$ are given vector fields in $({\textbf{u}},y)$, understood in a sense determined by the physical system.

In the case that ${{\varvec{F}}}({\textbf{u}},y)$ and $\sigma ({\textbf{u}},y)$ are sufficiently regular, (12.5) is always well-defined if ${\textbf{u}}\in L^{\infty }$. This is not always so if ${\textbf{u}}\in L^p$ for some $1 \le p < \infty$, and further assumptions may be necessary to ensure the composition ${{\varvec{F}}}({\textbf{u}}(y),y)$ is locally integrable. If ${\textbf{u}}$ is measure-valued, then we must give a meaning to (12.5); this is a modeling issue, which is beyond the scope of this paper. An example of such constitutive relations in the measure-valued case is given in [11] for the Euler equations for gas dynamics in Lagrangian coordinates.

Combining (12.3) with (12.5), we obtain the first-order quasilinear system of PDEs of divergence form:

12.6 $$\begin{aligned} \operatorname {div}{{\varvec{F}}}({\textbf{u}}(y), y)+\sigma ({\textbf{u}}(y), y)=0, \end{aligned}$$

which is called a system of nonlinear PDEs of balance laws (*cf*. [27]).

For the zero-production case: ${\mathcal {P}}=0$, which implies that $\sigma ({\textbf{u}}(y), y)=0$, then the above derivation yields

12.7 $$\begin{aligned} \operatorname {div}{{\varvec{F}}}({\textbf{u}}(y), y)=0, \end{aligned}$$

which is called a nonlinear system of conservation laws. In particular, when the medium is homogeneous:

$$\begin{aligned} {{\varvec{F}}}({\textbf{u}},y)={{\varvec{F}}}({\textbf{u}}), \end{aligned}$$

depending on *y* only through the state vector ${\textbf{u}}={\textbf{u}}(y)$, then system (12.7) becomes

$$\begin{aligned} \operatorname {div}{{\varvec{F}}}({\textbf{u}}(y))=0. \end{aligned}$$

Now suppose that the coordinate system *y* is described by the time variable *t* and the space variable $x=(x_1,\cdots , x_m)$:

$$\begin{aligned} y=(t,x)=(t,x_1,\cdots , x_m), \quad n=m+1, \end{aligned}$$

and the flux density is written as an $N \times n$ matrix:

$$\begin{aligned} {{\varvec{F}}}({\textbf{u}})=({\textbf{u}}, {\textbf{f}}({\textbf{u}}))=({\textbf{u}}, {\textbf{f}}_{1}({\textbf{u}}), \cdots , {\textbf{f}}_{m}({\textbf{u}})) \end{aligned}$$

where ${\textbf{f}}_i^{\intercal } :{\mathbb {R}}^N \rightarrow {\mathbb {R}}^m$ for each $i = 1,\cdots ,m$. Then we obtain the following standard form for the system of conservation laws:

12.8 $$\begin{aligned} \partial _t {\textbf{u}}+\operatorname {div}_x {\textbf{f}}({\textbf{u}})=0, \quad (t,x)\in {\mathbb {R}}^n, \,\, {\textbf{u}}\in {\mathbb {R}}^N, \end{aligned}$$

where the spacial divergence is taken row-wise.

This shows the equivalence between the weak solutions of physical flows governed by the balance laws (12.2) and weak solutions of the corresponding system of nonlinear PDEs of divergence form (12.6), or even a system of conservation laws (12.8), through the relations (12.3)–(12.4).

#### Theorem 12.1

The following statements hold:

(i) The state variables ${\textbf{u}}\in {\mathbb {R}}^N$ (even measure-valued) that are governed respectively by the physical balance laws (12.2) and the corresponding nonlinear PDEs of divergence form (12.6) are equivalent on any open set *U*. That is, the weak solutions ${\textbf{u}}\in {\mathbb {R}}^N$ (even measure-valued) for the state variables of the physical balance laws (12.2) and the corresponding nonlinear PDEs of divergence form (12.6) are equivalent.
(ii) Given an open set *U*, the divergence-measure field ${{\varvec{F}}}\in \mathcal{D}\mathcal{M}^{\textrm{ext}}(\Omega )$ is endowed with the normal trace $({{\varvec{F}}}\cdot \nu )_{\partial U}$ on $\partial U$ such that (12.4) hold for any Borel subset $S\subset \partial U$ with $U\in {\mathcal {O}}_{\mu }$.
(iii) If (12.7) is satisfied, across any discontinuity surface *S* on which the underlying field ${{\varvec{F}}}$ does not concentrate, the weak Rankine-Hugoniot condition holds: The exterior and interior normal traces of the divergence-measure field ${{\varvec{F}}}$ for weak solutions are equal, i.e., the normal trace is continuous across *S*.

However, the exterior and interior normal traces of the corresponding entropy divergence-measure fields on a shock wave must have a jump, inferred by the second law of thermodynamics as indicated in Sect. 12.3 below.

### Entropy Solutions of Hyperbolic Conservation Laws

We now apply the results established in Sect. 4 through Sect. 9 to the recovery of Cauchy entropy fluxes and the corresponding entropy balance laws through the Lax entropy inequality (i.e., the second law of thermodynamics) for entropy solutions of hyperbolic conservation laws by capturing entropy dissipation. That is, for hyperbolic conservation laws, even though there may be no production for a weak solution, the entropy solutions must obey the entropy balance law with non-zero production in general, especially when the entropy solution contains shock waves. This leads to the intrinsic connection between the second law of thermodynamics and the entropy balance laws in Continuum Mechanics.

We focus now on system (12.8), which is assumed to be hyperbolic. A function $\eta : {\mathbb {R}}^N\rightarrow {\mathbb {R}}$ is called an entropy of (12.8) if there exists ${\textbf{q}}= ({q}_1,\cdots ,{q}_m)^{\intercal }: {\mathbb {R}}^N\rightarrow {\mathbb {R}}^m$ such that

$$\begin{aligned} \nabla q_j({\textbf{u}})=\nabla \eta ({\textbf{u}}) \nabla {\textbf{f}}_j({\textbf{u}}), \quad j=1,\cdots , m. \end{aligned}$$

Then the vector function ${\textbf{q}}({\textbf{u}})$ is called an entropy flux associated with entropy $\eta ({\textbf{u}})$, and the function pair $(\eta ({\textbf{u}}), {\textbf{q}}({\textbf{u}}))$ is called an entropy pair. The entropy pair $(\eta ({\textbf{u}}), {\textbf{q}}({\textbf{u}}))$ is called a convex entropy pair on the state domain $D\subset {\mathbb {R}}^N$ if the Hessian matrix $\nabla ^2\eta ({\textbf{u}})\ge 0$ for any ${\textbf{u}}\in D$.

It is observed that most systems of conservation laws that result from continuum mechanics are endowed with a globally defined convex entropy (see Dafermos [27] and Friedrichs-Lax [41]). The available existence theories show that solutions of (12.8) generally fall within the following class of entropy solutions:

#### Definition 12.2

  A vector function ${\textbf{u}}={\textbf{u}}(t,x)\in {\mathcal {M}}_{\textrm{loc}}({\mathbb {R}}_+\times {\mathbb {R}}^m)$ or $L^p({\mathbb {R}}_+\times {\mathbb {R}}^m)$ for some $p\ge 1$ is called an entropy solution of (12.8) if ${\textbf{u}}(t,x)$ satisfies the Lax entropy inequality:

12.9 $$\begin{aligned} \partial _t\eta ({\textbf{u}}(t,x))+\operatorname {div}_x {\textbf{q}}({\textbf{u}}(t,x))\le 0 \end{aligned}$$

in the sense of distributions for any convex entropy pair $(\eta , {\textbf{q}})$ such that

$$\begin{aligned} (\eta ({\textbf{u}}(t,x)),\,{\textbf{q}}({\textbf{u}}(t,x))) \quad \text{ is } \text{ a } \text{ distributional } \text{ field }. \end{aligned}$$

Clearly, an entropy solution is a weak solution, which can be seen by choosing $(\eta ({\textbf{u}}), {\textbf{q}}({\textbf{u}}))=\pm (u_i, {\textbf{f}}_i({\textbf{u}}))$, $i = 1,\cdots ,N$, in (12.9).

One of the main issues in hyperbolic conservation laws is to study the behavior of entropy solutions in this class to explore to the fullest extent possible all questions relating to uniqueness, stability, large-time behavior, structure, and traces of entropy solutions, with neither specific reference to any particular method for constructing the solutions nor additional regularity assumptions. The Lax entropy inequality (12.9) indicates that the distribution:

$$\begin{aligned} \partial _t\eta ({\textbf{u}}(t,x))+\operatorname {div}_x {\textbf{q}}({\textbf{u}}(t,x)) \end{aligned}$$

is nonpositive. Then we conclude that it is, in fact, a Radon measure; that is, there exists $\sigma _\eta \in {\mathcal {M}}({\mathbb {R}}_+\times {\mathbb {R}}^m)$ with $\sigma _\eta \ge 0$ such that

$$\begin{aligned} -\operatorname {div}_{(t,x)}(\eta ({\textbf{u}}(t,x)), {\textbf{q}}({\textbf{u}}(t,x)))=:\sigma _\eta . \end{aligned}$$

Therefore, the vector field $(\eta ({\textbf{u}}(t,x)), {\textbf{q}}({\textbf{u}}(t,x)))$ is a divergence-measure field:

$$\begin{aligned} (\eta ({\textbf{u}}(t,x)), {\textbf{q}}({\textbf{u}}(t,x)))\in {\mathcal {D}}{\mathcal {M}}^{\textrm{ext}}_{\textrm{loc}}({\mathbb {R}}_+\times {\mathbb {R}}^m), \end{aligned}$$

provided that $$(\eta ({\textbf{u}}(t,x)), {\textbf{q}}({\textbf{u}}(t,x)))\in {\mathcal {M}}_{\textrm{loc}}({\mathbb {R}}_+\times {\mathbb {R}}^m)$$.

We introduce a functional on any surface $S\subset \partial U$ with $U\in {\mathcal {O}}_\mu$:

12.10 $$\begin{aligned} {\mathcal {F}}_U^\eta (S):=-((\eta ({{\textbf {u}}}), {{\textbf {q}}}({{\textbf {u}}}))\cdot \nu )_{\partial U}(S), \end{aligned}$$

where $((\eta ({\textbf{u}}), {\textbf{q}}({\textbf{u}}))\cdot \nu )_{\partial U}$ is the normal trace of $(\eta ({\textbf{u}}), {\textbf{q}}({\textbf{u}}))$ on $\partial U$ in the sense of Theorem 3.3, since $(\eta ({\textbf{u}}(t,x)), {\textbf{q}}({\textbf{u}}(t,x)))\in {\mathcal {D}}{\mathcal {M}}^{\textrm{ext}}_{\textrm{loc}}({\mathbb {R}}_+\times {\mathbb {R}}^m)$.

By Theorem 6.7, the functional ${\mathcal {F}}_U^\eta$ defined by (12.10) is a Cauchy flux in the sense of Definition 6.4, taking $\mu$ to be the total variation measure $|(\eta ({\textbf{u}}(t,x)), {\textbf{q}}({\textbf{u}}(t,x))|$.

#### Definition 12.3

(Cauchy entropy fluxes)  A functional ${\mathcal {F}}_U^\eta$ as defined by (12.10) is called a Cauchy entropy flux with respect to entropy $\eta ({\textbf{u}})$.

In particular, when $(\eta , {\textbf{q}})$ is a convex entropy pair, ${\mathcal {F}}_U^\eta (S)\ge 0$ for any Borel set $S\subset \partial U$ with $U\in {\mathcal {O}}_\mu$. Furthermore, we can now reformulate the balance law of entropy from the recovery of entropy production by capturing entropy dissipation.

#### Theorem 12.4

(Entropy balance laws)  The production $\sigma _\eta$ of entropy dissipation in any bounded open set $U\Subset \Omega$ is balanced by the entropy Cauchy flux ${\mathcal {F}}_U^\eta$ through the boundary $\partial U$ of the open set $U\Subset \Omega$.

Furthermore, we have

#### Theorem 12.5

  Assume that ${\textbf{u}}={\textbf{u}}(t,x)\in {\mathcal {M}}_{\textrm{loc}}({\mathbb {R}}_+\times {\mathbb {R}}^m)$ or $L^p({\mathbb {R}}_+\times {\mathbb {R}}^m)$ for some $p\ge 1$ is an entropy solution to (12.8). Then

(i) Across the discontinuity, the normal trace of the vector field $({\textbf{u}}(t,x), {\textbf{f}}({\textbf{u}}(t,x)))$ is continuous, provided the underlying field does not concentrate on the discontinuity surface; in this case the weak Rankine-Hugoniot condition holds.
(ii) For a convex entropy pair $(\eta ({\textbf{u}}), {\textbf{q}}({\textbf{u}}))$ with $\nabla ^2\eta ({\textbf{u}})>0$, the normal trace of the vector field $(\eta ({\textbf{u}}(t,x)), {\textbf{q}}({\textbf{u}}(t,x)))$ has a jump across a shock wave, which decreases across the shock in the *t*-direction.

The continuity of the normal trace of the vector field $({\textbf{u}}(t,x), {\textbf{f}}({\textbf{u}}(t,x)))$ is due to the fact that $\operatorname {div}({\textbf{u}}(t,x), {\textbf{f}}({\textbf{u}}(t,x)))$ is zero and $|({\textbf{u}},{\textbf{f}}({\textbf{u}}))|(\partial U) = 0$ as assumed. On the other hand, the jump of the normal trace of the vector field $(\eta ({\textbf{u}}(t,x)), {\textbf{q}}({\textbf{u}}(t,x)))$ is because $\operatorname {div}(\eta ({\textbf{u}}(t,x)), {\textbf{q}}({\textbf{u}}(t,x)))$ has a concentration on a shock wave, due to the entropy dissipation, concentrated on the shock wave.

Furthermore, for characteristic discontinuities such as vortex sheets and entropy waves, in general, $\operatorname {div}(\eta ({\textbf{u}}(t,x)), {\textbf{q}}({\textbf{u}}(t,x)))$ does not have a concentration due to the loss of the entropy dissipation along such a discontinuity, and the normal trace for the vector field $(\eta ({\textbf{u}}(t,x)), {\textbf{q}}({\textbf{u}}(t,x)))$ may generally be continuous across the characteristic discontinuity normally.

Moreover, it is clear that understanding further properties of divergence-measure fields can advance our understanding of the behavior of entropy solutions for hyperbolic conservation laws and other related nonlinear PDEs by selecting appropriate entropy pairs. As examples, we refer the reader to [8–18, 65] for such applications.

## Data Availability

Data sharing is not applicable to this article as no datasets were generated or analyzed during the current study.
